# Evaluation of the Suitability of an Existing Job–Exposure Matrix for the Assessment of Exposure of UK Biobank Participants to Dust, Fumes, and Diesel Exhaust Particulates

**DOI:** 10.3390/ijerph17144919

**Published:** 2020-07-08

**Authors:** Eirini Dimakakou, Helinor J. Johnston, George Streftaris, John W. Cherrie

**Affiliations:** 1 School of Engineering and Physical Sciences, Institute of Biological Chemistry, Biophysics and Bioengineering, Heriot-Watt University, Edinburgh EH14 4AS, UK; h.Johnston@hw.ac.uk; 2 Maxwell Institute for Mathematical Sciences, School of Mathematical and Computer Sciences, Heriot-Watt University, Edinburgh EH14 4AS, UK; G.Streftaris@hw.ac.uk; 3 Institute of Occupational Medicine (IOM), Edinburgh EH14 4AP, UK

**Keywords:** particulate air pollutants, UK Biobank, occupational exposure, dust, fumes, diesel exhaust particulate, Job–Exposure Matrix, JEM, ACE JEM, epidemiology

## Abstract

Many epidemiological studies have shown an association between outdoor particulate air pollutants and increased morbidity and mortality. Inhalation of ambient aerosols can exacerbate or promote the development of cardiovascular and pulmonary diseases as well as other diseases, such as type 2 diabetes mellitus (T2DM) and neurodegenerative diseases. Occupational exposure to dust, fumes and diesel exhaust particulates can also cause adverse health outcomes and there are numerous occupations where workers are exposed to airborne particles that are similar to ambient air pollution. An individual’s job title has normally been identified as a major determinant of workplace exposure in epidemiological studies. This has led to the development of Job–Exposure Matrices (JEMs) as a way of characterising specific workplace exposures. One JEM for airborne chemical exposures is the Airborne Chemical Exposure Job–Exposure Matrix (ACE JEM), developed specifically for the UK Biobank cohort. The objective of this paper is to evaluate the suitability of the ACE JEM in assessing occupational aerosol exposure of participants in the UK Biobank. We searched the scientific literature to identify exposure data linked to selected jobs in the ACE JEM and compared these data with the JEM assessments. Additionally, we carried out an independent expert-based assessment of exposure to compare with the JEM estimates. There is good published evidence to substantiate the high dust and biological dust assignments in the JEM and more limited evidence for diesel exhaust particulates. There is limited evidence in the published literature to substantiate moderate or low exposure assignments in the JEM. The independent expert-based assessment found good agreement at the two extremes of exposure in the JEM (high and no exposure), with uncertainty in all other classifications. The ACE JEM assignments are probably reliable for highly exposed jobs and for jobs assigned as unexposed. However, the assignments for medium and low exposures are less reliable. The ACE JEM is likely to be a good tool to examine associations between occupational exposures to particulates and chronic disease, although it should be used with caution. Further efforts should be made to improve the reliability of the ACE JEM.

## 1. Introduction

Many epidemiological studies have shown an association between outdoor particulate air pollutants and increased morbidity and mortality (reviewed by Johnston et al. [1]). More specifically, it is established that inhalation of airborne particulates can exacerbate or cause cardiovascular and pulmonary diseases [2,3]. This is observed globally as many countries suffer from poor air quality. For example, there are studies using data from the UK Biobank cohort (a large population study that examines how environmental, genetical and lifestyle factors affects human health) that have shown associations between outdoor particulate air pollution and chronic obstructive pulmonary disease (COPD) and difficulties in breathing [4,5]. There is increasing evidence for associations of environmental particulate matter (PM) exposure with other diseases, including type 2 diabetes mellitus (T2DM) and neurodegenerative diseases, such as Alzheimer’s disease and Parkinson’s disease [6,7,8,9].

Occupational exposure to dust, fumes and particulates can also aggravate or cause respiratory diseases and other adverse health outcomes, such as decreased lung function, occupational asthma, ischaemic heart disease and cancer [10,11,12,13,14]. However, corresponding evidence that workplace particle exposures can cause T2DM or neurodegenerative diseases is lacking, and further research is therefore needed to investigate these risks in occupational populations. The UK Biobank cohort could offer a powerful basis for such studies.

There are numerous occupations (e.g., construction workers and carpenters) where workers are exposed to airborne particles, such as mineral dusts, metal and polymer fumes, and ultrafine particles. These particles (e.g., diesel exhaust particulates and carbon black particles) have similar physicochemical properties (e.g., size) to ambient particulate air pollution, and so there is concern that exposure to these particles in an occupational setting will cause similar adverse health outcomes. The physicochemical properties of particles are known to influence their toxic potency. For many workplace aerosols, the hazard arises mainly from the chemical composition or the nature of the dust (e.g., arsenic and crystalline silica), or the shape and durability of particles, such as asbestos. The size of the particles may also determine the toxicity [15], as it is likely that smaller particles (<100 nm) will give rise to greater toxicity than larger particles of the same chemical composition due to their larger surface area [16,17].

An individual’s job title has been identified as a major determinant of workplace exposure in epidemiological studies. Workers with the same job (e.g., carpenter or maintenance mechanic) often have shared work exposures in epidemiology studies regardless of where they work. This has led to the development of Job–Exposure Matrices (JEMs) as a way of characterising specific workplace exposures for epidemiology studies. A JEM allows for the assessment of exposure for individual workers without the need for further specific details of the work activities or environment [18]. The assessments can be combined for different jobs throughout a lifetime to provide an estimate of cumulative exposure. JEMs are very useful where there is a diversity of occupations in the study population, such as in a population-based case-control study, as they provide a quick way to transform coded occupational titles into potential exposures. However, their simplicity is their main limitation as they cannot take into account the variability in exposure between workplaces or between workers, both of which can be large. Additionally, JEMs are often derived from expert judgement rather than objective data on workplace exposures and there may be little evidence to substantiate the categorization [19]. These limitations can lead to misclassification bias in epidemiological analyses [20]. For example, JEMs based on self-reports and expert consensus had higher odds ratios than JEMs derived from measurement data, which suggests that the use of JEMs should be made with caution in order to estimate the exposures correctly [21].

A JEM using the UK Standard Occupational Classification (SOC) 2000 system (i.e., the Airborne Chemical Exposure Job–Exposure Matrix (ACE JEM)), has been developed to investigate workplace causes of chronic obstructive pulmonary disease (COPD) amongst participants in the UK Biobank [22], which is a large UK population-based study with good-quality environmental and lifestyle data. This expert-derived JEM was developed for a range of different airborne workplace pollutants, such as dust, fumes and diesel exhaust particulates [22]. Although the ACE JEM has not been critically evaluated to assess its reliability, it has shown its utility in identifying associations between specific workplace exposures and COPD [23].

The objective of this study was to evaluate the suitability of the ACE JEM in assessing occupational aerosol exposure of participants in the UK Biobank cohort to airborne dust, fumes and diesel exhaust particulates, in order to assess how it could best be applied to study a range of adverse health outcomes (e.g., neurological diseases, T2DM), and to identify whether it needs to be improved.

## 2. Materials and Methods

Two approaches were employed to assess the suitability of the ACE JEM for jobs typically found in the UK Biobank study. Firstly, we searched the scientific literature to identify exposure data linked to these jobs and compared these data with the JEM assessments; secondly, we carried out an independent expert-based assessment of the JEM coding.

### 2.1. Step 1: Extraction of Information from ACE JEM and UK Biobank (Tables 1 to 5)

For each SOC code in the JEM with exposure to dust, mineral dust, biological dust, fumes or diesel exhaust particulate assigned, the data on the assessed average level of exposure for exposed individuals and the proportion of the population exposed was extracted from the ACE JEM. Then, from the UK Biobank, information based on the employment history (Job SOC coding, variable 22617) [24] was extracted, and in particular the number of people that had been employed in each occupation. Based on the latter information, the five most common occupations from each exposure intensity category for each pollutant type (high, medium, low and unexposed) were selected for further evaluation and information about the number of people in these different occupations was extracted from the UK Biobank (Table 1, Table 2, Table 3, Table 4 and Table 5). Finally, information about the proportion exposed to each pollutant type for these occupations was also extracted from the ACE JEM. The proportion exposed is divided in four categories: 0–5%, 5–19%, 20–49% and ≥50%.

### 2.2. Step 2: Searching the Scientific Literature (Table 6 and Tables S6–S10)

The JEMs are tools that convert information on jobs that were collected in epidemiological studies into information on potential exposures [25]; therefore, they are based on the existing literature. As a consequence, to assess a matrix, it is a prerequisite to scan, investigate and compare the existing research. Therefore, we searched the literature to gather information for each of the identified occupations and the corresponding exposures from Step 1. Quantitative and qualitative information was collected to assess the exposures to each pollutant type in each occupation. PubMed and Scopus databases were used for the literature searches using different combinations of search terms related to the occupation and the type of exposure of interest, and then titles and abstracts were examined. Studies were included if they were written in English and were carried out in countries with similar workplace conditions and laws as in UK to help ensure comparability with the UK Biobank population. The search terms used are listed in the Supplementary Material.

### 2.3. Step 3: Expert Estimation of the Exposure (Table S11 and Table 8)

A list of all the occupations identified in Step 1 was created, along with the standard job description published with the SOC codes [26]. A blind estimation of the intensity and proportion of exposed workers was then carried out by an expert. Cohen’s kappa, calculated using the R package, was used to assess agreement between the rater and the JEM assignments [27].

## 3. Results

### 3.1. ACE JEM and UK Biobank Information about the Exposure

According to the combined information from the ACE JEM and the UK Biobank, labourers in process and plant operations are the most common occupation (5223 participants, which is about 2% of the UK Biobank population) with high dust and mineral dust exposure and moderate fume exposure. Carpenters and joiners are the second most common dust exposed occupation and the most common high biological dust exposed occupation. Labourers in building and woodworking trades are highly exposed to both dust and mineral dust, but their exposure to biological dust and fumes is moderate (medium). Construction operatives are exposed in high levels to both dust and mineral dust and the construction trade is an occupation that is highly exposed to dust. Generally, we observe that construction workers and people working in the construction industry are mostly highly exposed to dust and biological dust.

Moderate dust and mineral dust exposure was common in metal working production and maintenance fitters, though they have low exposure to fumes. People that have in the past worked as motor mechanics and auto engineers have moderate exposure to dust, mineral dust, fumes and diesel. Cleaners and domestics have medium average exposure to dust and mineral dust. Farm workers and farmers, which comprise about 0.5% of the UK Biobank population, have moderate exposure to mineral dust, biological dust, and dust in general, according to the ACE JEM. People that worked as painters and decorators are exposed to moderate levels of dust and mineral dust.

Nurses are assigned low dust and biological dust exposure and are entirely unexposed to diesel, fumes, and mineral dust according to the matrix. Laboratory technicians have low mineral, biological and general dust exposure. NCOs and other ranks are occupations that were found in the most crowded occupations in the UK Biobank and have low exposure to dust, mineral dust, diesel and fumes. Both science/engineering technicians and medical practitioners are exposed to low levels of dust, but the first group is also exposed to low levels of mineral dust, whereas the second to the same levels of biological dust. Therefore, generally, scientific and medical occupations are in the low exposure category.

Ship and hovercraft officers as well as the garage managers and proprietors are exposed to medium levels of diesel exhaust particulates. Coalmine operatives are also exposed to medium levels of diesel, as are fire service officers, but are also highly exposed to mineral dust according to the JEM, whereas the fire service officers are also highly exposed to fumes. Mechanical engineers and police officers are exposed to a small amount of diesel and fumes and both sales representatives and managers in construction are exposed to low levels of diesel exhaust.

It is also noticeable that there is no job in the UK Biobank population that is identified as highly exposed in ACE JEM for diesel exhaust (see Table 3). People working as sheet metal workers, moulders, core makers, die casters, smiths, forge workers and people who work in welding trades are highly exposed to fumes, whereas electricians, electronic technicians, chefs and cooks are not that exposed to fumes (medium exposure). The least exposure to fumes is found for assemblers and routine operatives, although they are also exposed to low levels of mineral dust.

Food, drink and tobacco process operatives, bakers and flour confectioners, furniture makers and other craft woodworkers are highly exposed to biological dust. The same applies to textile process operatives, who are also exposed to high levels of mineral dust. Weighers, graders and sorters at assembling and routine operation and paper and wood machine operatives have a mediocre exposure to biological dust and nursing auxiliaries, care assistants and home carers have a low biological dust exposure.

Occupations such as secondary education teaching and primary and nursery education teaching professionals, personal assistants and secretaries and finally local government clerical officers and assistants were totally unexposed to all the five materials, which might imply that the scientific occupations are mostly unexposed to the above.

The estimated proportion exposed to pollutants according to the ACE JEM is that most of the high and medium exposed occupations are more than 50% exposed. For the low exposed occupations, only 5–19% are judged exposed, except for the assemblers and routine operatives, where more than 50% are exposed to low levels of mineral dust and 20–49% exposed to low levels of fumes. Also, 20–49% of nurses, medical practitioners and mechanical engineers are judged exposed to low levels of biological dust and diesel, respectively.

### 3.2. Literature Information about the Exposure

Generally, more papers were identified for the high exposed occupations, less for the medium exposed and only a few papers for the low and the unexposed occupations.

Abundant evidence was found for construction operatives, carpenters and joiners, furniture makers and craft woodworkers, and farmers and bakers for dust and biological dust exposure in their work environment. Carpenters and joiners have high exposure to dust and biological dust in the ACE JEM and the data collected substantiates that exposures are high, as there are 37 papers of dust exposure and 29 papers for biological dust exposure (Table 6 and Supplementary Material (SM) references 2–38) and that seems consistent with the ACE JEM. They are exposed to wood dust mostly, which is classified as a biological dust. The wood dust levels in these occupations have been measured and typically exceed national exposure limits. They have been identified to cause several health problems, such as asthma, respiratory inflammation, and cancer. Construction operatives, who have high assessed exposure to dust and mineral dust according to the ACE JEM, are exposed to respirable dust, wood dust, asbestos, diesel, and mineral dust, such as quartz exposure based on the published data, where 60% of the dust literature is about construction operatives. Sometimes, those levels can exceed the limit values for dust and quartz exposure. Adverse health outcomes mentioned in the literature include respiratory symptoms, a decline in lung function, COPD, silicosis, ischaemic heart disease and cancer (SM references 31, 43, 45–115, 395–400). Bakers and flour confectioners, who are highly exposed to biological dust in the ACE JEM, have also been identified as exposed to biological dust from the literature; from the published data, 36 papers were found mentioning their exposure to flour dust, airborne moulds, soybean dust and dust mites that can lead to nasal mucosal inflammation, asthma, allergic obstructive airway disease, immunological disorders and pulmonary function impairment (SM references 261–296). The measured inhalable dust levels in these occupations sometimes exceed 10 mg/m^3^, which is the workplace exposure limit for such exposures in the UK [28]. Furniture makers and other craft woodworkers are exposed to organic wood dust and the dust exposure could be 3.75 mg m^−3^ years (SM reference 278).

There is also considerable evidence that the exposure levels for farm workers and farmers is appropriate in the ACE JEM as 82 papers substantiate exposure to dust and biological dust. These workplaces are exposed to inorganic and organic dust, according to the literature (SM references 140–158, 334–394). They are exposed to PM, endotoxins and air pollutants. More than 50% of the dust measurements for endotoxins and organic dust were reported to exceed the recommended health occupational exposure limits.

Moreover, people that work in metal production and maintenance fitting are exposed to mixed manganese, cadmium, cobalt, nickel and chromium dust, as well as airborne contaminants, with 14 papers supporting this (SM references 120–131, 402–403). Dust exposure levels can be anywhere from 0.001 to 83 mg/m^−3^. There is also enough evidence for diesel exposure and gasoline emissions for motor mechanics, auto engineers and coal mine operatives, and there is also adequate evidence about cooking fumes for chefs and cooks.

Therefore, there is good published evidence for the reliability for the JEM assessments for dust and biological dust and more limited evidence for diesel exhaust particulates, but for all the other exposures, there is poor evidence in the published literature for the JEM assignments.

### 3.3. Comparison between an Independent Exposure Assessor and the ACE-JEM

The comparison between our estimations and the ACE JEM’s estimations for biological dust levels of exposure revealed the highest concordance (Cohen’s κ = 0.58, percentage of agreement 74%) among all exposures (Table 7). Fumes and diesel exposure both had the same agreement percentage (67%) and Cohen’s κ (0.52 and 0.42, respectively). The lower value of κ for diesel exposure reflects the fact that the kappa statistic also takes into account potential agreement between the raters occurring by chance [29]. For mineral dust exposure levels Cohen’s κ was 0.22, showing also the lowest over all agreement between the two comparisons (51%), while for dust levels, Cohen’s κ was 0.44 and there was 58% agreement between the two assessments. In addition, we graphically present the two assessors’ classification of each exposure type in different levels (Figure S1). Each figure shows the cross-tabulation of the assessors’ classification, with the given numbers corresponding to the percentage of cases classified by the two assessors in a given combination of levels (0–3). For dust exposure, there is a similar agreement across all intensity levels (high, medium, low, unexposed) with around 12–16% agreement at each level, while for all other exposures, the agreement appears to be mainly from the non-exposed (0) level (ranging from 37% to 56%). The entries in the main diagonal of each table indicate agreement between the two assessments, while the non-diagonal elements signify non-agreement. Therefore, for mineral dust exposure, we observe that there is low consistency between the two raters.

We also compare our estimations of the proportions exposed for each type of exposure to the ACE JEM corresponding estimates. The highest agreement percentage is observed in diesel and bio dust, with 67% and 65% respectively (Cohen’s κ = 0.40 for both—Table 7). The agreement for the other three types of exposure (dust, fumes and mineral dust) was 56% and Cohen’s kappa coefficient was 0.39, 0.30 and 0.28 respectively. These statistics suggest that there is limited agreement between the two raters for the proportions of workers exposed to fumes and mineral dust. We can also verify these findings from the cross-tabulated entries in the tables in Figure S2, where we can notice that the two evaluations agree more on diesel and biological dust.

Table 8 shows the classification from the two assessments (our estimations and ACE JEM’s estimations) for both level of exposure and proportions exposed. The table reveals that there is, in general, positive agreement between the two ratings in the block diagonal and predominantly at the two extremes (non-exposed/zero proportion, high exposure/high proportion), while there is greater uncertainty in the classifications in between.

## 4. Discussion

Whilst there is no gold standard available to retrospectively assess work exposure to dusts and other aerosols, the exposure assessment can be undertaken using JEMs [19]. Although JEMs are not perfect, they are a convenient tool and have been shown to be capable of converting coded occupations into potential exposures [20] and identifying known associations between exposure and disease. This work aimed to assess the reliability of the ACE JEM, which was created to assess occupational exposure of participants in the UK Biobank cohort, to assess risks of lung disease where the main exposure is inhalation of aerosols.

To be able to assess the JEM in this study, we have chosen a sample of occupations that were commonly carried out by the participants in the UK Biobank. We investigated those occupations and their exposure to a range of pollutants by searching the literature for scientific evidence based on epidemiological, exposure and other studies (e.g., systematic reviews) for similar exposures. Then, we independently assessed the level and proportion of workers exposed for a selection of jobs commonly encountered in the UK Biobank cohort. As observed from information about exposure from the published literature, there was a lot of evidence for categories such as high dust and biological dust, but information was mostly missing for all the other categories. Thus, the only way to investigate the reliability of the JEM and further explore if we should rely on the occupations with insufficient evidence was to use expert judgement in order to investigate all categories. Therefore, we used two approaches to assess the JEM: literature evidence and independent expert assessment. Both approaches agreed that there is sufficient evidence to substantiate the JEM assignments for the high exposed occupations but no secure evidence for the unexposed. Therefore, the two methods used complement and support each other, but also bring out the problem of the lack of evidence for all the other categories.

More specifically, based on the literature search, we observed that for the jobs that had high exposure categories in the ACE JEM, there was published evidence to substantiate the assignment, particularly for dust, fumes, diesel exhaust particulate, biological and mineral dust. There was limited evidence for many of the high exposed groups having exposure to fumes and mineral dust, and there was almost no evidence for the low exposed groups and no published evidence for the unexposed. In some highly exposed jobs, such as carpenters, joiners, construction operatives, bakers and craft woodworkers, many studies were found, several of which also provided information about the level of exposure. The medium exposure had some equivocal results. Some occupations, such as farm workers and farmers, chefs and cooks, and motor mechanics and auto engineers, had enough evidence supporting the occupational exposures in the different pollutants, but there were also other occupations, such as labourers in buildings and process, fire service officers, and painters and decorators, with no supporting evidence from the literature. The results from searching the literature, suggest that the boundaries between the four categories (high, medium, low, and unexposed) are blurred. There are occupations that according to the ACE JEM are highly exposed, but the literature shows insufficient evidence for this, i.e., textile process operatives, who seem to be highly exposed to biological dust according to ACE JEM, but only a few articles (eight studies) substantiate this. Also in the literature there was insufficient information to properly support the medium exposure assignments to fumes, diesel exhaust particulate and mineral dust. Moreover, the difference between the low exposed and the unexposed is unclear in our sample, given that there was lack of evidence for both categories. For example, there is little difference between the low exposed mineral dust category and the unexposed mineral dust, or it is not clear why the occupations determined to be exposed to low levels of fumes are different from the jobs identified as unexposed to fumes. Therefore, as mentioned above, the ACE JEM is only really sustained for high exposure, and to a lesser extent medium exposure, to dust (and biological dust) and these exposure assessments are supported by the published literature.

Kappa statistics (Table 7) indicate moderate inter-rater reliability between our estimation and JEM assigned exposures for almost all five exposures that we examined, except from exposure to mineral dust where the value of kappa suggests poor agreement [29]. This is generally consistent with the simple agreement percentages (number of agreement scores/total scores), which however ignore chance agreement between raters. However, most of the agreement appears to come from the fact that the two approaches generally agree on the unexposed level (primarily) and the high level of exposure, while agreement at medium and low exposure levels is unclear (Table 8, Figures S1 and S2). For proportions exposed (Table 7), the estimated values of the kappa statistic show poor inter-rater reliability between the ratings.

While the UK Biobank population is broadly representative of the UK population, it underrepresents people in lower socioeconomic groups. It is probable that many occupations where exposures are high, were performed by people in lower socioeconomic groups; this is an important limitation in using the UK Biobank data to investigate occupational exposure to airborne dusts. In this study, we limit our assessment to the jobs that are commonly found in the UK Biobank and it is probable that there is more published evidence to substantiate the ACE JEM for a wider range of occupations. Our study also only provides information on a small number of occupations; only 43 commonly encountered jobs were used to assess the JEM. Our conclusions from this sample are extrapolated to the whole JEM (353 occupations). Also, there is a possibility that some occupations that were exposed to dust, fumes and diesel might not have been investigated by researchers in the past, and while it could be reasonable to assume workers were exposed according to expert judgement, there might be insufficient literature to support the assignments, as this work focused only on the public literature, but there are a lot of unpublished datasets. Our results suggest that although the ACE JEM might be appropriate for the original purpose, it should be used with caution when investigating other diseases in relation to occupational exposures. The matrix could be applicable with some modifications, and it should be kept in mind that no matrix is a perfect gold standard due to exposure misclassification within the occupation group [30].

The objective of this paper was to examine the suitability of the ACE JEM for assessing exposure to airborne dust, fumes and diesel exhaust particulate, and to determine how it could best be applied to study other health outcomes for UK Biobank participants. We conclude that the ACE JEM assignments are probably reliable for highly exposed jobs and for jobs assigned as unexposed. However, the assignments for medium exposed and low exposed are less reliable. Therefore, we suggest three options to use the JEM in future analyses. Firstly, it could be used as it is and the results from the epidemiological analyses should be cautiously interpreted in the light of the reliability of the underlying exposure assessments. Secondly, the JEM could be improved or at least adapted to increase the reliability of the assessments. For adaptation, there are several paths that could be followed. One option would be to take high exposures and unexposed as they are by focusing on the two extremes, because of the agreement of the literature and the agreement between the assessors, and assume that they are reliable (Table 8). Then, all the other categories could be merged into one medium/low category. Alternatively, the highly and medium exposed categories could remain as they are and a merged category could be created that would include both the low exposed and unexposed, as there is poor evidence for both of these categories, or the high and medium categories could be merged together and the low and unexposed together, so that we would create two larger categories. These merged categories could be used to perform sensitivity analyses with slightly different groupings and see if they alter the outcome. The best of those three adaptations would probably be to merge the high with medium category and low with unexposed, as it seems that there is more agreement arising from this strategy. Thirdly, the reliability of the ACE JEM could be improved by sourcing more exposure data about levels of exposure, increasing the information contributing to the exposure assignment, for example by adding details of tasks undertaken, or getting a wider range of experts to assess the exposures and add more varied perspectives.

## 5. Conclusions

The ACE JEM might be a good instrument to examine occupational exposures that contribute to COPD development, as it has demonstrated positive findings in epidemiological analyses in the UK Biobank cohort, which strengthens its reliability. However, it should be improved, or used with caution, when used as a tool to examine associations between occupational exposures and other diseases, such as dementia or diabetes.

## Figures and Tables

**Table 1:** The most common occupations in UK Biobank and their level of exposure and the proportion exposed to dust according to ACE JEM.

Dust																	
Exposure															Unexposed		
High					Medium					Low							
Job Codes	Job	UK Biobank Participants (n)	ACE JEM (P) Exposed ^a^	ACE JEM Exposure (L) ^b^	Job Codes	Job	UK Biobank Participants (n)	ACE JEM (P) Exposed	ACE JEM Exposure (L)	Job Codes	Job	UK Biobank Participants (n)	ACE JEM (P) Exposed	ACE JEM Exposure (L)	Job Codes	Job	UK Biobank Participants (n)
9139	Labourers in process and plant operations n.e.c. ^c^	1303	3	3	5223	Metal working production and maintenance fitters	1589	3	2	3211	Nurses	6748	2	1	2314	Secondary education teaching professionals	9974
5315	Carpenters and joiners	767	3	3	5231	Motor mechanics, auto engineers	1024	3	2	3111	Laboratory technicians	3375	1	1	2315	Primary and nursery education teaching professionals	7302
9121	Labourers in building and woodworking trades	715	3	3	9233	Cleaners, domestics	910	3	2	3311	NCOs ^d^ and other ranks	2659	1	1	4215	Personal assistants and other secretaries	7220
8149	Construction operatives n.e.c.	504	3	3	9111	Farm workers	476	3	2	3119	Science and engineering technicians n.e.c.	2625	1	1	4113	Local government clerical officers and assistants	6374
5319	Construction trades n.e.c.	372	3	3	5323	Painters and decorators	475	3	2	2211	Medical practitioners	2118	2	1	7111	Sales and retail assistants	6225

^a^ Proportion exposed according to ACE JEM (<5% = 0, 5–19% = 1, 20–49% = 2, >50% = 3); ^b^ Exposure level according to ACE JEM (None = 0, Low = 1, Medium = 2, High = 3); ^c^ n.e.c.: not elsewhere classified; ^d^ NCOs: Non-commissioned officers.

**Table 2:** The most common occupations in UK Biobank and their level of exposure and the proportion exposed to fumes according to ACE JEM.

Fumes																	
Exposure															Unexposed		
High					Medium					Low							
Job Codes	Job	UK Biobank Participants (n)	ACE JEM (P) Exposed ^a^	ACE JEM Exposure (L) ^b^	Job Codes	Job	UK Biobank Participants (n)	ACE JEM (P) Exposed	ACE JEM Exposure (L)	Job Codes	Job	UK Biobank Participants (n)	ACE JEM (P) Exposed	ACE JEM Exposure (L)	Job Codes	Job	UK Biobank Participants (n)
5215	Welding trades	358	3	3	9139	Labourers in process and plant operations n.e.c.	1303	3	2	3311	NCOs and other ranks	2659	1	1	2314	Secondary education teaching professionals	9974
3313	Fire service officers (leading fire officer and below)	253	3	3	3112	Electrical/electronic technicians	1242	1	2	2122	Mechanical engineers	2067	2	1	2315	Primary and nursery education teaching professionals	7302
5213	Sheet metal workers	180	3	3	5434	Chefs, cooks	1127	3	2	8139	Assemblers and routine operatives n.e.c.	1665	2	1	4215	Personal assistants and other secretaries	7220
5212	Moulders, core makers, die casters	38	3	3	5231	Motor mechanics, auto engineers	1024	3	2	5223	Metal working production and maintenance fitters	1589	2	1	3211	Nurses	6748
5211	Smiths and forge workers	37	3	3	9121	Labourers in building and woodworking trades	715	3	2	3312	Police officers (sergeant and below)	1488	3	1	4113	Local government clerical officers and assistants	6374

^a^ Proportion exposed according to ACE JEM (<5% = 0, 5–19% = 1, 20–49% = 2, >50% = 3); ^b^ Exposure level according to ACE JEM (None = 0, Low = 1, Medium = 2, High = 3).

**Table 3:** The most common occupations in UK Biobank and their level of exposure and the proportion exposed to diesel according to ACE JEM.

Diesel																	
Exposure															Unexposed		
High					Medium					Low							
Job Codes	Job	UK Biobank Participants (n)	ACE JEM (P) Exposed ^a^	ACE JEM Exposure (L) ^b^	Job Codes	Job	UK Biobank Participants (n)	ACE JEM (P) Exposed	ACE JEM Exposure (L)	Job Codes	Job	UK Biobank Participants (n)	ACE JEM (P) Exposed	ACE JEM Exposure (L)	Job Codes	Job	UK Biobank Participants (n)
-	-	-	-	-	5231	Motor mechanics, auto engineers	1024	3	2	3311	NCOs and other ranks	2659	1	1	2314	Secondary education teaching professionals	9974
-	-	-	-	-	3513	Ship and hovercraft officers	445	1	2	2122	Mechanical engineers	2067	2	1	2315	Primary and nursery education teaching professionals	7302
-	-	-	-	-	8122	Coal mine operatives	348	3	2	3312	Police officers (sergeant and below)	1488	3	1	4215	Personal assistants and other secretaries	7220
-	-	-	-	-	1232	Garage managers and proprietors	283	3	2	3542	Sales representatives	1475	1	1	3211	Nurses	6748
-	-	-	-	-	3313	Fire service officers (leading fire officer and below)	253	3	2	1122	Managers in construction	1355	1	1	4113	Local government clerical officers and assistants	6374

^a^ Proportion exposed according to ACE JEM (<5% = 0, 5–19% = 1, 20–49% = 2, >50% = 3); ^b^ Exposure level according to ACE JEM (None = 0, Low = 1, Medium = 2, High = 3).

**Table 4:** The most common occupations in UK Biobank and their level of exposure and the proportion exposed to mineral dust according to ACE JEM.

Mineral Dust																	
Exposure															Unexposed		
High					Medium					Low							
Job Codes	Job	UK Biobank Participants (n)	ACE JEM (P) Exposed ^a^	ACE JEM Exposure (L) ^b^	Job Codes	Job	UK Biobank Participants (n)	ACE JEM (P) Exposed	ACE JEM Exposure (L)	Job Codes	Job	UK Biobank Participants (n)	ACE JEM (P) Exposed	ACE JEM Exposure (L)	Job Codes	Job	UK Biobank Participants (n)
9139	Labourers in process and plant operations n.e.c. ^c^	1303	3	3	5223	Metal working production and maintenance fitters	1589	3		3111	Laboratory technicians	3375	1		2314	Secondary education teaching professionals	9974
9121	Labourers in building and woodworking trades	715	3	3	5231	Motor mechanics, auto engineers	1024	3		3311	NCOs and other ranks	2659	1		2315	Primary and nursery education teaching professionals	7302
8149	Construction operatives n.e.c.	504	3	3	9233	Cleaners, domestics	910	3		3119	Science and engineering technicians n.e.c.	2625	1		4215	Personal assistants and other secretaries	7220
8113	Textile process operatives	352	2	3	9111	Farm workers	476	3		2122	Mechanical engineers	2067	1		3211	Nurses	6748
8122	Coal mine operatives	348	3	3	5323	Painters and decorators	475	3		8139	Assemblers and routine operatives n.e.c.	1665	3		4113	Local government clerical officers and assistants	6374

^a^ Proportion exposed according to ACE JEM (<5% = 0, 5–19% = 1, 20–49% = 2, >50% = 3); ^b^ Exposure level according to ACE JEM (None = 0, Low = 1, Medium = 2, High = 3); ^c^ n.e.c.: not elsewhere classified; NCOs: Non-commissioned officers.

**Table 5:** The most common occupations in UK Biobank and their level of exposure and the proportion exposed to biological dust according to ACE JEM.

Biological Dust																	
Exposure														Unexposed			
High					Medium					Low							
Job codes	Job	UK Biobank Participants (n)	ACE JEM (P) Exposed ^a^	ACE JEM Exposure (L) ^b^	Job Codes	Job	UK Biobank Participants (n)	ACE JEM (P) Exposed	ACE JEM Exposure (L)	Job Codes	Job	UK Biobank Participants (n)	ACE JEM (P) Exposed	ACE JEM Exposure (L)	Job Codes	Job	UK Biobank Participants (n)
5315	Carpenters and joiners	767	3	3	9121	Labourers in building and woodworking trades	715	3	2	3211	Nurses	6748	2	1	2314	Secondary education teaching professionals	9974
8113	Textile process operatives	352	3	3	9111	Farm workers	476	3	2	3111	Laboratory technicians	3375	1	1	2315	Primary and nursery education teaching professionals	7302
8111	Food, drink and tobacco process operatives	315	3	3	5111	Farmers	236	3	2	2211	Medical practitioners	2118	2	1	4215	Personal assistants and other secretaries	7220
5432	Bakers, flour confectioners	250	3	3	8134	Weighers, graders, sorters	224	3	2	6111	Nursing auxiliaries and assistants	1730	2	1	4113	Local government clerical officers and assistants	6374
5492	Furniture makers, other craft woodworkers	250	3	3	8121	Paper and wood machine operatives	212	3	2	6115	Care assistants and home carers	1686	1	1	7111	Sales and retail assistants	6225

^a^ Proportion exposed according to ACE JEM (<5% = 0, 5–19% = 1, 20–49% = 2, >50% = 3); ^b^ Exposure level according to ACE JEM (None = 0, Low = 1, Medium = 2, High = 3).

**Table 6:** Information gathered from the literature search for the most common occupations in UK Biobank for all the 5 agents.

High		Medium		Low		Unexposed	
Job	Number of Papers Included	Job	Number of Papers Included	Job	Number of Papers Included	Job	Number of Papers Included
**Dust**							
Labourers in process and plant operations n.e.c.	1	Metal working production and maintenance fitters	12	Nurses	7	Secondary education teaching professionals	-
Carpenters and joiners	37	Motor mechanics, auto engineers	5	Laboratory technicians	8	Primary and nursery education teaching professionals	-
Labourers in building and woodworking trades	6	Cleaners, domestics	3	NCOs and other ranks	2	Personal assistants and other secretaries	1
Construction operatives n.e.c.	73	Farm workers	19	Science and engineering technicians n.e.c.	-	Local government clerical officers and assistants	-
Construction trades n.e.c	5	Painters and decorators	7	Medical practitioners	-	Sales and retail assistants	-
**Fumes**							
Welding trades	3	Labourers in process and plant operations n.e.c.	-	NCOs and other ranks	3	Secondary education teaching professionals	-
Fire service officers (leading fire officer and below)	4	Electrical/electronics technicians	1	Mechanical engineers	-	Primary and nursery education teaching professionals	-
Sheet metal workers	2	Chefs, cooks	12	Assemblers and routine operatives n.e.c.	2	Personal assistants and other secretaries	-
Moulders, core makers, die casters	1	Motor mechanics, auto engineers	-	Metal working production + maintenance fitters	-	Nurses	-
Smiths and forge workers	2	Labourers in building and woodworking trades	1	Police officers (sergeant and below)	1	Local government clerical officers and assistants	-
**Diesel**							
-	-	Motor mechanics, auto engineers	11	NCOs and other ranks	-	Secondary education teaching professionals	-
-	-	Ship and hovercraft officers	-	Mechanical engineers	2	Primary and nursery education teaching professionals	-
-	-	Coal mine operatives	21	Police officers (sergeant and below)	-	Personal assistants and other secretaries	-
-	-	Garage managers and proprietors	5 papers about garage WORKERS!	Sales representatives	-	Nurses	-
-	-	Fire service officers (leading fire officer and below)	1	Managers in construction	5 papers about construction WORKERS!	Local government clerical officers and assistants	-
**Biological Dust**							
Carpenters and joiners	29	Labourers in building and woodworking trades	-	Nurses	-	Secondary education teaching professionals	-
Textile process operatives	8	Farm workers	10	Laboratory technicians	-	Primary and nursery education teaching professionals	-
Food, drink and tobacco process operatives	-	Farmers	53	Medical practitioners	-	Personal assistants and other secretaries	-
Bakers, flour confectioners	36	Weighers, graders, sorters	-	Nursing auxiliaries and assistants	-	Local government clerical officers and assistants	-
Furniture makers, other craft woodworkers	40	Paper and wood machine operatives	-	Care assistants and home carers	-	Sales and retail assistants	-
**Mineral Dust**							
Labourers in process and plant operations n.e.c.	-	Metal working production and maintenance fitters	2	Laboratory technicians	1	Secondary education teaching professionals	-
Labourers in building and woodworking trades	-	Motor mechanics, auto engineers	-	NCOs and other ranks	1	Primary and nursery education teaching professionals	-
Construction operatives n.e.c.	15	Cleaners, domestics	2	Science and engineering technicians n.e.c.	-	Personal assistants and other secretaries	-
Textile process operatives	-	Farm workers	2	Mechanical engineers	-	Nurses	-
Coal mine operatives	1	Painters and decorators	2	Assemblers and routine operatives n.e.c.	1	Local government clericalofficers and assistants	-

n.e.c.: not elsewhere classified; NCOs: Non-commissioned officers.

**Table 7:** Agreement between assessors and Cohen’s kappa for level of exposure and proportions exposed.

	Dust	Fumes	Diesel	Mineral Dust	Biological Dust
Level of exposure					
Agreement (%)	58	67	67	51	74
Cohen’s kappa	0.44	0.52	0.42	0.22	0.58
Proportions exposed					
Agreement (%)	56	56	67	56	65
Cohen’s kappa	0.39	0.30	0.40	0.28	0.40

**Table 8:** Classification from the two assessments for level of exposure and proportions exposed.

JWC ^a^																		
**ACE JEM**	**Level**		**High**				**Medium**				**Low**				**Non-exposed**			
		**Proportion**	**3**	**2**	**1**	**0**	**3**	**2**	**1**	**0**	**3**	**2**	**1**	**0**	**3**	**2**	**1**	**0**
	High	**3**	14	1	1		4	1		1	1		1					
		**2**																1
		**1**																
		**0**																
	Medium	**3**	2	4	2		1	5	8	1	1		2	1				7
		**2**																
		**1**	1						1									
		**0**							1									
	Low	**3**		1					1	1								2
		**2**			1			1	5	1	2		4	1				3
		**1**							4				6	2			1	6
		**0**											1					
	Non-exposed	**3**	1				1	1										1
		**2**																
		**1**																
		**0**		1	3				3	2	1		9	3	2			82

^a^ Independent assessor John W. Cherrie.

## Supplementary Materials

The following are available online at https://www.mdpi.com/1660-4601/17/14/4919/s1, Detailed search terms and combinations that were used while searching in PubMed and Scopus databases related to the occupation and the type of exposure of interest. Tables S1–S5: References related to the occupation and the type of exposure of interest. Tables S6–S10: Detailed tables about number of studies used in each category (exposure studies, epidemiological studies and other). Table S11: Exposure levels and proportions exposed according to ACE JEM and independent exposure assessor. Figures S1 and S2: Graphical representation of agreement (percentages) between assessors for level of exposure and proportion exposed, respectively. References corresponding to Tables S1–S5.
