# Supplementary material for: Evaluation of the Suitability of an Existing Job–Exposure Matrix for the Assessment of Exposure of UK Biobank Participants to Dust, Fumes, and Diesel Exhaust Particulates
Source: Int J Environ Res Public Health. 2020 Jul 8;17(14):4919. doi: 10.3390/ijerph17144919 (PMC7400423; doi:10.3390/ijerph17144919)
Supplement: Supplementary file 1 [file ijerph-17-04919-s001.zip › Supplementary_material.docx]

Supplementary material

**Evaluation of the suitability of an existing job-exposure matrix for assessment of exposure of UK-Biobank participants to dust, fumes, and diesel exhaust particulates**

Eirini Dimakakou^1^, Helinor J Johnston^1^, George Streftaris^2^, John W Cherrie^1,3^

^1^ School of Engineering and Physical Sciences, Institute of Biological Chemistry, Biophysics and Bioengineering, Heriot-Watt University, Riccarton, Edinburgh EH14 4AS, UK

^2^ Maxwell Institute for Mathematical Sciences, School of Mathematical and Computer Sciences, Heriot-Watt University, Edinburgh EH14 4AS, UK

^3^ Institute of Occupational Medicine (IOM), Riccarton, Edinburgh EH14 4AP, UK

| **Table of Contents** | |
| --- | --- |
| 1. Literature search terms | pg. 2 |
| 2. Tables | pg. 9 |
| 3. Figures | pg. 50 |
| 4. References | Pg. 52 |

**1. Literature search terms**

PubMed and Scopus databases were used for our literature search. For each occupation and for each of the two databases different search terms were used.

For dust exposure:

for the “labourers in process and plant operations n.e.c.” we have used ("labourers" AND ("process" OR "plant operation*")) AND (dust OR "particulate matter") and ("labourers in process" OR "labourers in plant operation") AND (dust OR "particulate matter") for PubMed and the same for Scopus, although this dataset searches this search term at the end: TITLE-ABS-KEY ((“labourers" AND (“process" OR "plant operation*”)) AND ( dust OR "particulate matter”)).

For the “carpenters and joiners” we have used ("carpenters" OR "joiners") AND (dust OR "particulate matter") search terms for PubMed and Scopus.

For the “labourers in building and woodworking trades” we used ("labourers" AND ("building" OR "woodworking trade*")) AND (dust OR "particulate matter") for both datasets.

For “construction operatives n.e.c.” we searched ("construction operatives")) AND (dust OR "particulate matter") and then ("construction") AND (dust OR "particulate matter"), as in the first search no results were found.

For “construction trades n.e.c.” we used ("construction trades") AND (dust OR "particulate matter") search term.

For “metal working production and maintenance fitters” we used ("metal working production" OR "maintenance fitters”) AND (dust OR "particulate matter") search term.

For “motor mechanics, auto engineers” we searched by typing ("motor mechanics" OR "auto engineers”) AND (dust OR "particulate matter").

For “cleaners and domestics” we used the following search term: ("cleaners" OR "domestics”) AND (dust OR "particulate matter").

For “farm workers” and for “painters and decorators” we used the following terms accordingly: ("farm workers”) AND (dust OR "particulate matter") and ("painters" OR "decorators”) AND (dust OR "particulate matter").

For “nurses” we searched as follows: ("nurses”) AND (dust OR "particulate matter").

For “laboratory technicians”, ("laboratory technicians”) AND (dust OR "particulate matter").

For “NCOs and other ranks” we used the following search term: ("NCO" OR "non-commissioned officer" OR "military personnel" OR "soldier*" OR "airmen" OR "sailor*") AND (dust OR "particulate matter").

For “science and engineering technicians n.e.c” we typed ("science technicians" OR "engineering technicians") AND (dust OR "particulate matter") and for “medical practitioners”, ("medical practitioners") AND (dust OR "particulate matter").

For “secondary education teaching professionals”, ”primary and nursery education teaching professionals”, ”personal assistants and other secretaries”, ”local government clerical officers and assistants” and “sales and retail assistants” we searched the following accordingly: ("secondary education teaching professionals") AND (dust OR "particulate matter"), ("primary and nursery education teaching professionals") AND (dust OR "particulate matter"), ("personal assistants" OR "secretaries") AND (dust OR "particulate matter"), ("local government clerical officer*" OR "local government clerical assistants") AND (dust OR "particulate matter") and ("sales assistants" OR "retail assistants") AND (dust OR "particulate matter").

For fumes exposure:

For “welding trades” we search by using this combination: ("welding trades" AND fumes) and for “fire service officers”, (("fire service officers" OR "firemen") AND fumes) for PubMed and TITLE-ABS-KEY ((“firemen" OR "fire service officers”) AND "fumes”) AND ( LIMIT-TO ( LANGUAGE ,”English”)) for Scopus.

For “Sheet metal workers” we searched (“Sheet metal workers” AND fumes) in PubMed and

TITLE-ABS-KEY ("sheet metal workers" AND fumes) AND (LIMIT-TO (LANGUAGE,” English")) in Scopus.

For “Moulders, core makers, die casters” we used the (("moulders" OR "core makers" OR "die casters") AND fumes ) search term in PubMed and the TITLE-ABS-KEY ((“moulders" OR "core makers" OR "die casters" AND fumes )) AND ( LIMIT-TO ( LANGUAGE ,”English”)) search term in Scopus.

For “Smiths and forge workers”, ("smiths" OR "forge workers" AND fumes) and

TITLE-ABS-KEY (("smiths" OR "forge workers" AND fumes)) AND (LIMIT-TO (LANGUAGE,” English")).

For “Labourers in process and plant operations n.e.c.” we searched in PubMed and Scopus, ("labourers” AND (“process” OR “plant operation*”)) AND fumes) and

TITLE-ABS-KEY (("labourers" AND ("process" OR "plant operation*")) AND fumes) accordingly.

For “Electrical/ electronic technicians” we searched in PubMed ("technicians" AND ("electrical" OR "electronic*")) AND fumes) and in Scopus TITLE-ABS-KEY (("technicians" AND ("electrical" OR "electronic*")) AND fumes).

For “Chefs, cooks” we searched in PubMed (("chef*” OR “cook*”) AND fumes) and in Scopus

TITLE-ABS-KEY ((“chef*" OR "cook*”) AND fumes ) AND ( LIMIT-TO ( LANGUAGE ,”English”)) AND ( LIMIT-TO ( AFFILCOUNTRY ,”United States”) OR LIMIT-TO ( AFFILCOUNTRY ,”Norway”) OR LIMIT-TO ( AFFILCOUNTRY ,”United Kingdom”) OR LIMIT-TO ( AFFILCOUNTRY ,”Canada”) OR LIMIT-TO ( AFFILCOUNTRY ,”Sweden”) OR LIMIT-TO ( AFFILCOUNTRY ,”Australia”) OR LIMIT-TO ( AFFILCOUNTRY ,”Italy”) OR LIMIT-TO ( AFFILCOUNTRY ,”Spain”) OR LIMIT-TO ( AFFILCOUNTRY ,”Finland”) OR LIMIT-TO ( AFFILCOUNTRY ,”France”) OR LIMIT-TO ( AFFILCOUNTRY ,”Turkey”) OR LIMIT-TO ( AFFILCOUNTRY ,”Germany”) OR LIMIT-TO ( AFFILCOUNTRY ,”Switzerland”) OR LIMIT-TO ( AFFILCOUNTRY ,”Netherlands”) OR LIMIT-TO ( AFFILCOUNTRY ,”Portugal”) OR LIMIT-TO ( AFFILCOUNTRY ,”Belgium”) OR LIMIT-TO ( AFFILCOUNTRY ,”Denmark”) OR LIMIT-TO ( AFFILCOUNTRY ,”Greece”) OR LIMIT-TO ( AFFILCOUNTRY ,”New Zealand”) OR LIMIT-TO ( AFFILCOUNTRY ,”Poland”) OR LIMIT-TO ( AFFILCOUNTRY ,”Romania”)) in order to narrow down the results.

For “Motor mechanics, auto engineers” we searched (("motor” OR “auto" AND "mechanic*" OR "engineer*”) AND fumes) in both datasets.

For “Labourers in building and woodworking trades” we used the following search term for both datasets: (((“labourers" AND (“building”)) OR "woodworking trades”) AND fumes).

For “NCOs and other ranks” (("NCOs" OR "non-commissioned officer" OR "soldier*" OR "airmen" OR "sailors") AND fumes) and for “Mechanical engineers” ("mechanical engineers" AND fumes), for “Assemblers and routine operatives n.e.c.” ((“Assembler*" OR "routine operatives”) AND fumes ) and for “Metal working production and maintenance fitters” ((“metal working production" OR "maintenance fitters”) AND fumes ) and for “Police officers (sergeant and below)” ((“police officers”) AND fumes ).

For diesel exposure:

For “motor mechanics, auto engineers” we used (("motor mechanics" OR "auto engineers" OR "mechanics") AND ("diesel" OR "diesel exhaust")) search term for the PubMed search and TITLE-ABS-KEY (((“motor" OR "auto" AND "mechanic*" OR "engineer*”) AND diesel )) AND ( LIMIT-TO ( LANGUAGE ,”English”)) and more specifically: TITLE-ABS-KEY (((“motor" OR "auto”) AND (“mechanic*" OR "engineer*”)) AND diesel ) AND ( LIMIT-TO ( LANGUAGE ,”English”)) AND ( LIMIT-TO ( AFFILCOUNTRY ,”United States”) OR LIMIT-TO ( AFFILCOUNTRY ,”Germany”) OR LIMIT-TO ( AFFILCOUNTRY ,”United Kingdom”) OR LIMIT-TO ( AFFILCOUNTRY ,”Canada”) OR LIMIT-TO ( AFFILCOUNTRY ,”Sweden”) OR LIMIT-TO ( AFFILCOUNTRY ,”Italy”) OR LIMIT-TO ( AFFILCOUNTRY ,”Finland”) OR LIMIT-TO ( AFFILCOUNTRY ,”Norway”) OR LIMIT-TO ( AFFILCOUNTRY ,”Austria”) OR LIMIT-TO ( AFFILCOUNTRY ,”Netherlands”) OR LIMIT-TO ( AFFILCOUNTRY ,”Poland”) OR LIMIT-TO ( AFFILCOUNTRY ,”Portugal”) OR LIMIT-TO ( AFFILCOUNTRY ,”Switzerland”) OR LIMIT-TO ( AFFILCOUNTRY ,”Belgium”) OR LIMIT-TO ( AFFILCOUNTRY ,”Denmark”) OR LIMIT-TO ( AFFILCOUNTRY ,”New Zealand”)) in order to limit our search.

For “ship and hovercraft officers” the search term was (("ship officers" OR "hovercraft officers") AND ("diesel" OR "diesel exhaust")) for both datasets.

For “coal mine operatives” we used ("coal mine workers" OR "coal mine") AND ("diesel" OR "diesel exhaust") for PubMed and TITLE-ABS-KEY ((“coal mine workers" OR "coal mine”) AND (“diesel" OR "diesel exhaust”)) AND ( LIMIT-TO ( LANGUAGE ,”English”)) AND ( LIMIT-TO ( AFFILCOUNTRY ,”United States”) OR LIMIT-TO ( AFFILCOUNTRY ,”Australia”) OR LIMIT-TO ( AFFILCOUNTRY ,”Germany”) OR LIMIT-TO ( AFFILCOUNTRY ,”United Kingdom”) OR LIMIT-TO ( AFFILCOUNTRY ,”Canada”) OR LIMIT-TO ( AFFILCOUNTRY ,”Netherlands”) OR LIMIT-TO ( AFFILCOUNTRY ,”Austria”) OR LIMIT-TO ( AFFILCOUNTRY ,”France”) OR LIMIT-TO ( AFFILCOUNTRY ,”Norway”) OR LIMIT-TO ( AFFILCOUNTRY ,”Russian Federation”) OR LIMIT-TO ( AFFILCOUNTRY ,”Denmark”) OR LIMIT-TO ( AFFILCOUNTRY ,”Greece”) OR LIMIT-TO ( AFFILCOUNTRY ,”Switzerland”)), to narrow down the search in Scopus.

For “garage managers and proprietors” we used ("garage managers" OR "garage proprietors") AND ("diesel" OR "diesel exhaust" OR "diesel engine exhaust") and

("garage") AND ("diesel" OR "diesel exhaust" OR "diesel engine exhaust") for PubMed and TITLE-ABS-KEY ((“garage" AND "managers" OR "proprietors”) AND (“diesel" OR "diesel exhaust" OR "diesel engine exhaust”)) for Scopus.

For “fire service officers” we searched in both datasets: (("firemen" OR "fire service officers") AND ("diesel" OR "diesel exhaust" OR "diesel engine exhaust")).

For “NCOs and other ranks”: (("NCOs" OR "non-commissioned officer" OR "soldier*" OR "airmen" OR "sailors") AND ("diesel" OR "diesel exhaust" OR "diesel engine exhaust")).

For “mechanical engineers”: (("mechanical engineers") AND ("diesel exposure" OR "diesel exhaust" OR "diesel engine exhaust")).

For “police officers”: (("police officers") AND ("diesel exposure" OR "diesel exhaust" OR "diesel engine exhaust" OR "black carbon")).

For “sales representatives”: (("sales representatives ") AND ("diesel exposure" OR "diesel exhaust" OR "diesel engine exhaust")).

For “managers in construction”: (("managers" AND "construction") AND ("diesel exposure" OR "diesel exhaust" OR "diesel engine exhaust" OR "black carbon")).

For “secondary education teaching professionals”, ”primary and nursery education teaching professionals”, ”personal assistants and other secretaries”, ”local government clerical officers and assistants” and “nurses” we searched the following accordingly: ("secondary education teaching professionals") AND ("diesel" OR "diesel exhaust" OR "diesel engine exhaust")), ("primary and nursery education teaching professionals") AND ("diesel" OR "diesel exhaust" OR "diesel engine exhaust")), ("personal assistants" OR "secretaries") AND ("diesel" OR "diesel exhaust" OR "diesel engine exhaust")), ("local government clerical officer*" OR "local government clerical assistants") AND ("diesel" OR "diesel exhaust" OR "diesel engine exhaust")) and ("nurses" AND ("diesel" OR "diesel exhaust" OR "diesel engine exhaust")).

For biological dust:

For “carpenters and joiners” we searched: (("carpenters" OR "joiners") AND ("wood dust" OR "bio dust" OR "biological dust")) in both databases. Also, we added here the papers found while searching dust, as wood dust is a biological dust.

For “textile process operatives” we used ("Textile process operatives ") AND ("bio-dust" OR "biological dust" OR "bio dust" OR "dust") for PubMed and TITLE-ABS-KEY ((“textile process operatives”) AND (“wood dust" OR "bio dust" OR "biological dust”)) and TITLE-ABS-KEY ((“textile workers”) AND (“wood dust" OR "bio dust" OR "biological dust" OR "organic dust”)) in Scopus.

For “food, drink and tobacco process operatives” we used the following search term: ((“food workers" OR "drink process operator" OR "tobacco workers”) AND (“wood dust" OR "bio dust" OR "biological dust" OR "organic dust”)).

For “bakers, flour confectioners” ("bakers" OR "flour confectioners") AND dust in PubMed

and TITLE-ABS-KEY ((“bakers" OR "flour confectioners”) AND (“wood dust" OR "bio dust" OR "biological dust" OR "organic dust”)) AND (LIMIT-TO (LANGUAGE,” English”)) in Scopus.

For “furniture makers, other craft woodworkers”, ("Furniture makers" OR "craft woodworkers" OR "woodworkers") AND ("bio-dust" OR "biological dust" OR "bio dust" OR "organic dust") and then we added OR “dust” to broad our category and detect more papers. For the Scopus search we typed (("furniture makers" OR "craft woodworkers" OR "woodworkers”) AND (“wood dust" OR "bio dust" OR "biological dust" OR "organic dust”)).

For “labourers in building and woodworking trades” we searched in PubMed ("Labourers in building" OR "Labourers woodworking trades" OR "woodworking trades") AND ("wood dust" OR "bio-dust" OR "biological dust" OR "bio dust" OR "organic dust") and in Scopus TITLE-ABS-KEY ((“labourers" AND (“building" OR "woodworking trades”)) AND (“wood dust" OR "bio dust" OR "biological dust" OR "organic dust”)).

For “farm workers”: (("farm workers") AND ("wood dust" OR "bio dust" OR "biological dust" OR "organic dust")).

For “weighers, graders, sorters” and “paper and wood machine operatives” we typed ((“weighers” OR “graders” OR “sorters”) AND ("wood dust" OR "bio-dust" OR "biological dust" OR "bio dust" OR "organic dust")) and (("paper machine operatives" OR "wood machine operatives") AND ("wood dust" OR "bio-dust" OR "biological dust" OR "bio dust" OR "organic dust")) accordingly.

For “farmers” (("farmers") AND ("bio dust" OR "biological dust" OR "organic dust")).

For “laboratory technicians” (("Laboratory technicians") AND ("wood dust" OR "bio-dust" OR "biological dust" OR "bio dust" OR "organic dust")), for “medical practitioners” (("Medical practitioners") AND ("wood dust" OR "bio-dust" OR "biological dust" OR "bio dust" OR "organic dust"), for “nursing auxiliaries and assistants”, (("Nursing auxiliaries" OR "nursing assistants") AND ("wood dust" OR "bio-dust" OR "biological dust" OR "bio dust" OR "organic dust")) and for “care assistants and home carers”, (("Care assistants" OR "home carers") AND ("wood dust" OR "bio-dust" OR "biological dust" OR "bio dust" OR "organic dust")).

For all the unexposed occupations for bio-dust we did the exact search as in the unexposed category of dust, but instead of only “dust” we searched also ("wood dust" OR "bio-dust" OR "biological dust" OR "bio dust" OR "organic dust").

For mineral dust exposure:

For “labourers in process and plant operations n.e.c.”, (("Labourers in process" OR "Labourers in plant operations") AND ("min-dust" OR "mineral dust" OR "min dust")) and also ((“labourers" AND (“process" OR "plant operation*”)) AND (“min-dust" OR "mineral dust" OR "min dust”)).

For “labourers in building and woodworking trades”, (("Labourers in building" OR "Labourers in woodworking trades") AND ("min-dust" OR "mineral dust" OR "min dust") and also ((“labourers" AND (“building" OR "woodworking trades”)) AND (“min-dust" OR "mineral dust" OR "min dust”)).

For “construction operatives n.e.c.”, (("Construction workers") AND ("min-dust" OR "mineral dust" OR "min dust")) and ((“construction" AND (“workers" OR "operatives”)) AND (“min-dust" OR "mineral dust" OR "min dust”)).

For “textile process operatives” we used the following search terms: (("Textile process operatives" OR "Textile process workers") AND ("min-dust" OR "mineral dust" OR "min dust")) and ((“Textile process operatives" OR "Textile process workers”) AND (“min-dust" OR "mineral dust" OR "min dust”)) and finally ((“Textile" AND (“process operatives" OR "process workers”)) AND (“min-dust" OR "mineral dust" OR "min dust”)).

For “coal mine operatives”, (("Coal mine operatives" OR "Coal mine workers") AND ("min-dust" OR "mineral dust" OR "min dust")).

For “metal working production and maintenance fitters”, (("metal working production" OR "maintenance fitters") AND ("quartz" OR "min-dust" OR "mineral dust" OR "min dust")) and also ((“metal production" OR "maintenance fitters”) AND (“quartz" OR "min-dust" OR "mineral dust" OR "min dust”)).

For “motor mechanics, auto engineers”, (("Motor mechanics" OR "auto engineers") AND ("min-dust" OR "mineral dust" OR "min dust")) and also (((“auto" OR "motor”) AND (“mechanics" OR "engineers”)) AND (“quartz exposure" OR "min-dust" OR "mineral dust" OR "min dust”)).

For “cleaners, domestics”, ((“cleaners” OR “domestics”) AND (“quartz exposure" OR "min-dust" OR "mineral dust" OR "min dust”)).

For “farm workers”, ("farm workers” AND (“quartz exposure” OR “min-dust” OR "mineral dust" OR "min dust”).

For “painters and decorators”, ((“painters" OR "decorators”) AND (“quartz exposure" OR "min-dust" OR "mineral dust" OR "min dust”)).

For “laboratory technicians”, ((“laboratory technicians”) AND (“quartz exposure" OR "min-dust" OR "mineral dust" OR "min dust”)) and for “NCOs and other ranks”, ((“non-commissioned officers" OR "soldiers" OR "airman" OR "military personnel" OR "sailor”) AND (“quartz exposure" OR "min-dust" OR "mineral dust" OR "min dust”)).

For “science and engineering technicians n.e.c.”, (("Science technicians" OR "engineering technicians") AND ("min-dust" OR "mineral dust" OR "min dust")) and ((“science technicians" OR "engineering technicians”) AND (“quartz exposure" OR "min-dust" OR "mineral dust" OR "min dust”)).

For “mechanical engineers”, ((“mechanical engineers”) AND (“quartz exposure" OR "min-dust" OR "mineral dust" OR "min dust”)) and for “assemblers and routine operatives n.e.c.”, (("Assemblers" OR "routine operatives") AND ("min-dust" OR "mineral dust" OR "min dust")) and ((“assemblers" OR "routine operatives”) AND (“quartz exposure" OR "min-dust" OR "mineral dust" OR "min dust”)).

For the unexposed group we did an identical search with diesel, but instead we put “AND (“quartz exposure" OR "min-dust" OR "mineral dust" OR "min dust”)” at the second part of our search term.

**2. Tables.**

**Tables with references related to the occupation and the type of exposure of interest (Tables S1a- S5c)**

**Table S1a**

| **Dust** | |
| --- | --- |
| **High** | |
| Labourers in process and plant operations n.e.c. | [1] |
| Carpenters and joiners | [2-38] |
| Labourers in building and woodworking trades | [39-44] |
| Construction operatives n.e.c. | [31,43,45-115] |
| Construction trades n.e.c | [98,116-119] |

**Table S1b**

| **Dust** | |
| --- | --- |
| **Medium** | |
| Metal working production and maintenance fitters | [120-131] |
| Motor mechanics, auto engineers | [132-136] |
| Cleaners, domestics | [137-139] |
| Farm workers | [140-158] |
| Painters and decorators | [159-165] |

**Table S1c**

| **Dust** | |
| --- | --- |
| **Low** | |
| Nurses | [166-172] |
| Laboratory technicians | [173-180] |
| NCOs and other ranks | [181,182] |
| Science and engineering technicians n.e.c. | - |
| Medical practitioners | - |

**Table S1d**

| **Dust** | |
| --- | --- |
| **Unexposed** | |
| Secondary education teaching professionals | - |
| Primary and nursery education teaching professionals | - |
| Personal assistants and other secretaries | [183] |
| Local government clerical officers and assistants | - |
| Sales and retail assistants | - |

**Table S2a**

| **Fumes** | |
| --- | --- |
| **High** | |
| Welding trades | [184-186] |
| Fire service officers (leading fire officer and below) | [187-190] |
| Sheet metal workers | [191,192] |
| Moulders, core makers, die casters | [193] |
| Smiths and forge workers | [194,195] |

**Table S2b**

| **Fumes** | |
| --- | --- |
| **Medium** | |
| Labourers in process and plant operations n.e.c. | - |
| Electrical/electronics technicians | [196] |
| Chefs, cooks | [197] [198-208] |
| Motor mechanics, auto engineers | - |
| Labourers in building and woodworking trades | [44] |

**Table S2c**

| **Fumes** | |
| --- | --- |
| **Low** | |
| NCOs and other ranks | [209-211] |
| Mechanical engineers | - |
| Assemblers and routine operatives n.e.c. | [196,212] |
| Metal working production + maintenance fitters | - |
| Police officers (sergeant and below) | [213] |

**Table S2d**

| **Fumes** | |
| --- | --- |
| **Unexposed** | |
| Secondary education teaching professionals | - |
| Primary and nursery education teaching professionals | - |
| Personal assistants and other secretaries | - |
| Nurses | - |
| Local government clerical officers and assistants | - |

**Table S3a**

| **Diesel** | |
| --- | --- |
| **High** | |
| - | - |
| - | - |
| - | - |
| - | - |
| - | - |

**Table S3b**

| **Diesel** | |
| --- | --- |
| **Medium** | |
| Motor mechanics, auto engineers | [214-224] |
| Ship and hovercraft officers | - |
| Coal mine operatives | [225-245] |
| Garage managers and proprietors | - |
| Fire service officers (leading fire officer and below) | [246] |

**Table S3c**

| **Diesel** | |
| --- | --- |
| **Low** | |
| NCOs and other ranks | - |
| Mechanical engineers | [247,248] |
| Police officers (sergeant and below) | - |
| Sales representatives | - |
| Managers in construction | [249] |

**Table S3d**

| **Diesel** | |
| --- | --- |
| **Unexposed** | |
| Secondary education teaching professionals | - |
| Primary and nursery education teaching professionals | - |
| Personal assistants and other secretaries | - |
| Nurses | - |
| Local government clerical officers and assistants | - |

**Table S4a**

| **Bio-Dust** | |
| --- | --- |
| **High** | |
| Carpenters and joiners | [2,4-12,14,15,18-20,23,25-30,32,34,35,38,250-252] |
| Textile process operatives | [253-260] |
| Food, drink and tobacco process operatives | - |
| Bakers, flour confectioners | [261-296] |
| Furniture makers, other craft woodworkers | [38,133,252,297-333] |

**Table S4b**

| **Bio-Dust** | |
| --- | --- |
| **Medium** | |
| Labourers in building and woodworking trades | - |
| Farm workers | [146,334-342] |
| Farmers | [144,343-394] |
| Weighers, graders, sorters | - |
| Paper and wood machine operatives | - |

**Table S4c**

| **Bio-Dust** | |
| --- | --- |
| **Low** | |
| Nurses | - |
| Laboratory technicians | - |
| Medical practitioners | - |
| Nursing auxiliaries and assistants | - |
| Care assistants and home carers | - |

**Table S4d**

| **Bio-Dust** | |
| --- | --- |
| **Unexposed** | |
| Secondary education teaching professionals | - |
| Primary and nursery education teaching professionals | - |
| Personal assistants and other secretaries | - |
| Local government clerical  officers and assistants | - |
| Sales and retail assistants | - |

**Table S5a**

| **Min-Dust** | |
| --- | --- |
| **High** | |
| Labourers in process and plant operations n.e.c. | - |
| Labourers in building and woodworking trades | - |
| Construction operatives n.e.c. | [47,62,74,76,99,105,107,111,113,395-400] |
| Textile process operatives | - |
| Coal mine operatives | [401] |

**Table S5b**

| **Min-Dust** | |
| --- | --- |
| **Medium** | |
| Metal working production and maintenance fitters | [402,403] |
| Motor mechanics, auto engineers | - |
| Cleaners, domestics | [138,404] |
| Farm workers | [147,341] |
| Painters and decorators | [257,404] |

**Table S5c**

| **Min-Dust** | |
| --- | --- |
| **Low** | |
| Laboratory technicians | [405] |
| NCOs and other ranks | [406] |
| Science and engineering technicians n.e.c. | - |
| Mechanical engineers | - |
| Assemblers and routine operatives n.e.c. | [407] |

**Table S5d**

| **Min-Dust** | |
| --- | --- |
| **Unexposed** | |
| Secondary education teaching professionals | - |
| Primary and nursery education teaching professionals | - |
| Personal assistants and other secretaries | - |
| Nurses | - |
| Local government clerical officers and assistants | - |

| **Table S6a** | | | | | | | | |
| --- | --- | --- | --- | --- | --- | --- | --- | --- |
| **Dust** | | | | | | | | |
| **High** | | | | | | | | |
| **Job** | **No of**  **papers included** | **No of paper** | **Exposure studies** | **No of paper** | **Epi studies (health outcomes)** | **No of paper** | **Other (exposure to different hazardous agents)/ Reviews** | **Evidence** |
| Labourers in process and plant operations n.e.c. | 1 | - | - | - | - | 1 | Airborne crystalline silica concentrations (mean value of 0.048 mg/m^3^) at coal-fired power plants associated with coal fly ash | x |
| Carpenters and joiners | 37 | 9 | Wood dust* exposure mostly / Respirable dust and wood dust exposure among carpenters and construction industry and hardwood dust among joiners  Limit of 2mg/m^3^ for carpenter’s shop  *Wood dust levels*: above 3.5mg/m^3^ and sometimes exceeding 5mg/m^3^.  Exposure to wood dust among carpenters: 1.5 times the limit of 2mg/m^3^ | 24 | Wood dust can cause cytotoxic effects which may lead to inflammation, chromosomal instability (wood dust is human carcinogen), respiratory risks (rhinitis and asthma), sinonasal cancer, lip cancer, stomach cancer, lung cancer, adenocarcinoma, Ca incidence and risk, mortality, idiopathic pulmonary fibrosis (IPF), allergic dermatoses and symptoms  Lung cancer among carpenters: O.R.:1.3(1.0-1.7)  Adenocarcinoma among carpenters: O.R.: 1.6(1.0-2.5)  Lip cancer among carpenters: O.R.: 2.28(1.23-4.14) | 4 | Crystalline silica / asbestos at work sites / PAHs in wood dust  mesothelioma  During some operations wood produces 100 times more PAHs in dust | ✓ |
| Labourers in building and woodworking trades | 6 | 5 | Silica dust and respirable dust in construction industry  Respirable dust: 2.46mg/m^3^, 2.85mg/m^3^  Silica: 0.350mg/m^3^ | 1 | Lung and bladder cancer among employments in woodworking trades | - | - | ? |
| Construction operatives n.e.c. | 73 | 17 | Particulate matter, respirable dust, airborne ultrafine particles, inorganic dust, quartz exposure**, quartz containing dust, wood dust, inhalable cement dust, diesel exhaust exposure and airborne contaminants in tunnel and construction workers, stone dust, cement dust  Respirable dust in tunnel workers: 1.2-3.6 mg/m^3^  *Inhalable dust levels in construction site*: 0.05 to 34mg/m^3^, PM levels: 51-841 μg/m^3^, quartz levels up to 63 times the max allowable concentration (0.075mg/m3), 74μg/m^3^  Total and respirable dust GM: 3.5 and 1.2 mg/m^3^  Quartz and dust exceeding respirable exposures of 25.6 and 9.3 respectively | 41 | Ischaemic heart disease, inflammation, infectious pneumonias, COPD, decline in lung function, respiratory diseases, lung cancer, silicosis, sarcoidosis, asthma, chronic renal disease, range of autoimmune diseases, coccidioidomycosis, skin symptoms, eye injuries from dust  Particulate air pollution and ischaemic heart disease: R.R.: 1.13 (1.07-1.19)  Inorganic dust and IHD: R.R.: 1.07 (1.03-1.12)  Cement dust and adenocarcinoma: IRR: 4.5(1.4- 14.3)  Wood dust and adenocarcinoma: 4.8 (1.2- 19.4)  Cement dust and pharyngeal cancer: 1.9(1.2-3.1)  Wood dust and sinonasal Ca: O.R.: 4.0 (1.5-10.8) | 15 | Respirable crystalline silica, asbestos, inorganic dust  Crystalline silica GM: 0.30mg/m^3^  Total dust from block of flats GM:  1.9mg/m^3^ | ✓ |
| Construction trades n.e.c | 5 | - | - | 2 | Mineral, wood dust and brain cancer, dust and work-related asthma | 3 | Asbestos and asbestiform minerals and adverse effects | ? |

**Table S6b**

| **Dust** | | | | | | | | |
| --- | --- | --- | --- | --- | --- | --- | --- | --- |
| **Medium** | | | | | | | | |
| **Job** | **No of**  **papers included** | **No of paper** | **Exposure studies** | **No of paper** | **Epi studies (health outcomes)** | **No of paper** | **Other (exposure to different hazardous agents)/ Reviews** | **Evidence** |
| Metal working production and maintenance fitters | 12 | 8 | WC-Co dust, mixed dust, inhalable and total dust, manganese dust (particle size), airborne contaminants, inhalable dust which contains nickel  *Dust exposure levels*: 0.001 to 83mg/m^-3^  Nickel exposure levels GM: 0.34microgram cm ^(-2)^  Mn respirable dust: 0.04 mg/m^3^ | 1 | “hard metal lung disease”, lung cancer, hard metal fibrosis, neurotoxic effects, IPF | 3 | Cobalt exposure and lung cancer and hard metal fibrosis, chromium exposure  *Airborne cobalt exposure levels*: 0.002 to 0.028mg/m^3^ | ✓ |
| Motor mechanics, auto engineers | 5 | 2 | Air particulates,  Indoor mass concentration of PM10: 22 and 36 times higher than outdoors | 1 | Bladder cancer, lung health and chronic obstructive lung disease and high exposures to pollutants (gases and particulate matter) | 2 | Asbestos and brake mechanics | ? |
| Cleaners, domestics | 3 | 2 | Dust, VOCs, quartz, concrete dust containing quartz in construction site cleaners  Highest dust concentrations in dry sweeping  *Dust concentration levels*: 32mg/m^3^  *Respirable quartz levels*: 0.53 mg/m^3^ | 1 | Allergies, asthma, ischemic heart disease | - | - | x |
| Farm workers | 19 | 7 | Agricultural dust, soil dust and airborne particulate matter and seeding operations, inhalable dust, TVOCs and dairy farm workers, dust exposure and tobacco farm workers, air pollutant exposures and farmers, inorganic and organic dust, PM and endotoxin in total dust and swine barn workers  GM of inhalable dust: 1.5mg/m^3^  *Inhalable and respirable swine dust*: 0.25-7.6 mg/m^3^ and 0.01-3.4 mg/m^3^ | 10 | Respirable dust, total dust, endotoxin in total dust  Respiratory symptoms, asthma, COPD, chronic bronchitis, fibrosis, cancers  *Airborne dust levels in pig farms*: 1.66 to 21.04 mg/m^3^  Respirable dust: 1-5mg/m^3^  Total dust: >=20 mg/m^3^ | 2 | crystalline silica, humulus lupulus and respiratory disease in hop workers | ✓ |
| Painters and decorators | 7 | 1 | Dust, VOCs  Dust exposure levels: 10 mg/m^3^ | 2 | Dust  *Health problems*: nasal symptoms, acute and chronic effects  chromosomal aberrations, nasal mucosal disturbances | 4 | Lead from lead-based paints and lead poisoning, titanium dioxide nanoparticles in paint dust  airborne concentrations of lead- chromium- cadmium, solvents  *Average emission factor*: 20.400 mg/m2 for lead, 75 mg/m^2^ for chromium, 16 mg/m^2^ for cadmium | ? |

**Table S6c**

| **Dust** | | | | | | | | |
| --- | --- | --- | --- | --- | --- | --- | --- | --- |
| **Low** | | | | | | | | |
| **Job** | **No of**  **papers included** | **No of paper** | **Exposure studies** | **No of paper** | **Epi studies (health outcomes)** | **No of paper** | **Other (exposure to different hazardous agents)/ Reviews** | **Evidence** |
| Nurses | 7 | - | - | 3 | Respiratory symptoms and asthma from VGDF occupational exposure, drug dust exposure and occupational contact dermatitis | 4 | PM2.5 and second-hand smoke and health staff in mental health units, allergic hand dermatitis from airborne spread of rubber accelerators, ispaghula powder | ? |
| Laboratory technicians | 8 | 1 | PM emissions from non-road vehicles and laboratory technicians | 4 | Dust exposure, quartz and pneumoconiosis (5.6%)  Mineralogical dust exposure, PM2.5 and lung health  PM2.5 ranged from 26-664 mg/m^3^ | 3 | Fibrinogen dusts and dental laboratory technicians, crystalline silica dust and silicosis among dental laboratory technicians  Hard metal dust and silica particles and aluminium | ? |
| NCOs and other ranks | 2 |  | - | - | - | 2 | Gun powder, silica dust, carbon nanoparticles | x |
| Science and engineering technicians n.e.c. | - | - | - | - | - | - | - | x |
| Medical practitioners | - | - | - | - | - | - | - | x |

**Table S6d**

| **Dust** | | | | | | | | |
| --- | --- | --- | --- | --- | --- | --- | --- | --- |
| **Unexposed** | | | | | | | | |
| **Job** | **No of**  **papers included** | **No of paper** | **Exposure studies** | **No of paper** | **Epi studies (health outcomes)** | **No of paper** | **Other (exposure to different hazardous agents)/ Reviews** | **Evidence** |
| Secondary education teaching professionals | - | - | - | - | - | - | - | x |
| Primary and nursery education teaching professionals | - | - | - | - | - | - | - | x |
| Personal assistants and other secretaries | 1 | - | - | 1 | Private secretaries and cancer of the mouth | - | - | x |
| Local government clerical officers and assistants | - | - | - | - | - | - | - | x |
| Sales and retail assistants | - | - | - | - | - | - | - | x |

**Table S7a**

| **Fumes** | | | | | | | | |
| --- | --- | --- | --- | --- | --- | --- | --- | --- |
| **High** | | | | | | | | |
| **Job** | **No of**  **papers included** | **No of paper** | **Exposure studies** | **No of paper** | **Epi studies (health outcomes)** | **No of paper** | **Other (exposure to different hazardous agents)/ Reviews** | **Evidence** |
| Welding trades | 3 | 1 | Chemical, welding and thermal cutting fumes | 2 | Exposure to fumes and electromagnetic radiation  *Health outcomes*: cancer incidence | - | - | ? |
| Fire service officers (leading fire officer and below) | 4 | 1 | Fumes and smoke | 1 | Smoke from burning plastics  *Health outcomes:* respiratory symptoms, transient hypoxemia | 2 | Cadmium, sulphonated castor oil, fumes of toluene and damage of the respiratory tract | ? |
| Sheet metal workers | 2 | - | - | 1 | Welding fumes and prevalence of obstructive pulmonary disease/ lung disease | 1 | Zinc oxide fume inhalation does not affect sheet metal workers | x |
| Moulders, core makers, die casters | 1 | - | - | - | - | 1 | Metallic components of fumes and ozone or compounds of nitrogen | x |
| Smiths and forge workers | 2 | - | - | - | - | 2 | Zinc oxide that exceeds the MAC value among ship smiths | x |

**Table S7b**

| **Fumes** | | | | | | | | |
| --- | --- | --- | --- | --- | --- | --- | --- | --- |
| **Medium** | | | | | | | | |
| **Job** | **No of**  **papers included** | **No of paper** | **Exposure studies** | **No of paper** | **Epi studies (health outcomes)** | **No of paper** | **Other (exposure to different hazardous agents)/ Reviews** | **Evidence** |
| Labourers in process and plant operations n.e.c. | - | - | - | - | - | - | - | x |
| Electrical/  electronics technicians | 1 | - | - | 1 | Electromagnetic radiation and soldering fumes  *Health outcomes*: brain tumor | - | - | x |
| Chefs, cooks | 12 | 4 | Cooking fumes, polycyclic aromatic hydrocarbons (PAHs) from cooking- particulate matter, ultrafine particles during cooking, fat aerosols  Maximum values of PAHs: 270-300 ng/m^3^ air  Level of total particles: 2.2-4.2 mg/m^3^  Arithmetic mean of fat aerosols: 0.62 mg/m^3^ | 7 | Cooking fumes, polycyclic aromatic hydrocarbons (PAHs) from cooking fumes, cooking oil fumes (COFs), mutagenic and carcinogenic air pollutants  *Health outcomes*: chronic bronchitis, respiratory symptoms, dyspnea, pulmonary irritation, lung cancer, DNA damage | 1 | Acrolein from fumes is a carcinogen (report) | ✓ |
| Motor mechanics, auto engineers | - | - | - | - | - | - | - | x |
| Labourers in building and woodworking trades | 1 | 1 | Asphalt fumes in labourers in road construction project  Exposure ranged from 0.20 to 1.13 mg/m^3^ | - | - | - | - | x |

**Table S7c**

| **Fumes** | | | | | | | | |
| --- | --- | --- | --- | --- | --- | --- | --- | --- |
| **Low** | | | | | | | | |
| **Job** | **No of**  **papers included** | **No of paper** | **Exposure studies** | **No of paper** | **Epi studies (health outcomes)** | **No of paper** | **Other (exposure to different hazardous agents)/ Reviews** | **Evidence** |
| NCOs and other ranks | 3 | - | - | 2 | Exposure to fumes from military weapons and munitions  Health outcomes: respiratory hazard and multisymptomatic conditions | 1 | Soldiers exposed to fumes, toxic dust (a review) | ? |
| Mechanical engineers | - | - |  | - | - | - | - | x |
| Assemblers and routine operatives n.e.c. | 2 | 1 | Smoke/ fume/ gas and assemblers | 1 | Soldering fumes, electromagnetic radiation, solvents and chemicals among assemblers  Health outcomes: brain tumor | - | - | ? |
| Metal working production + maintenance fitters | - | - | - | - | - | - | - | - |
| Police officers (sergeant and below) | 1 | - | - | 1 | Toxic fumes  Health outcomes: reactive airway dysfunction syndrome (RADS) (bronchial hyperreactivity and asthmatic symptoms develop in previously healthy individuals after a single large exposure to an irritating gas, fume, or vapor) | - | - | x |

**Table S7d**

| **Fumes** | | | | | | | | |
| --- | --- | --- | --- | --- | --- | --- | --- | --- |
| **Unexposed** | | | | | | | | |
| **Job** | **No of**  **papers included** | **No of paper** | **Exposure studies** | **No of paper** | **Epi studies (health outcomes)** | **No of paper** | **Other (exposure to different hazardous agents)/ Reviews** | **Evidence** |
| Secondary education teaching professionals | - | - | - | - |  | - | - | x |
| Primary and nursery education teaching professionals | - | - | - | - |  | - | - | x |
| Personal assistants and other secretaries | - | - | - | - |  | - | - | x |
| Nurses | - | - | - | - |  | - | - | x |
| Local government clerical  officers and assistants | - | - | - | - |  | - | - | x |

**Table S8a**

| **Diesel** | | | | | | | | |
| --- | --- | --- | --- | --- | --- | --- | --- | --- |
| **High** | | | | | | | | |
| **Job** | **No of**  **papers included** | **No of paper** | **Exposure studies** | **No of paper** | **Epi studies (health outcomes)** | **No of paper** | **Other (exposure to different hazardous agents)/ Reviews** | **Evidence** |
| - | - | - | - | - |  | - | - | x |
| - | - | - | - | - |  | - | - | x |
| - | - | - | - | - |  | - | - | x |
| - | - | - | - | - |  | - | - | x |
| - | - | - | - | - |  | - | - | x |

**Table S8b**

| **Diesel** | | | | | | | | |
| --- | --- | --- | --- | --- | --- | --- | --- | --- |
| **Medium** | | | | | | | | |
| **Job** | **No of**  **papers included** | **No of paper** | **Exposure studies** | **No of paper** | **Epi studies (health outcomes)** | **No of paper** | **Other (exposure to different hazardous agents)/ Reviews** | **Evidence** |
| Motor mechanics, auto engineers | 11 | 2 | Mixtures of diesel and gasoline exhaust | 7 | Diesel engine exhaust, automobile exhaust  Health outcomes: cytotoxic and genotoxic effects, lung cancer, laryngeal cancer, pulmonary function abnormalities- though not causal evidence that long term exposure to DE increases mutagenicity and lung cancer in all studies | 2 | Diesel and gasoline emissions and increased cancer risk | ✓ |
| Ship and hovercraft officers | - | - | - | - | - | - | - | x |
| Coal mine operatives | 21 | 15 | Diesel engine particulate matter pollution (DPM) (found in the respirable dust)  Average diesel particulate exposure: 0.1-2.1 mg/m^3^ | 5 | Coal mine dust and contaminations of dust by diesel emissions, diesel engine emissions (DEE)  Health outcomes: fibrogenic and cytotoxic, DNA adducts and lung cancer, chronic respiratory effects, COPD | 1 | Diesel exhaust aerosols (review) | ✓ |
| Garage managers and proprietors | 5 papers about garage WORKERS! |  |  |  |  |  |  | x |
| Fire service officers (leading fire officer and below) | 1 | - | - | 1 | Diesel engine exhaust exposure  Health outcome: rate of bronchopulmonary carcinoma | - | - | x |

**Table S8c**

| **Diesel** | | | | | | | | |
| --- | --- | --- | --- | --- | --- | --- | --- | --- |
| **Low** | | | | | | | | |
| **Job** | **No of**  **papers included** | **No of paper** | **Exposure studies** | **No of paper** | **Epi studies (health outcomes)** | **No of paper** | **Other (exposure to different hazardous agents)/ Reviews** | **Evidence** |
| NCOs and other ranks | - | - | - | - | - | - | - | x |
| Mechanical engineers | 2 | 1 | PM2.5 and black carbon in maintenance engineers  Maintenance engineers had the highest average levels of exposure to both: PM2.5: 76 μg/m^3^ and BC: 9.3 μg/m^3^ | 1 | Diesel exhaust and operating engineers  Health outcome: lung function and asthma | - | - | x |
| Police officers (sergeant and below) | - | - | - | - | - | - | - | x |
| Sales representatives | - | - | - | - | - | - | - | x |
| Managers in construction | 5 papers about construction WORKERS! |  |  | 1 | Diesel exhaust gases  Health outcomes: chromosome aberration (CA) |  | Respirable elemental carbon and respirable crystalline silica | x |

**Table S8d**

| **Diesel** | | | | | | | | |
| --- | --- | --- | --- | --- | --- | --- | --- | --- |
| **Unexposed** | | | | | | | | |
| **Job** | **No of**  **papers included** | **No of paper** | **Exposure studies** | **No of paper** | **Epi studies (health outcomes)** | **No of paper** | **Other (exposure to different hazardous agents)/ Reviews** | **Evidence** |
| Secondary education teaching professionals | - | - | - | - |  | - | - | x |
| Primary and nursery education teaching professionals | - | - | - | - |  | - | - | x |
| Personal assistants and other secretaries | - | - | - | - |  | - | - | x |
| Nurses | - | - | - | - |  | - | - | x |
| Local government clerical  officers and assistants | - | - | - | - |  | - | - | x |

**Table S9a**

| **Bio-Dust** | | | | | | | | |
| --- | --- | --- | --- | --- | --- | --- | --- | --- |
| **High** | | | | | | | | |
| **Job** | **No of**  **papers included** | **No of paper** | **Exposure studies** | **No of paper** | **Epi studies (health outcomes)** | **No of paper** | **Other (exposure to different hazardous agents)/ Reviews** | **Evidence** |
| Carpenters and joiners | 29 | 7 | Inhalable wood dust, wood in inhalable construction dust  Levels of wood dust: exceeding 5 mg/m^3^  Average exposure to dust (8 hours): 0.8 to 11.6 mg/m3 with geometric mean: 3.3 mg/m^3^  Wood dust at construction work sites: 0.065 mg | 20 | Wood dust  Health outcomes: allergies, occupational rhinitis, asthma, nasal adenocarcinoma, lung cancer, bone cancer, emphysema, cytotoxic effect, inflammation, idiopathic pulmonary fibrosis, allergic contact dermatitis, lip cancer, nasopharyngeal cancer, stomach cancer | 2 | Wood product industries, asbestos, formaldehyde, carcinogenic PAHs in wood dust  Asbestosis, mesothelioma | ✓ |
| Textile process operatives | 8 | - | - | 6 | Organic dusts, cotton dust  Health outcome: exercise-induced bronchoconstriction, COPD, inflammation, bronchitis, adenocarcinoma, squamous cell carcinomas | 2 | Organic dust on chronic respiratory disease and byssinosis (reviews) | ? |
| Food, drink and tobacco process operatives | - | - | - | - | - | - | - | x |
| Bakers, flour confectioners | 36 | 13 | Flour dust, airborne molds  *Inhalable dust levels for bakers*: 3mg/m^3^, 8.2mg/m^3^, sometimes exceeded 10 mg/m^3^,  mean alpha-amylase exposure: 22.0 ng/m^3^,  GM dust exposure for bakers: 1.2 mg/m^3^ | 20 | Flour dust, wheat allergen, serum aflatoxin B1 (AFB1), liver enzymes, soybean dust, dust mites, a-amylase  *Health outcomes*: nasal mucosal inflammation, asthma, allergic obstructive airway disease, pulmonary function impairment, allergic respiratory disease, immunological disorders  During a work shift exposure concentration of 0.2 mg/m^3^ wheat allergen and 0.5 mg/m^3^ inhalable dust  Average wheat exposure levels: 25-30 microg/m^3^  Dough makers or bread formers personal inhalable dust measurements: 1.0 to 3.8 mg/m^3^ | 3 | Flour dust, wheat flour dust reviews  Occupational asthma in bakers (book) | ✓ |
| Furniture makers, other craft woodworkers | 40 | 4 | Organic wood dust, hardwood dust  Average GM exposures in furniture making: 0.6 mg/m^3^  *Cumulative dust exposure*: 3.75 mg x year x m(-3)  Overall GM exposure to dust: 0.96 mg/m^3^ | 34 | Organic wood dust, hardwood dust during furniture  Production  *Health outcomes*: COPD, sinonasal adenocarcinoma, respiratory symptoms, rhinitis, asthma, lung function decline, DNA damage, acute nasal obstruction, cancer | 2 | Woodworkers and nasal cancer (review) | ✓ |

**Table S9b**

| **Bio-Dust** | | | | | | | | |
| --- | --- | --- | --- | --- | --- | --- | --- | --- |
| **Medium** | | | | | | | | |
| **Job** | **No of**  **papers included** | **No of paper** | **Exposure studies** | **No of paper** | **Epi studies (health outcomes)** | **No of paper** | **Other (exposure to different hazardous agents)/ Reviews** | **Evidence** |
| Labourers in building and woodworking trades | - | - | - | - | - | - | - | x |
| Farm workers | 10 | 1 | Organic dust  *Total concentration of airborne conidia*: 1.1 x 10(4) to 3.9 x 15(5) per m^3^ and *airborne dust concentration* ranged from 0.08 to 0.21 mg/m^3^ | 7 | Organic dust (poultry residues, molds, feathers, microorganisms  *Health outcome*: byssinosis, rhinitis, asthma, COPD, organic dust toxic syndrome, pneumonitis, chronic bronchitis, lung disease, rheumatoid arthritis, pulmonary function reductions | 2 | Organic dust and respiratory reviews | ✓ |
| Farmers | 53 | 9 | Organic dust, allergenic mites, thoracic dust, endotoxin, total volatile organic compounds, airborne microorganisms  Mean concentration of thoracic dust: 0.24 mg/m^3^  GM of inhalable dust: 4.4 mg/ m^3^  Levels of total and respirable dust respectively: 701.1 EU/m^3^ LPS and 15.8 EU/m^3^ LPS  More than 50% of the exposure measurements for endotoxin and organic dust exceeded recommended health occupational exposure limits sometimes! | 3 | Organic dust, allergenic mites, grain dust, fungal spores  Health outcomes: asthma, adverse respiratory health effects, organic dust toxic syndrome, cough, throat irritation, COPD, diffuse alveolar haemorrhage, allergic alveolitis, chronic bronchitis, local and systemic inflammation, emphysema, bronchial hyperreactivity  Dust: 0.04 to 2 mg/m^3^/ 11.9 (2.8) mg/m^3^ | 7 | Organic dust and endotoxin reviews and reports | ✓ |
| Weighers, graders, sorters | - | - | - | - | - | - | - | x |
| Paper and wood machine operatives | - | - | - | - | - | - | - | x |

**Table S9c**

| **Bio-Dust** | | | | | | | | |
| --- | --- | --- | --- | --- | --- | --- | --- | --- |
| **Low** | | | | | | | | |
| **Job** | **No of**  **papers included** | **No of paper** | **Exposure studies** | **No of paper** | **Epi studies (health outcomes)** | **No of paper** | **Other (exposure to different hazardous agents)/ Reviews** | **Evidence** |
| Nurses | - | - | - | - |  | - | - | x |
| Laboratory technicians | - | - | - | - |  | - | - | x |
| Medical practitioners | - | - | - | - |  | - | - | x |
| Nursing auxiliaries and assistants | - | - | - | - |  | - | - | x |
| Care assistants and home carers | - | - | - | - |  | - | - | x |

**Table S9d**

| **Bio-Dust** | | | | | | | | |
| --- | --- | --- | --- | --- | --- | --- | --- | --- |
| **Unexposed** | | | | | | | | |
| **Job** | **No of**  **papers included** | **No of paper** | **Exposure studies** | **No of paper** | **Epi studies (health outcomes)** | **No of paper** | **Other (exposure to different hazardous agents)/ Reviews** | **Evidence** |
| Secondary education teaching professionals | - | - | - | - |  | - | - | x |
| Primary and nursery education teaching professionals | - | - | - | - |  | - | - | x |
| Personal assistants and other secretaries | - | - | - | - |  | - | - | x |
| Local government clerical  officers and assistants | - | - | - | - |  | - | - | x |
| Sales and retail assistants | - | - | - | - |  | - | - | x |

**Table S10a**

| **Min-Dust** | | | | | | | | |
| --- | --- | --- | --- | --- | --- | --- | --- | --- |
| **High** | | | | | | | | |
| **Job** | **No of**  **papers included** | **No of paper** | **Exposure studies** | **No of paper** | **Epi studies (health outcomes)** | **No of paper** | **Other (exposure to different hazardous agents)/ Reviews** | **Evidence** |
| Labourers in process and plant operations n.e.c. | - | - | - | - | - | - | - | x |
| Labourers in building and woodworking trades | - | - | - | - | - | - | - | x |
| Construction operatives n.e.c. | 15 | 4 | Mineral dust, high respirable quartz exposures  Geometric mean exposure to quartz for tunnel workers: 0.035 mg/m^3^  Inhalable exposure to quartz exceeded respirable exposures by a factor of 25.6 | 6 | Respirable dust and quartz  Health outcomes: IDR, respiratory symptoms, decline in lung function, silicosis, thrombosis  Geometric mean of a-quartz concentrations: 74 μg/m^3^ | 5 | Silica dust and asbestos fibres in construction and silicosis  Dust and quartz exposure levels: exceeding the limit values of 3.0 and 0.05mg/m ^3^ respectively | ✓ |
| Textile process operatives | - | - | - | - | - | - | - | x |
| Coal mine operatives | 1 | 1 | Respirable dust and quartz exposure | - | - | - | - | x |

**Table S10b**

| **Min-Dust** | | | | | | | | |
| --- | --- | --- | --- | --- | --- | --- | --- | --- |
| **Medium** | | | | | | | | |
| **Job** | **No of**  **papers included** | **No of paper** | **Exposure studies** | **No of paper** | **Epi studies (health outcomes)** | **No of paper** | **Other (exposure to different hazardous agents)/ Reviews** | **Evidence** |
| Metal working production and maintenance fitters | 2 | 1 | Quartz in iron foundries | - | - | 1 | Airborne silica dust exposure in metal industry | ? |
| Motor mechanics, auto engineers | - | - | - | - | - | - | - | x |
| Cleaners, domestics | 2 | 1 | Quartz  Level of respirable quartz exposure while dry sweeping in construction sites: 0.53 mg/m^3^ | 1 | Mineral dusts  Cleaners and asthma O.R.: 1.97 (1.33-2.92) | - | - | ? |
| Farm workers | 2 | - | - | 2 | Mineral dust- inorganic dust  Health outcomes: chronic bronchitis, interstitial fibrosis, and chronic obstructive pulmonary disease, rheumatoid arthritis | - | - | ? |
| Painters and decorators | 2 | - | - | 2 | Mineral dust  Adjusted O.R.: 3.8(1.21-12.0) for workers exposed to high  Levels and COPD  O.R. for asthma and painters: 2.34 (1.04- 5.28)  Health outcomes: COPD and asthma | - | - | ? |

**Table S10c**

| **Min-Dust** | | | | | | | | |
| --- | --- | --- | --- | --- | --- | --- | --- | --- |
| **Low** | | | | | | | | |
| **Job** | **No of**  **papers included** | **No of paper** | **Exposure studies** | **No of paper** | **Epi studies (health outcomes)** | **No of paper** | **Other (exposure to different hazardous agents)/ Reviews** | **Evidence** |
| Laboratory technicians | 1 | - | - | - | - | 1 | silica and chromium-cobalt alloy by inhalation | x |
| NCOs and other ranks | 1 | - | - | 1 | Sailors and mineral dust  Health outcomes: gastric cancer | - | - | x |
| Science and engineering technicians n.e.c. | - | - | - | - | - | - | - | x |
| Mechanical engineers | - | - |  | - | - | - | - | x |
| Assemblers and routine operatives n.e.c. | 1 | - | - | - | - | 1 | Artificial quartz aggregates (AQA)  And silicosis in assembling work | x |

**Table S10d**

| **Min-Dust** | | | | | | | | |
| --- | --- | --- | --- | --- | --- | --- | --- | --- |
| **Unexposed** | | | | | | | | |
| **Job** | **No of**  **papers included** | **No of paper** | **Exposure studies** | **No of paper** | **Epi studies (health outcomes)** | **No of paper** | **Other (exposure to different hazardous agents)/ Reviews** | **Evidence** |
| Secondary education teaching professionals | - | - | - | - |  | - | - | x |
| Primary and nursery education teaching professionals | - | - | - | - |  | - | - | x |
| Personal assistants and other secretaries | - | - | - | - |  | - | - | x |
| Nurses | - | - | - | - |  | - | - | x |
| Local government clerical  officers and assistants | - | - | - | - |  | - | - | x |

**Table S11.** Exposure levels and proportions exposed according to ACE JEM and independent exposure assessor.

| **Job codes** | **Job** | **Exposure level (H)igh, (M)edium, (L)ow, None(0) according to John W. Cherrie** | **Exposure level (H, M, L, None) according to ACE-JEM** | **Proportion exposed (<5%=0, 5-19%=1, 20-49%=2, >50%=3)**  **John W. Cherrie** | **Proportion exposed (<5%=0, 5-19%=1, 20-49%=2, >50%=3)**  **ACE-JEM** |
| --- | --- | --- | --- | --- | --- |
| 9121 | Labourers in building and woodworking trades (same as elementary construction occupations and construction operatives) | Dust H  Fumes M  Diesel H  Min dust H  Bio dust M | H  M  L  H  M | 3  1  2  3  1 | 3  3  3  3  3 |
| 5315 | Carpenters and joiners | Dust H  Fumes 0  Diesel 0  Min dust M  Bio dust H | H  0  0  0  H | 3  0  0  0  3 | 3  0  0  0  3 |
| 5319 | Construction trades n.e.c. | Dust H  Fumes M  Diesel H  Min dust H  Bio dust M | H  0  0  M  0 | 3  1  2  3  0 | 3  0  0  3  0 |
| 5231 | Motor mechanics, auto engineers | Dust M  Fumes H  Diesel H  Min dust 0  Bio dust 0 | H  0  0  M  0 | 0  1  1  0  0 | 3  0  0  3  0 |
| 9233 | Cleaners, domestics | Dust L  Fumes 0  Diesel 0  Min dust 0  Bio dust L | M  0  0  M  0 | 3  0  0  0  3 | 3  0  0  3  0 |
| 9111 | Farm workers | Dust H  Fumes L  Diesel M  Min dust M  Bio dust H | M  L  L  M  M | 2  0  1  1  2 | 3  2  2  3  3 |
| 5323 | Painters and decorators | DUST M  Fumes 0  Diesel 0  Min dust 0  Bio dust 0 | M  0  0  L  0 | 1  0  0  0  3 | 3  0  0  3  0 |
| 3211 | Nurses | DUST L  Fumes 0  Diesel 0  Min dust 0  Bio dust L | L  0  0  0  L | 1  0  0  0  1 | 2  0  0  0  2 |
| 3111 | Laboratory technicians | DUST L  Fumes L  Diesel L  Min dust L  Bio dust L | L  0  0  L  L | 1  1  1  1  1 | 1  0  0  1  1 |
| 3311 | NCOs and other ranks | DUST 0  Fumes 0  Diesel L  Min dust 0  Bio dust 0 | L  L  L  L  0 | 0  0  1  0  3 | 1  1  1  1  0 |
| 3119 | Science and engineering technicians n.e.c. | DUST L  Fumes L  Diesel L  Min dust L  Bio dust L | L  0  0  L  0 | 1  1  1  1  1 | 0  0  0  1  0 |
| 2211 | Medical practicioners | DUST 0  Fumes 0  Diesel 0  Min dust 0  Bio dust 0 | L  0  0  0  L | 0  0  0  0  0 | 2  0  0  0  2 |
| 2314 | Secondary education teaching professionals | Dust 0  Fumes 0  Diesel 0  Min dust 0  Bio dust 0 | 0  0  0  0  0 | 0  0  0  0  0 | 0  0  0  0  0 |
| 2315 | Primary and nursery education teaching professionals | Dust 0  Fumes 0  Diesel 0  Min dust 0  Bio dust 0 | 0  0  0  0  0 | 0  0  0  0  0 | 0  0  0  0  0 |
| 4215 | Personal assistants and other secretaries | Dust 0  Fumes 0  Diesel 0  Min dust 0  Bio dust 0 | 0  0  0  0  0 | 0  0  0  0  0 | 0  0  0  0  0 |
| 4113 | Local government clerical officers and assistants | Dust 0  Fumes 0  Diesel 0  Min dust 0  Bio dust 0 | 0  0  0  0  0 | 0  0  0  0  0 | 0  0  0  0  0 |
| 7111 | Sales and retail assistants | Dust 0  Fumes 0  Diesel 0  Min dust 0  Bio dust 0 | 0  0  0  0  0 | 0  0  0  0  0 | 0  0  0  0  0 |
| 5215 | Welding trades | Dust H  Fumes H  Diesel 0  Min dust 0  Bio dust 0 | M  H  0  M  0 | 3  3  0  0  0 | 3  3  3  3  0 |
| 3313 | Fire service officers (leading fire officer and below) | Dust H  Fumes L  Diesel M  Min dust L  Bio dust L | M  H  M  M  0 | 1  1  1  1  1 | 3  3  3  3  0 |
| 5213 | Sheet metal workers | Dust H  Fumes H  Diesel 0  Min dust 0  Bio dust 0 | M  H  0  M  0 | 1  2  0  0  0 | 3  3  0  3  0 |
| 5212 | Moulders, core makers, die casters | Dust H  Fumes H  Diesel 0  Min dust M  Bio dust 0 | H  H  0  H  0 | 3  3  0  3  0 | 3  3  0  3  0 |
| 5211 | Smiths and forge workers | Dust M  Fumes L  Diesel 0  Min dust 0  Bio dust 0 | M  H  0  M  0 | 3  3  0  0  0 | 3  3  0  3  0 |
| 3112 | Electrical/electronics technicians | Dust M  Fumes M  Diesel L  Min dust 0  Bio dust 0 | 0  M  0  0  0 | 1  1  0  0  0 | 0  1  0  0  0 |
| 5434 | Chefs, cooks | Dust L  Fumes L  Diesel 0  Min dust 0  Bio dust H | L  M  0  0  L | 1  1  0  0  1 | 2  3  0  0  2 |
| 2122 | Mechanical engineers | Dust M  Fumes M  Diesel M  Min dust 0  Bio dust 0 | L  L  L  L  0 | 1  1  1  0  0 | 1  2  2  1  0 |
| 8139 | Assemblers and routine operatives n.e.c. | Dust M  Fumes M  Diesel 0  Min dust 0  Bio dust 0 | L  L  0  L  0 | 1  0  0  0  0 | 3  2  0  3  0 |
| 3312 | Police officers (sergeant and below) | Dust L  Fumes L  Diesel M  Min dust L  Bio dust 0 | 0  L  L  0  0 | 0  0  1  0  0 | 0  1  1  0  0 |
| 8113 | Textile process operatives | Dust H  Fumes 0  Diesel 0  Min dust 0  Bio dust H | H  0  0  H  H | 3  0  0  0  3 | 3  0  0  2  3 |
| 8122 | Coal mine operatives | Dust H  Fumes L  Diesel M  Min dust H  Bio dust 0 | H  M  M  H  0 | 3  0  1  3  0 | 3  3  3  3  0 |
| 3542 | Sales representatives | Dust 0  Fumes 0  Diesel M  Min dust 0  Bio dust 0 | 0  L  L  0  0 | 0  0  1  0  0 | 0  1  1  0  0 |
| 1122 | Managers in construction | Dust M  Fumes L  Diesel M  Min dust M  Bio dust 0 | L  L  L  L  L | 1  0  1  1 | 2  1  1  2  1 |
| 9139 | Labourers in process and plant operations n.e.c. | Dust H  Fumes H  Diesel M  Min dust H  Bio dust 0 | H  M  L  H  0 | 3  2  0  1  0 | 3  3  3  3  0 |
| 5223 | Metal working production and maintenance fitters | Dust M  Fumes M  Diesel 0  Min dust 0  Bio dust 0 | M  L  0  M  0 | 2  2  0  0  0 | 3  2  0  3  0 |
| 3513 | Ship and hovercraft officers | Dust H  Fumes M  Diesel H  Min dust L  Bio dust L | 0  M  M  0  0 | 1  1  3  1  1 | 0  0  1  0  0 |
| 1232 | Garage managers and proprietors | Dust L  Fumes M  Diesel M  Min dust 0  Bio dust 0 | L  M  M  L  0 | 1  1  2  0  0 | 2  3  3  2  0 |
| 8111 | Food, drink and tobacco process operatives | Dust M  Fumes 0  Diesel 0  Min dust 0  Bio dust H | H  0  0  0  0 | 2  0  0  0  2 | 3  0  0  0  3 |
| 5432 | Bakers, flour confectioners | Dust M  Fumes 0  Diesel 0  Min dust 0  Bio dust H | H  0  0  0  0 | 3  0  0  0  3 | 3  0  0  0  3 |
| 5492 | Furniture makers, other craft woodworkers | DUST M  Fumes 0  Diesel 0  Min dust 0  Bio dust H (ASSUMING THAT WOOD DUST IS A BIODUST) | H  0  0  0  H | 3  0  0  0  3 | 3  0  0  0  3 |
| 5111 | Farmers (not farm workers) | DUST M  Fumes M  Diesel M  Min dust M  Bio dust H | M  M  M  0  M | 2  0  2  1  2 | 3  3  3  0  3 |
| 8134 | Weighers, graders, sorters | DUST M  Fumes 0  Diesel 0  Min dust 0  Bio dust M | M  0  0  M  M | 2  0  0  0  1 | 3  0  0  3  3 |
| 8121 | Paper and wood machine operatives | DUST M  Fumes 0  Diesel 0  Min dust 0  Bio dust M | H  0  0  0  0 | 3  0  0  0  3 | 3  0  0  0  3 |
| 6111 | Nursing auxiliaries and assistants | DUST L  Fumes 0  Diesel 0  Min dust 0  Bio dust L | L  0  0  0  L | 3  0  0  0  3 | 2  0  0  0  2 |
| 6115 | Care assistants and home carers | DUST L  Fumes 0  Diesel L  Min dust 0  Bio dust 0 | L  0  0  0  L | 1  0  1  0  0 | 1  0  0  0  1 |

**3. Figures**

**Figure S1.** Graphical representation of agreement (percentages) between assessors for level of exposure.

**
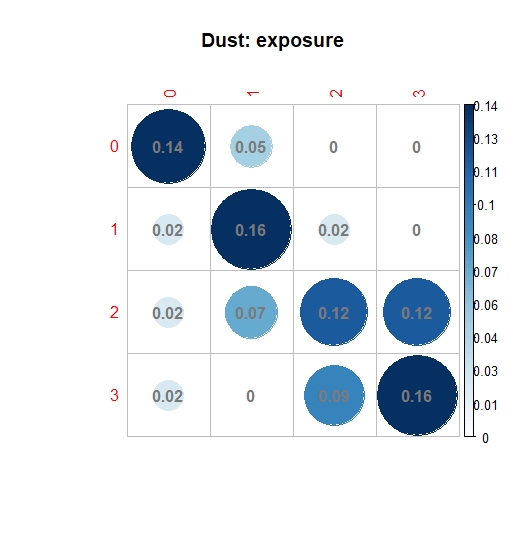

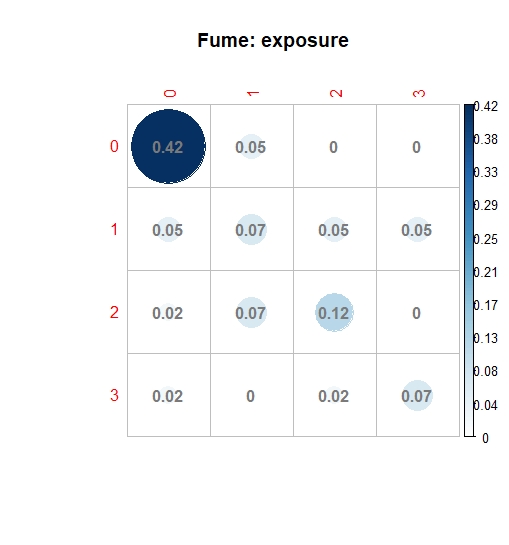

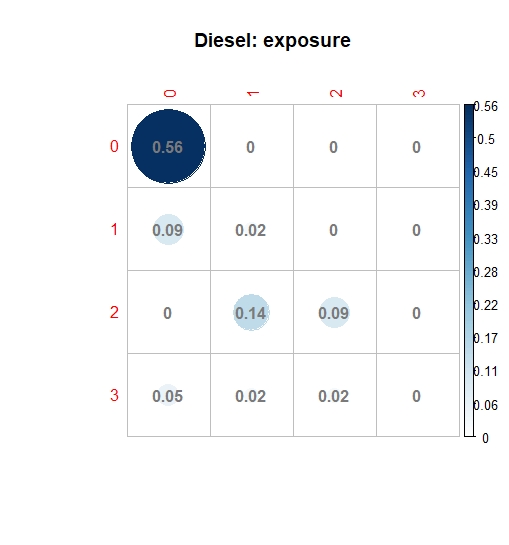
**

**
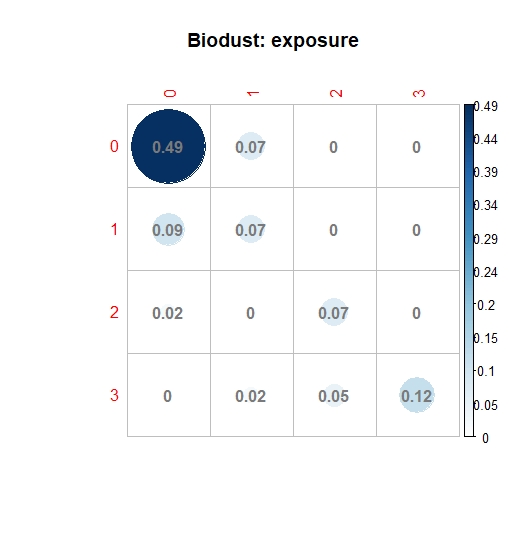

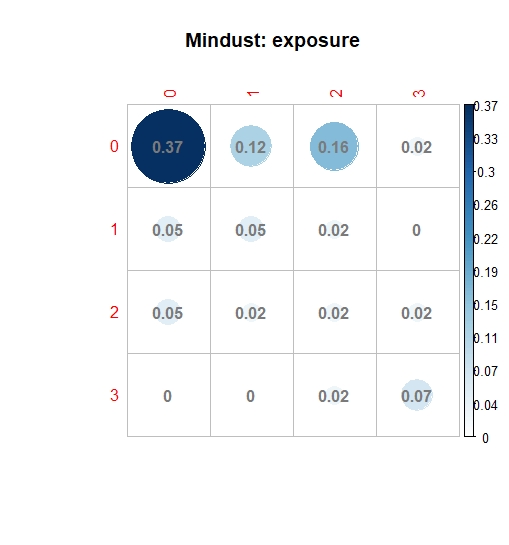
**

0: non-exposed, 1: low, 2: medium, 3: high

**Figure S2:** Graphical representation of agreement (percentages) between assessors for proportion exposed.

**
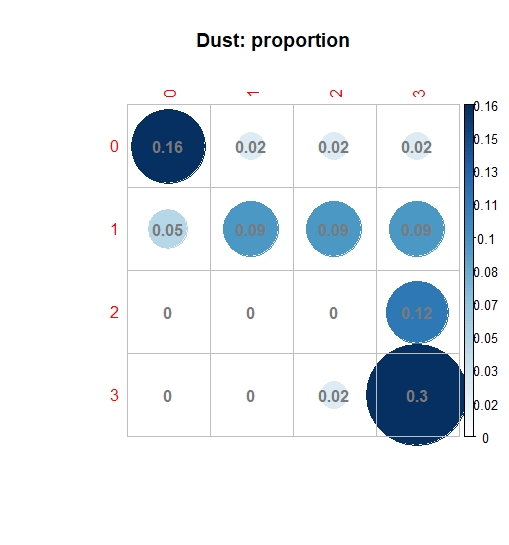

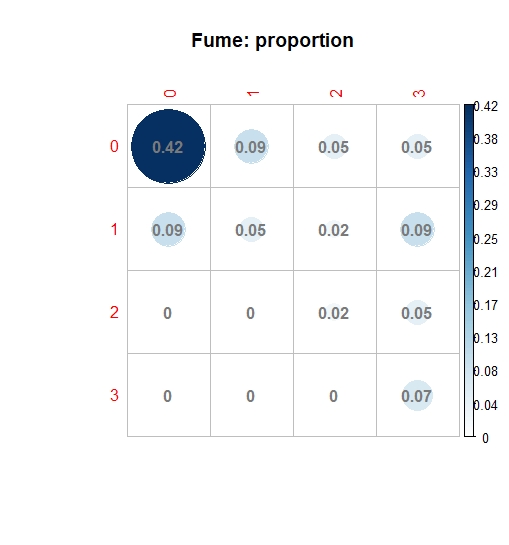
**

**
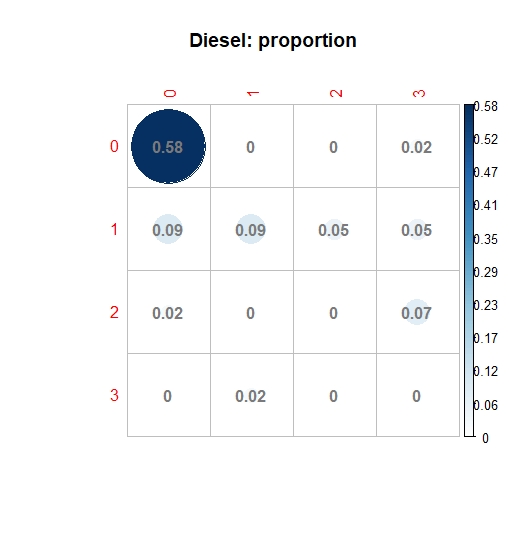

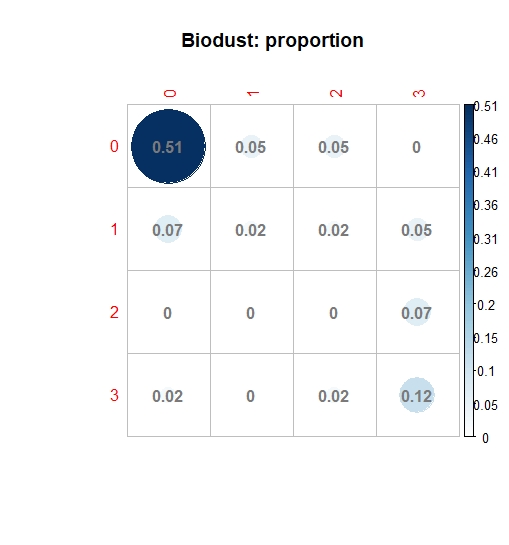

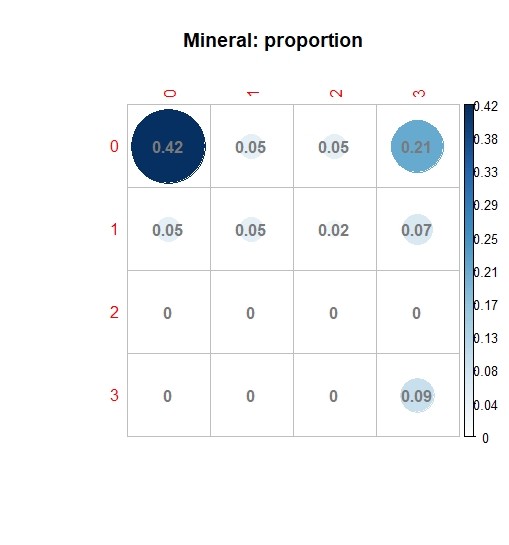
**

0: non-exposed, 1: low, 2: medium, 3: high

**4. References**

1. Hicks, J.; Yager, J. Airborne crystalline silica concentrations at coal-fired power plants associated with coal fly ash. *J Occup Environ Hyg* **2006**, *3*, 448-455.

2. Stacey, P.; Simpson, A.; Hambling, S. The Measurement of Wood in Construction Dust Samples: A Furnace Based Thermal Gravimetric Approach. *Ann Work Expo Health* **2019**, *63*, 1070-1080.

3. Kirkeskov, L.; Hanskov, D.J.; Brauer, C. Total and respirable dust exposures among carpenters and demolition workers during indoor work in Denmark. *J Occup Med Toxicol* **2016**, *11*, 45.

4. Wultsch, G.; Nersesyan, A.; Kundi, M.; Wagner, K.H.; Ferk, F.; Jakse, R.; Knasmueller, S. Impact of exposure to wood dust on genotoxicity and cytotoxicity in exfoliated buccal and nasal cells. *Mutagenesis* **2015**, *30*, 701-709.

5. Bruschweiler, E.D.; Hopf, N.B.; Wild, P.; Huynh, C.K.; Fenech, M.; Thomas, P.; Hor, M.; Charriere, N.; Savova-Bianchi, D.; Danuser, B. Workers exposed to wood dust have an increased micronucleus frequency in nasal and buccal cells: results from a pilot study. *Mutagenesis* **2014**, *29*, 201-207.

6. Martin, J.R.; Zalk, D.M. Comparison of total dust/inhalable dust sampling methods for the evaluation of airborne wood dust. *Applied Occupational and Environmental Hygiene* **1998**, *13*, 177-182.

7. Lintermann, A.; Schröder, W. Simulation of aerosol particle deposition in the upper human tracheobronchial tract. *European Journal of Mechanics - B/Fluids* **2017**, *63*, 73-89.

8. Ricco, M. Lung fibrosis and exposure to wood dusts: Two case reports and review of the literature. *Med Pr* **2015**, *66*, 739-747.

9. Huynh, C.K.; Schüpfer, P.; Boiteux, P. Occupational exposure to Polycyclic Aromatic Hydrocarbons in wood dust. *Journal of Physics: Conference Series* **2009**, *151*.

10. Obata, H.; Dittrick, M.; Chan, H.; Chan-Yeung, M. Occupational asthma due to exposure to African cherry (Makore) wood dust. *Intern Med* **2000**, *39*, 947-949.

11. Stacey, P. A study to assess the performance of an “X-ray powder diffraction with Rietveld” approach for measuring the crystalline and amorphous components of inhalable dust collected on aerosol sampling filters. *Powder Diffraction* **2019**, *34*, 251-259.

12. Estlander, T.; Jolanki, R.; Alanko, K.; Kanerva, L. Occupational allergic contact dermatitis caused by wood dusts. *Contact Dermatitis* **2001**, *44*, 213-217.

13. Lipscomb, H.J.; Dement, J.M. Respiratory diseases among union carpenters: Cohort and case-control analyses. *American Journal of Industrial Medicine* **1998**, *33*, 131-150.

14. Martin, J.R.; Zalk, D.M. Carpenter shop wood dust control: Practical experience to reduce hardwood dust exposures below the American conference of governmental industrial hygienists threshold limit values. *Applied Occupational and Environmental Hygiene* **1997**, *12*, 595-605.

15. Robinson, C.F.; Petersen, M.; Sieber, W.K.; Palu, S.; Halperin, W.E. Mortality of Carpenters' Union members employed in the U.S. construction or wood products industries, 1987–1990. *American Journal of Industrial Medicine* **1996**, *30*, 674-694.

16. Reijula, K.; Kujala, V.; Latvala, J. Sauna builder's asthma caused by obeche (Triplochiton scleroxylon) dust. *Thorax* **1994**, *49*, 622-623.

17. Scherr, P.A.; Hutchison, G.B.; Neiman, R.S. Non-Hodgkin's lymphoma and occupational exposure. *Cancer Res* **1992**, *52*, 5503s-5509s.

18. Godnič-Cvar, J.; Gomzi, M. Case report of occupational asthma due to palisander wood dust and bronchoprovocation challenge by inhalation of pure wood dust from a capsule. *American Journal of Industrial Medicine* **1990**, *18*, 541-545.

19. Kawachi, I.; Pearce, N.; Fraser, J. A New Zealand Cancer Registry-based study of cancer in wood workers. *Cancer* **1989**, *64*, 2609-2613.

20. Hausen, B.M. Contact allergy to woods. *Clinics in Dermatology* **1986**, *4*, 65-76.

21. Blair, A.; Walrath, J.; Rogot, E. Mortality Patterns Among U.S. Veterans by Occupation. I. Cancer. *JNCI: Journal of the National Cancer Institute* **1985**, *75*, 1039-1047.

22. Sabroe, S.; Olsen, J. Health Complaints and Work Conditions among Lacquerers in the Danish Furniture Industry. *Scandinavian Journal of Social Medicine* **1979**, *7*, 97-104.

23. Engzell, U.; Englund, A.; Westerholm, P. Nasal Cancer Associated with Occupational Exposure to Organic Dust. *Acta Oto-Laryngologica* **1978**, *86*, 437-442.

24. Gold, C.; Cuthbert, J. Asbestos — A hazard to the community. *Public Health* **1966**, *80*, 261-270.

25. Mohan, M.; Aprajita; Panwar, N.K. Effect of wood dust on respiratory health status of carpenters. *J Clin Diagn Res* **2013**, *7*, 1589-1591.

26. Aranda, A.; Campo, P.; Palacin, A.; Dona, I.; Gomez-Casado, C.; Galindo, L.; Diaz-Perales, A.; Blanca, M. Antigenic proteins involved in occupational rhinitis and asthma caused by obeche wood (Triplochiton scleroxylon). *PLoS One* **2013**, *8*, e53926.

27. Gomez Yepes, M.E.; Cremades, L.V. Characterization of wood dust from furniture by scanning electron microscopy and energy-dispersive x-ray analysis. *Ind Health* **2011**, *49*, 492-500.

28. Rekhadevi, P.V.; Mahboob, M.; Rahman, M.F.; Grover, P. Genetic damage in wood dust-exposed workers. *Mutagenesis* **2008**, *24*, 59-65.

29. Pesch, B.; Pierl, C.B.; Gebel, M.; Gross, I.; Becker, D.; Johnen, G.; Rihs, H.P.; Donhuijsen, K.; Lepentsiotis, V.; Meier, M., et al. Occupational risks for adenocarcinoma of the nasal cavity and paranasal sinuses in the German wood industry. *Occupational and Environmental Medicine* **2008**, *65*, 191-196.

30. Spee, T.; Hoof, E.V.D.R.V.; Hoof, W.V.; Noy, D.; Kromhout, H. Exposure to wood dust among carpenters in the construction industry in The Netherlands. *Annals of Occupational Hygiene* **2007**, *51*, 241-248.

31. Oliver, L.C.; Miracle-McMahill, H. Airway disease in highway and tunnel construction workers exposed to silica. *Am J Ind Med* **2006**, *49*, 983-996.

32. Kauppinen, T.; Vincent, R.; Liukkonen, T.; Grzebyk, M.; Kauppinen, A.; Welling, I.; Arezes, P.; Black, N.; Bochmann, F.; Campelo, F., et al. Occupational exposure to inhalable wood dust in the member states of the European Union. *Ann Occup Hyg* **2006**, *50*, 549-561.

33. Dement, J.; Pompeii, L.; Lipkus, I.M.; Samsa, G.P. Cancer Incidence Among Union Carpenters in New Jersey. **2003**, *45*, 1059-1067.

34. Zahm, S.H.; Brownson, R.C.; Chang, J.C.; Davis, J.R. Study of lung cancer histologic types, occupation, and smoking in missouri. *American Journal of Industrial Medicine* **1989**, *15*, 565-578.

35. Voss, R.; Stenersen, T.; Oppedal, B.R.; Boysen, M. Sinonasal cancer and exposure to softwood. *Acta Oto-Laryngologica* **1985**, *99*, 172-178.

36. Acheson, E.D.; Cowdell, R.H.; Rang, E.H. Nasal cancer in England and Wales: an occupational survey. *Br J Ind Med* **1981**, *38*, 218-224.

37. Olsen, J.; Sabroe, S. Mortality among Bricklayers and Carpenters/cabinet makers. *Scandinavian Journal of Social Medicine* **1979**, *7*, 49-54.

38. Ironside, P.; Matthews, J. Adenocarcinoma of the nose and paranasal sinuses in woodworkers in the state of Victoria, Australia. *Cancer* **1975**, *36*, 1115-1124.

39. Stellman, S.D.; Garfinkel, L. Cancer mortality among woodworkers. *American Journal of Industrial Medicine* **1984**, *5*, 343-357.

40. Shepherd, S.; Woskie, S.R.; Holcroft, C.; Ellenbecker, M. Reducing silica and dust exposures in construction during use of powered concrete-cutting hand tools: Efficacy of local exhaust ventilation on hammer drills. *Journal of Occupational and Environmental Hygiene* **2008**, *6*, 42-51.

41. Rappaport, S.M.; Goldberg, M.; Susi, P.; Herrick, R.F. Excessive exposure to silica in the US construction industry. *Ann Occup Hyg* **2003**, *47*, 111-122.

42. Flanagan, M.; Seixas, N.; Majar, M.; Camp, J.; Morgan, M. Silica dust exposures during selected construction activities. *American Industrial Hygiene Association Journal* **2003**, *64*, 319-328.

43. Blute, N.A.; Woskie, S.R.; Greenspan, C.A. Exposure characterization for highway construction part I: Cut and cover and tunnel finish stages. *Applied Occupational and Environmental Hygiene* **1999**, *14*, 632-641.

44. Greenspan, C.A.; Moure-Eraso, A.R.; Wegman, D.H.; Olivep, L.C. Occupational hygiene characterization of a highway construction project: A pilot study. *Applied Occupational and Environmental Hygiene* **1995**, *10*, 50-58.

45. Peters, S.; Thomassen, Y.; Fechter-Rink, E.; Kromhout, H. Personal exposure to inhalable cement dust among construction workers. *J. Environ. Monit.* **2009**, *11*, 174-180.

46. Carlo, R.V.; Sheehy, J.; Amy Feng, H.; Sieber, W.K. Laboratory evaluation to reduce respirable crystalline silica dust when cutting concrete roofing tiles using a masonry saw. *Journal of Occupational and Environmental Hygiene* **2010**, *7*, 245-251.

47. Meijer, E.; Tjoe Nij, E.; Kraus, T.; van der Zee, J.S.; van Delden, O.; van Leeuwen, M.; Lammers, J.W.; Heederik, D. Pneumoconiosis and emphysema in construction workers: results of HRCT and lung function findings. *Occupational and Environmental Medicine* **2011**, *68*, 542.

48. Toren, K.; Qvarfordt, I.; Bergdahl, I.A.; Jarvholm, B. Increased mortality from infectious pneumonia after occupational exposure to inorganic dust, metal fumes and chemicals. *Thorax* **2011**, *66*, 992-996.

49. Calvert, G.M.; Luckhaupt, S.E.; Sussell, A.; Dahlhamer, J.M.; Ward, B.W. The prevalence of selected potentially hazardous workplace exposures in the US: findings from the 2010 National Health Interview Survey. *Am J Ind Med* **2013**, *56*, 635-646.

50. Beaudry, C.; Lavoue, J.; Sauve, J.F.; Begin, D.; Senhaji Rhazi, M.; Perrault, G.; Dion, C.; Gerin, M. Occupational exposure to silica in construction workers: a literature-based exposure database. *J Occup Environ Hyg* **2013**, *10*, 71-77.

51. Carino, M.; Romita, P.; Foti, C. Allergy-related disorders in the construction industry. *ISRN Prev Med* **2013**, *2013*, 864679.

52. Torén, K.; Järvholm, B. Effect of Occupational Exposure to Vapors, Gases, Dusts, and Fumes on COPD Mortality Risk Among Swedish Construction Workers: A Longitudinal Cohort Study. *CHEST* **2014**, *145*, 992-997.

53. van Deurssen, E.; Pronk, A.; Spaan, S.; Goede, H.; Tielemans, E.; Heederik, D.; Meijster, T. Quartz and respirable dust in the Dutch construction industry: a baseline exposure assessment as part of a multidimensional intervention approach. *Ann Occup Hyg* **2014**, *58*, 724-738.

54. Bakke, B.; Ulvestad, B.; Thomassen, Y.; Woldbaek, T.; Ellingsen, D.G. Characterization of occupational exposure to air contaminants in modern tunnelling operations. *Ann Occup Hyg* **2014**, *58*, 818-829.

55. Oude Hengel, K.M.; van Deurssen, E.; Meijster, T.; Tielemans, E.; Heederik, D.; Pronk, A. 'Relieved Working' study: systematic development and design of an intervention to decrease occupational quartz exposure at construction worksites. *BMC Public Health* **2014**, *14*, 760.

56. Ringen, K.; Dement, J.; Welch, L.; Dong, X.S.; Bingham, E.; Quinn, P.S. Risks of a lifetime in construction. Part II: Chronic occupational diseases. *Am J Ind Med* **2014**, *57*, 1235-1245.

57. Ulvestad, B.; Lund, M.B.; Bakke, B.; Thomassen, Y.; Ellingsen, D.G. Short-term lung function decline in tunnel construction workers. *Occupational and Environmental Medicine* **2015**, *72*, 108-113.

58. Ellingsen, D.G.; Ulvestad, B.; Bakke, B.; Seljeflot, I.; Barregard, L.; Thomassen, Y. Serum pneumoproteins in tunnel construction workers. *Int Arch Occup Environ Health* **2015**, *88*, 943-951.

59. Blanc, P.D.; Järvholm, B.; Torén, K. Prospective risk of rheumatologic disease associated with occupational exposure in a cohort of male construction workers. *American Journal of Medicine* **2015**, *128*, 1094-1101.

60. Lacourt, A.; Pintos, J.; Lavoué, J.; Richardson, L.; Siemiatycki, J. Lung cancer risk among workers in the construction industry: results from two case-control studies in Montreal. *BMC public health* **2015**, *15*, 941-941.

61. Galea, K.S.; Mair, C.; Alexander, C.; de Vocht, F.; van Tongeren, M. Occupational Exposure to Respirable Dust, Respirable Crystalline Silica and Diesel Engine Exhaust Emissions in the London Tunnelling Environment. *Ann Occup Hyg* **2016**, *60*, 263-269.

62. Ellingsen, D.G.; Seljeflot, I.; Thomassen, Y.; Thomassen, M.; Bakke, B.; Ulvestad, B. Biomarkers of endothelial activation and thrombosis in tunnel construction workers exposed to airborne contaminants. *Int Arch Occup Environ Health* **2017**, *90*, 309-317.

63. Borup, H.; Kirkeskov, L.; Hanskov, D.J.A.; Brauer, C. Systematic review: chronic obstructive pulmonary disease and construction workers. *Occup Med (Lond)* **2017**, *67*, 199-204.

64. Dement, J.M.; Welch, L.S.; Ringen, K.; Cranford, K.; Quinn, P. Longitudinal decline in lung function among older construction workers. *Occupational and Environmental Medicine* **2017**, *74*, 701.

65. McLean, D.; Glass, B.; ’t Mannetje, A.; Douwes, J. Exposure to respirable crystalline silica in the construction industry—do we have a problem? *New Zealand Medical Journal* **2017**, *130*, 78-82.

66. Adhikari, A.; Mitra, A.; Rashidi, A.; Ekpo, I.; Schwartz, J.; Doehling, J. Field Evaluation of N95 Filtering Facepiece Respirators on Construction Jobsites for Protection against Airborne Ultrafine Particles. *Int J Environ Res Public Health* **2018**, *15*.

67. Silva, I.R.; Ramos, M.; Arantes, L.; Lengert, A.V.H.; Oliveira, M.A.; Cury, F.P.; Martins Pereira, G.; Santos, A.G.; Barbosa, F., Jr.; Vasconcellos, P.C., et al. Evaluation of DNA Methylation Changes and Micronuclei in Workers Exposed to a Construction Environment. *Int J Environ Res Public Health* **2019**, *16*.

68. Jonsson, E.; Jarvholm, B.; Andersson, M. Silica dust and sarcoidosis in Swedish construction workers. *Occup Med (Lond)* **2019**, 10.1093/occmed/kqz118.

69. Tjoe Nij, E.; Hilhorst, S.; Spee, T.; Spierings, J.; Steffens, F.; Lumens, M.; Heederik, D. Dust control measures in the construction industry. *Ann Occup Hyg* **2003**, *47*, 211-218.

70. Si, S.; Carey, R.N.; Reid, A.; Driscoll, T.; Glass, D.C.; Peters, S.; Benke, G.; Darcey, E.; Fritschi, L. The Australian Work Exposures Study: Prevalence of Occupational Exposure to Respirable Crystalline Silica. *Ann Occup Hyg* **2016**, *60*, 631-637.

71. Jansson, C.; Johansson, A.L.; Bergdahl, I.A.; Dickman, P.W.; Plato, N.; Adami, J.; Boffetta, P.; Lagergren, J. Occupational exposures and risk of esophageal and gastric cardia cancers among male Swedish construction workers. *Cancer Causes Control* **2005**, *16*, 755-764.

72. Sjodahl, K.; Jansson, C.; Bergdahl, I.A.; Adami, J.; Boffetta, P.; Lagergren, J. Airborne exposures and risk of gastric cancer: a prospective cohort study. *Int J Cancer* **2007**, *120*, 2013-2018.

73. Toren, K.; Bergdahl, I.A.; Nilsson, T.; Jarvholm, B. Occupational exposure to particulate air pollution and mortality due to ischaemic heart disease and cerebrovascular disease. *Occup Environ Med* **2007**, *64*, 515-519.

74. Suarthana, E.; Moons, K.G.; Heederik, D.; Meijer, E. A simple diagnostic model for ruling out pneumoconiosis among construction workers. *Occup Environ Med* **2007**, *64*, 595-601.

75. Sauni, R.; Oksa, P.; Vattulainen, K.; Uitti, J.; Palmroos, P.; Roto, P. The effects of asthma on the quality of life and employment of construction workers. *Occup Med (Lond)* **2001**, *51*, 163-167.

76. Ulvestad, B.; Bakke, B.; Eduard, W.; Kongerud, J.; Lund, M.B. Cumulative exposure to dust causes accelerated decline in lung function in tunnel workers. *Occupational and Environmental Medicine* **2001**, *58*, 663.

77. Ulvestad, B.; Lund, M.B.; Bakke, B.; Djupesland, P.G.; Kongerud, J.; Boe, J. Gas and dust exposure in underground construction is associated with signs of airway inflammation. *European Respiratory Journal* **2001**, *17*, 416.

78. Hilt, B.; Qvenild, T.; Holme, J.; Svendsen, K.; Ulvestad, B. Increase in interleukin-6 and fibrinogen after exposure to dust in tunnel construction workers. *Occupational and environmental medicine* **2002**, *59*, 9-12.

79. Tjoe-Nij, E.; de Meer, G.; Smit, J.; Heederik, D. Lung function decrease in relation to pneumoconiosis and exposure to quartz-containing dust in construction workers. *Am J Ind Med* **2003**, *43*, 574-583.

80. Tjoe Nij, E.; Burdorf, A.; Parker, J.; Attfield, M.; van Duivenbooden, C.; Heederik, D. Radiographic abnormalities among construction workers exposed to quartz containing dust. *Occupational and environmental medicine* **2003**, *60*, 410-417.

81. Bakke, B.; Ulvestad, B.; Stewart, P.; Eduard, W. Cumulative exposure to dust and gases as determinants of lung function decline in tunnel construction workers. *Occup Environ Med* **2004**, *61*, 262-269.

82. Bergdahl, I.A.; Torén, K.; Eriksson, K.; Hedlund, U.; Nilsson, T.; Flodin, R.; Järvholm, B. Increased mortality in COPD among construction workers exposed to inorganic dust. *European Respiratory Journal* **2004**, *23*, 402-406.

83. Rahman, S.A.A.; Yatim, S.R.M.; Abdullah, A.H.; Zainuddin, N.A.; Samah, M.A.A. Exposure of particulate matter 2.5 (PM2.5) on lung function performance of construction workers.

84. Guerrera, E.; Dominici, L.; Levorato, S.; Vannini, S.; Acito, M.; Fatigoni, C.; Villarini, M.; Moretti, M. Cytotoxicity and genotoxicity of size-fractionated particulate matter collected in underground workplaces. *Air Quality, Atmosphere and Health* **2019**, *12*, 359-367.

85. Sąkol, G.; Muszyńska - Graca, M. Air Pollution during Asbestos Removal. *Pol. J. Environ. Stud.* **2019**, *28*, 1007-1011.

86. Montadka, N.; Arocho, I. Methodology to Measure Real-Time PM 2.5 Levels in Equipment Cabins. *Construction Research Congress 2018* doi:10.1061/9780784481301.026

10.1061/9780784481301.026, 257-268.

87. Cooksey, G.L.S.; Wilken, J.A.; McNary, J.; Gilliss, D.; Shusterman, D.; Materna, B.L.; Vugia, D.J. Dust exposure and coccidioidomycosis prevention among solar power farm construction workers in California. *American Journal of Public Health* **2017**, *107*, 1296-1303.

88. Andersson, M.; Selin, F.; Järvholm, B. Asbestos exposure and the risk of sinonasal cancer. *Occupational Medicine* **2016**, *66*, 326-331.

89. Dement, J.; Welch, L.; Ringen, K.; Quinn, P.; Chen, A.; Haas, S. A case-control study of airways obstruction among construction workers. *Am J Ind Med* **2015**, *58*, 1083-1097.

90. Danilova, M.; Stoleski, S.; Mijakoski, D. Respiratory Symptoms and Ventilatory Function in Never-Smoking Males Working in Dusty Occupations. *Open Access Macedonian Journal of Medical Sciences* **2014**, *2*.

91. Timmerman, J.G.; Heederik, D.; Spee, T.; Smit, L.A.M. Skin symptoms in the construction industry: Occurrence and determinants. *American Journal of Industrial Medicine* **2014**, *57*, 660-668.

92. Dominici, L.; Guerrera, E.; Villarini, M.; Fatigoni, C.; Moretti, M.; Blasi, P.; Monarca, S. Evaluation of in vitro cytoxicity and genotoxicity of size-fractionated air particles sampled during road tunnel construction. *BioMed Research International* **2013**, *2013*.

93. Englund, A. Cancer risks among construction workers. *European Journal of Oncology* **2012**, *17*, 5-9.

94. Yeheyis, M.; Aguilar, G.; Hewage, K.; Sadiq, R. Exposure to Crystalline Silica Inhalation Among Construction Workers: A Probabilistic Risk Analysis. *Human and Ecological Risk Assessment: An International Journal* **2012**, *18*, 1036-1050.

95. Hinze, J.; Giang, G. Factors associated with construction worker eye injuries. *Safety Science* **2008**, *46*, 634-645.

96. Buratti, M.; Campo, L.; Fustinoni, S.; Valla, C.; Martinotti, I.; Cirla, P.E.; Cavallo, D.; Foà, V. Application of ultraviolet spectrophotometry to estimate occupational exposure to airborne polyaromatic compounds in asphalt pavers. *Journal of Occupational and Environmental Hygiene* **2007**, *4*, 412-419.

97. Purdue, M.P.; Järvholm, B.; Bergdahl, I.A.; Hayes, R.B.; Baris, D. Occupational exposures and head and neck cancers among Swedish construction workers. *Scandinavian Journal of Work, Environment and Health* **2006**, *32*, 270-275.

98. JÄRvholm, B. Carcinogens in the Construction Industry. *Annals of the New York Academy of Sciences* **2006**, *1076*, 421-428.

99. Tjoe Nij, E.; Höhr, D.; Borm, P.; Burstyn, I.; Spierings, J.; Steffens, F.; Lumens, M.; Spee, T.; Heederik, D. Variability in Quartz Exposure in the Construction Industry: Implications for Assessing Exposure-Response Relations. *Journal of Occupational and Environmental Hygiene* **2004**, *1*, 191-198.

100. Arcangeli, G.; Cupelli, V.; Montalti, M.; Pristera, M.; Baldasseroni, A.; Giuliano, G. Respiratory risks in tunnel construction workers. *Int J Immunopathol Pharmacol* **2004**, *17*, 91-96.

101. Verma, D.K.; Kurtz, L.A.; Sahai, D.; Finkelstein, M.M. Current chemical exposures among ontario construction workers. *Applied Occupational and Environmental Hygiene* **2003**, *18*, 1031-1047.

102. Sauni, R.; Oksa, P.; Huikko, S.; Roto, P.; Uitti, J. Increased risk of asthma among Finnish construction workers. *Occupational Medicine* **2003**, *53*, 527-531.

103. Lee, W.J.; Baris, D.; Järvholm, B.; Silverman, D.T.; Bergdahl, I.A.; Blair, A. Multiple myeloma and diesel and other occupational exposures in Swedish construction workers. *International Journal of Cancer* **2003**, *107*, 134-138.

104. Kauppinen, T.; Heikkilä, P.; Partanen, T.; Virtanen, S.V.; Pukkala, E.; Ylöstalo, P.; Burstyn, I.; Ferro, G.; Boffetta, P. Mortality and cancer incidence of workers in Finnish road paving companies. *American Journal of Industrial Medicine* **2003**, *43*, 49-57.

105. Bakke, B.; Stewart, P.; Eduard, W. Determinants of dust exposure in tunnel construction work. *Applied Occupational and Environmental Hygiene* **2002**, *17*, 783-796.

106. Nij, E.T.; Borm, P.; Höhr, D.; Heederik, D. Pneumoconiosis and exposure to quartz-containing dust in the construction industry. *Annals of Occupational Hygiene* **2002**, *46*, 71-75.

107. Woskie, S.R.; Kalil, A.; Bello, D.; Abbasvirji, M. Exposures to quartz, diesel, dust, and welding fumes during heavy and highway construction. *American Industrial Hygiene Association Journal* **2002**, *63*, 447-457.

108. Bakke, B.; Stewart, P.; Ulvestad, B.; Eduard, W. Dust and gas exposure in tunnel construction work. *American Industrial Hygiene Association Journal* **2001**, *62*, 457-465.

109. Petersen, J.S.; Zwerling, C. Comparison of health outcomes among older construction and blue-collar employees in the United States. *American Journal of Industrial Medicine* **1998**, *34*, 280-287.

110. Epling, C.A.; Rose, C.S.; Martyny, J.W.; Zhen, B.; Alexander, W.; Waldron Jr, J.A.; Kreiss, K. Endemic work-related febrile respiratory illness among construction workers. *American Journal of Industrial Medicine* **1995**, *28*, 193-205.

111. Oksa, P.; Koskinen, H.; Rinne, J.P.; Zitting, A.; Roto, P.; Huuskonen, M.S. Parenchymal and pleural fibrosis in construction workers. *American Journal of Industrial Medicine* **1992**, *21*, 561-567.

112. Gonzalez, C.A.; Sanz, M.; Marcos, G.; Pita, S.; Brullet, E.; Vida, F.; Agudo, A.; Hsieh, C.C. Occupation and gastric cancer in Spain. *Scand J Work Environ Health* **1991**, *17*, 240-247.

113. Heederik, D.; Kromhout, H.; Burema, J.A.N.; Biersteker, K.; Kromhout, D. Occupational Exposure and 25-Year Incidence Rate of Non-Specific Lung Disease: The Zutphen Study. *International Journal of Epidemiology* **1990**, *19*, 945-952.

114. Roush, G.C.; Meigs, J.W.; Kelly, J.; Flannery, J.T.; Burdo, H. SINONASAL CANCER AND OCCUPATION: A CASE-CONTROL STUDY1. *American Journal of Epidemiology* **1980**, *111*, 183-193.

115. Askergren, A.; Szamosi, A. Relation between radiological pleuropulmonary changes, clinical history and weight index of construction workers. *Scandinavian Journal of Work, Environment & Health* **1978**, 10.5271/sjweh.2710, 179-183.

116. Fischbein, A.; Luo, J.C.J.; Lacher, M.; Rosenfeld, S.; Rosenbaum, A.; Miller, A.; Solomon, S.J. Respiratory Findings Among Millwright and Machinery Erectors: Identification of Health Hazards from Asbestos in Place at Work. *Environmental Research* **1993**, *61*, 25-35.

117. Finkelstein, M.M. Diesel particulate exposure and diabetes mortality among workers in the Ontario construction trades. *Occupational and Environmental Medicine* **2008**, *65*, 215.

118. Fitzgerald, E.F.; Stark, A.D.; Hwang, S.A.; Vianna, N. Exposure to asbestiform minerals and radiographic chest abnormalities in a talc mining region of upstate new york. *Archives of Environmental Health* **1991**, *46*, 151-154.

119. Pan, S.Y.; Ugnat, A.-M.; Mao, Y.; Group, T.C.C.R.E.R. Occupational Risk Factors for Brain Cancer in Canada. **2005**, *47*, 704-717.

120. Thériault, G.P.; Tremblay, C.G.; Armstrong, B.G. Risk of ischemic heart disease among primary aluminum production workers. *American Journal of Industrial Medicine* **1988**, *13*, 659-666.

121. Stebbins, A.I.; Horstman, S.W.; Daniell, W.E.; Atallah, R. COBALT EXPOSURE IN A CARBIDE TIP GRINDING PROCESS. *American Industrial Hygiene Association Journal* **1992**, *53*, 186-192.

122. Armstead, A.L.; Li, B. Nanotoxicity: emerging concerns regarding nanomaterial safety and occupational hard metal (WC-Co) nanoparticle exposure. *Int J Nanomedicine* **2016**, *11*, 6421-6433.

123. Klasson, M.; Bryngelsson, I.L.; Pettersson, C.; Husby, B.; Arvidsson, H.; Westberg, H. Occupational Exposure to Cobalt and Tungsten in the Swedish Hard Metal Industry: Air Concentrations of Particle Mass, Number, and Surface Area. *Ann Occup Hyg* **2016**, *60*, 684-699.

124. Park, R.M.; Baldwin, M.; Bouchard, M.F.; Mergler, D. Airborne manganese as dust vs. fume determining blood levels in workers at a manganese alloy production plant. *Neurotoxicology* **2014**, *45*, 267-275.

125. Park, R.M.; Bouchard, M.F.; Baldwin, M.; Bowler, R.; Mergler, D. Respiratory manganese particle size, time-course and neurobehavioral outcomes in workers at a manganese alloy production plant. *Neurotoxicology* **2014**, *45*, 276-284.

126. Hughson, G.W.; Galea, K.S.; Heim, K.E. Characterization and assessment of dermal and inhalable nickel exposures in nickel production and primary user industries. *Ann Occup Hyg* **2010**, *54*, 8-22.

127. Johnsen, H.L.; Hetland, S.M.; Saltyte Benth, J.; Kongerud, J.; Soyseth, V. Quantitative and qualitative assessment of exposure among employees in Norwegian smelters. *Ann Occup Hyg* **2008**, *52*, 623-633.

128. Tsai, P.-J.; Vincent, J.H. A study of workers' exposures to the inhalable and ‘Total’ aerosol fractions in the primary nickel production industry using mannequins to simulate personal sampling. *The Annals of Occupational Hygiene* **2001**, *45*, 385-394.

129. Gibb, H.J.; Lees, P.S.J.; Pinsky, P.F.; Rooney, B.C. Lung cancer among workers in chromium chemical production. *American Journal of Industrial Medicine* **2000**, *38*, 115-126.

130. Lison, D. Human Toxicity of Cobalt-Containing Dust and Experimental Studies on the Mechanism of Interstitial Lung Disease (Hard Metal Disease). *Critical Reviews in Toxicology* **1996**, *26*, 585-616.

131. Mergler, D.; Huel, G.; Bowler, R.; Iregren, A.; Belanger, S.; Baldwin, M.; Tardif, R.; Smargiassi, A.; Martin, L. Nervous System Dysfunction among Workers with Long-Term Exposure to Manganese. *Environmental Research* **1994**, *64*, 151-180.

132. D'Arcy, J.B.; Dasch, J.M.; Gundrum, A.B.; Rivera, J.L.; Johnson, J.H.; Carlson, D.H.; Sutherland, J.W. Characterization of process air emissions in automotive production plants. *J Occup Environ Hyg* **2016**, *13*, 9-18.

133. Baxter, P.J.; McDowall, M.E. Occupation and cancer in London: an investigation into nasal and bladder cancer using the Cancer Atlas. *British journal of industrial medicine* **1986**, *43*, 44-49.

134. Loupa, G. Case Study. *Journal of Occupational and Environmental Hygiene* **2013**, *10*, D135-D146.

135. Paustenbach, D.J.; Richter, R.O.; Finley, B.L.; Sheehan, P.J. An Evaluation of the Historical Exposures of Mechanics to Asbestos in Brake Dust. *Applied Occupational and Environmental Hygiene* **2003**, *18*, 786-804.

136. Rödelsperger, K.; Jahn, H.; Brückel, B.; Manke, J.; Paur, R.; Woitowitz Med, H.J.P.D. Asbestos dust exposure during brake repair. *American Journal of Industrial Medicine* **1986**, *10*, 63-72.

137. Wolkoff, P.; Schneider, T.; Kildesø, J.; Degerth, R.; Jaroszewski, M.; Schunk, H. Risk in cleaning: chemical and physical exposure. *Science of The Total Environment* **1998**, *215*, 135-156.

138. Riala, R. Dust and quartz exposure of Finnish construction site cleaners. *Ann Occup Hyg* **1988**, *32*, 215-220.

139. Sjögren, B.; Fredlund, P.; Lundberg, I.; Weiner, J. Ischemic heart disease in female cleaners. *International Journal of Occupational and Environmental Health* **2003**, *9*, 134-137.

140. Donham, K.; Haglind, P.; Peterson, Y.; Rylander, R.; Belin, L. Environmental and health studies of farm workers in Swedish swine confinement buildings. *British journal of industrial medicine* **1989**, *46*, 31-37.

141. Crook, B.; Robertson, J.F.; Glass, S.A.; Botheroyd, E.M.; Lacey, J.; Topping, M.D. Airborne dust, ammonia, microorganisms, and antigens in pig confinement houses and the respiratory health of exposed farm workers. *Am Ind Hyg Assoc J* **1991**, *52*, 271-279.

142. Muscat, J.E.; Stellman, S.D.; Richie, J.P.; Wynder, E.L. Lung Cancer Risk and Workplace Exposures in Black Men and Women. *Environmental Research* **1998**, *76*, 78-84.

143. Basinas, I.; Sigsgaard, T.; Erlandsen, M.; Andersen, N.T.; Takai, H.; Heederik, D.; Omland, O.; Kromhout, H.; Schlunssen, V. Exposure-affecting factors of dairy farmers' exposure to inhalable dust and endotoxin. *Ann Occup Hyg* **2014**, *58*, 707-723.

144. Basinas, I.; Cronin, G.; Hogan, V.; Sigsgaard, T.; Hayes, J.; Coggins, A.M. Exposure to inhalable dust, endotoxin, and total volatile organic carbons on dairy farms using manual and automated feeding systems. *Annals of Work Exposures and Health* **2017**, *61*, 344-355.

145. Reeb-Whitaker, C.K.; Bonauto, D.K. Respiratory disease associated with occupational inhalation to hop (Humulus lupulus) during harvest and processing. *Annals of Allergy, Asthma and Immunology* **2014**, *113*, 534-538.

146. Reynolds, S.J.; Clark, M.L.; Koehncke, N.; Von Essen, S.G.; Prinz, L.; Keefe, T.J.; Mehaffy, J.; Bradford, M.; Cranmer, B.; Davidson, M.E., et al. Pulmonary function reductions among potentially susceptible subgroups of agricultural workers in Colorado and Nebraska. *Journal of Occupational and Environmental Medicine* **2012**, *54*, 632-641.

147. Schenker, M. Exposures and health effects from inorganic agricultural dusts. *Environ Health Perspect* **2000**, *108 Suppl 4*, 661-664.

148. Parks, C.G.; Cooper, G.S.; Nylander-French, L.A.; Storm, J.F.; Archer, J.D. Assessing Exposure to Crystalline Silica from Farm Work: A Population-based Study in the Southeastern United States. *Annals of Epidemiology* **2003**, *13*, 385-392.

149. Mitloehner, F.M.; Calvo, M.S. Worker health and safety in concentrated animal feeding operations. *J Agric Saf Health* **2008**, *14*, 163-187.

150. Mills, P.K.; Dodge, J.; Yang, R. Cancer in migrant and seasonal hired farm workers. *J Agromedicine* **2009**, *14*, 185-191.

151. Swanberg, J.E.; Clouser, J.M.; Gan, W.; Mannino, D.M.; Flunker, J.C. Individual and occupational characteristics associated with respiratory symptoms among Latino horse farm workers. *Am J Ind Med* **2015**, *58*, 679-687.

152. Ngajilo, D.; Singh, T.; Ratshikhopha, E.; Dayal, P.; Matuka, O.; Baatjies, R.; Jeebhay, M.F. Risk factors associated with allergic sensitization and asthma phenotypes among poultry farm workers. *Am J Ind Med* **2018**, *61*, 515-523.

153. Viegas, S.; Mateus, V.; Almeida-Silva, M.; Carolino, E.; Viegas, C. Occupational exposure to particulate matter and respiratory symptoms in Portuguese swine barn workers. *J Toxicol Environ Health A* **2013**, *76*, 1007-1014.

154. Mc Donnell, P.E.; Coggins, M.A.; Hogan, V.J.; Fleming, G.T. Exposure assessment of airborne contaminants in the indoor environment of Irish swine farms. *Ann Agric Environ Med* **2008**, *15*, 323-326.

155. Cho, K.J.; Jones, S.; Jones, G.; McKay, R.; Grinshpun, S.A.; Dwivedi, A.; Shukla, R.; Singh, U.; Reponen, T. Effect of Particle Size on Respiratory Protection Provided by Two Types of N95 Respirators Used in Agricultural Settings. *Journal of Occupational and Environmental Hygiene* **2010**, *7*, 622-627.

156. Rodriquez, E.J.; Stoecklin-Marois, M.T.; Bennett, D.H.; Tancredi, D.J.; Schenker, M.B. Agricultural Work Exposures and Pulmonary Function Among Hired Farm Workers in California (The MICASA Study). *Journal of Agromedicine* **2014**, *19*, 427-436.

157. Stoecklin-Marois, M.T.; Bigham, C.W.; Bennett, D.H.; Tancredi, D.J.; Schenker, M.B. Occupational Exposures and Migration Factors Associated With Respiratory Health in California Latino Farm Workers. *The MICASA Study* **2015**, *57*, 152-158.

158. Gilbey, S.E.; Selvey, L.A.; Mead-Hunter, R.; Mullins, B.; Netto, K.; Zhao, Y.; Rumchev, K.B. Occupational exposures to agricultural dust by Western Australian wheat-belt farmers during seeding operations. *J Occup Environ Hyg* **2018**, *15*, 824-832.

159. Conroy, L.M.; Lindsay, R.M.; Sullivan, P.M. Lead, chromium, and cadmium emission factors during abrasive blasting operations by bridge painters. *Am Ind Hyg Assoc J* **1995**, *56*, 266-271.

160. Madhavi, D.; Devi, K.R.; Sowjanya, B.L. Increased frequency of chromosomal aberrations in industrial painters exposed to lead-based paints. *J Environ Pathol Toxicol Oncol* **2008**, *27*, 53-59.

161. Scholz, P.F.; Materna, B.L.; Harrington, D.; Uratsu, C. Residential and Commercial Painters' Exposure to Lead during Surface Preparation. *AIHA Journal* **2002**, *63*, 22-28.

162. Ennever, F.K.; Zaccaro, D.J.; Fernando, R.A.; Jones, B.T. Blood lead levels in North Carolina painters. *Human & Experimental Toxicology* **1995**, *14*, 456-461.

163. Nored, A.W.; Chalbot, M.-C.G.; Kavouras, I.G. Characterization of paint dust aerosol generated from mechanical abrasion of TiO2-containing paints. *Journal of Occupational and Environmental Hygiene* **2018**, *15*, 629-640.

164. Norbäck, D.; Wieslander, G.; Edling, C. Occupational exposure to volatile organic compounds (VOCs), and other air pollutants from the indoor application of water-based paints. *The Annals of Occupational Hygiene* **1995**, *39*, 783-794.

165. Hellquist, H.; Irander, K.; Edling, C.; Ödkvist, L.M. Nasal Symptoms and Histopathology in A Group of Spray-Painters. *Acta Oto-Laryngologica* **1983**, *96*, 495-500.

166. Schyllert, C.; Andersson, M.; Hedman, L.; Ekstrom, M.; Backman, H.; Lindberg, A.; Ronmark, E. Job titles classified into socioeconomic and occupational groups identify subjects with increased risk for respiratory symptoms independent of occupational exposure to vapour, gas, dust, or fumes. *Eur Clin Respir J* **2018**, *5*, 1468715.

167. Schubert, S.; Bauer, A.; Molin, S.; Skudlik, C.; Geier, J. Occupational contact sensitization in female geriatric nurses: Data of the Information Network of Departments of Dermatology (IVDK) 2005-2014. *J Eur Acad Dermatol Venereol* **2017**, *31*, 469-476.

168. Jensen, P.; Menne, T.; Thyssen, J.P. Allergic contact dermatitis in a nurse caused by airborne rubber additives. *Contact Dermatitis* **2011**, *65*, 54-55.

169. Liss, G.M.; Buyantseva, L.; Luce, C.E.; Ribeiro, M.; Manno, M.; Tarlo, S.M. Work-related asthma in health care in Ontario. *American Journal of Industrial Medicine* **2011**, *54*, 278-284.

170. Ballbè, M.; Sureda, X.; Martínez-Sánchez, J.M.; Fu, M.; Saltó, E.; Gual, A.; Fernández, E. Secondhand smoke in psychiatric units: patient and staff misperceptions. *Tobacco Control* **2015**, *24*, e212.

171. Machado, L.; Olsson, G.; Stålenheim, G.; Zetterström, O. Dust Exposure Challenge Test as a Measure of Potential Allergenicity and Occupational Disease Risk in Handling of Ispaghula Products. *Allergy* **1983**, *38*, 141-144.

172. Machado, L.; Zetterström, O.; Fagerberg, E. Occupational Allergy in Nurses to a Bulk Laxative. *Allergy* **1979**, *34*, 51-55.

173. A. Abakay, S.A., O. Abakay, Y. Atalay, S. Güven, F. Yaman, Y. Palanci, G. Tekbas, A. Dalli, A.C. Tanrikulu. Frequency of respiratory function disorders among dental laboratory technicians working under conditions of high dust concentration. *Eur Rev Med Pharmacol Sci 2013* **2013**.

174. Bozkurt, N.; Yurdasal, B.; Bozkurt, A.I.; Yilmaz, O.; Tekin, M. Respiratory Systems of Dental Technicians Negatively Affected during 5 Years of Follow-Up. *Balkan Med J* **2016**, *33*, 426-433.

175. Yurdasal, B.; Bozkurt, N.; Bozkurt, A.İ.; Yilmaz, Ö. The evaluation of the dust-related occupational respiratory disorders of dental laboratory technicians working in Denizli Province. *Ann Thorac Med* **2015**, *10*, 249-255.

176. Hu, S.W.; Lin, Y.Y.; Wu, T.C.; Hong, C.C.; Chan, C.C.; Lung, S.C. Workplace air quality and lung function among dental laboratory technicians. *Am J Ind Med* **2006**, *49*, 85-92.

177. Lijewski, P.; Merkisz, J.; Fuc, P. The Analysis of the Emission of Particulate Matter from Non-Road Vehicles Under Actual Operating Conditions.

178. Nayebzadeh, A.; Dufresne, A.; Harvie, S.; Bégin, R. Mineralogy of Lung Tissue in Dental Laboratory Technicians' Pneumoconiosis. *American Industrial Hygiene Association Journal* **1999**, *60*, 349-353.

179. Brancaleone, P.; Weynnand, B.; De Vuyst, P.; Stanestcu, D.; Pieters, T. Lung granulomatosis in a dental technician. *Pneumologie* **1999**, *53*, M72-M73.

180. Jacobsen, N.; Derand, T.; Hensten-Pettersen, A. Profile of work-related health complaints among Swedish dental laboratory technicians. *Community Dentistry and Oral Epidemiology* **1996**, *24*, 138-144.

181. Muresanu, D.F.; Sharma, A.; Patnaik, R.; Nozar, A.; Mossier, H.; Lafuente, J.V.; Tian, Z.R.; Ozikzilcik, A.; Sharma, H.S. Nanodelivery of cerebrolysin induces profound neuroprotection in heat stroke following chronic hypertension in combination with Si02 and carbon nanoparticles induced exacerbation of brain damage. pp. 29-32.

182. Sharma, H.S.; Muresanu, D.F.; Lafuente, J.V.; Patnaik, R.; Tian, Z.R.; Mos̈sler, H.; Sharma, A. TiO2-nanowired cerebrolysin attenuated hyperthermia induced ubiquitin overexpression and brain pathology. pp. 303-306.

183. Tarvainen, L.; Kyyrönen, P.; Kauppinen, T.; Pukkala, E. Cancer of the mouth and pharynx, occupation and exposure to chemical agents in Finland [in 1971–95]. *International Journal of Cancer* **2008**, *123*, 653-659.

184. Susi, P.; Goldberg, M.; Barnes, P.; Stafford, E. The Use of a Task-Based Exposure Assessment Model (T-BEAM) for Assessment of Metal Fume Exposures During Welding and Thermal Cutting. *Applied Occupational and Environmental Hygiene* **2000**, *15*, 26-38.

185. Chinn, D.J.; Stevenson, I.C.; Cotes, J.E. Longitudinal respiratory survey of shipyard workers: effects of trade and atopic status. *British Journal of Industrial Medicine* **1990**, *47*, 83.

186. Stern, R.M. Cancer incidence among welders: possible effects of exposure to extremely low frequency electromagnetic radiation (ELF) and to welding fumes. *Environmental Health Perspectives* **1987**, *76*, 221-229.

187. Horsfield, K.; Cooper, F.M.; Buckman, M.P.; Guyatt, A.R.; Cumming, G. Respiratory symptoms in West Sussex firemen. *Br J Ind Med* **1988**, *45*, 251-255.

188. Genovesi, M.G.; Tashkin, D.P.; Chopra, S.; Morgan, M.; McElroy, C. Transient hypoxemia in firemen following inhalation of smoke. *Chest* **1977**, *71*, 441-444.

189. Shiels, D.O.; Robertson, I. Poisoning of firemen by irritant fumes; cadmium and sulphonated castor oil. *British journal of industrial medicine* **1946**, *3*, 213-224.

190. Axford, A.T.; McKerrow, C.B.; Jones, A.P.; Le Quesne, P.M. Accidental exposure to isocyanate fumes in a group of firemen. *British Journal of Industrial Medicine* **1976**, *33*, 65-71.

191. Fine, J.M.; Gordon, T.; Chen, L.C.; Kinney, P.; Falcone, G.; Sparer, J.; Beckett, W.S. Characterization of clinical tolerance to inhaled zinc oxide in naive subjects and sheet metal workers. *Journal of Occupational and Environmental Medicine* **2000**, *42*, 1085-1091.

192. Hunting, K.L.; Welch, L.S. Occupational exposure to dust and lung disease among sheet metal workers. *British Journal of Industrial Medicine* **1993**, *50*, 432-442.

193. Coggon, D.; Inskip, H.; Winter, P.; Pannett, B. Lobar pneumonia: an occupational disease in welders. *The Lancet* **1994**, *344*, 41-43.

194. Chmielewski, J.; Jaremin, B.; Bartnicki, C.; Konieczka, R. Evaluation of occupational exposure to zinc oxide in the marine production shipyard. II. Examination of the state of health of the workers exposed to zinc oxide. *BIUL.INST.MED.MORSK.GDANSKY* **1974**, *25*, 53-65.

195. Chmielewski, J.; Jaremin, B.; Bartnicki, C.; Konieczka, R. Evaluation of occupational exposure to zinc oxide in the marine production shipyard. I. Examination of the working environment and the stands under exposure. *BIUL.INST.MED.MORSK.GDANSKY* **1974**, *25*, 43-51.

196. Thomas, T.L.; Stolley, P.D.; Stemhagen, A.; Fontham, E.T.; Bleecker, M.L.; Stewart, P.A.; Hoover, R.N. Brain tumor mortality risk among men with electrical and electronics jobs: A case-control study. *Journal of the National Cancer Institute* **1987**, *79*, 233-238.

197. Oliveira, M.; Capelas, S.; Delerue-Matos, C.; Pereira, I.B.; Morais, S. Barbecue grill workers occupational exposure to particulate-bound polycyclic aromatic hydrocarbons. In *Studies in Systems, Decision and Control*, 2019; Vol. 202, pp 201-209.

198. Jørgensen, R.B.; Strandberg, B.; Sjaastad, A.K.; Johansen, A.; Svendsen, K. Simulated restaurant cook exposure to emissions of PAHs, mutagenic aldehydes, and particles from frying bacon. *Journal of Occupational and Environmental Hygiene* **2013**, *10*, 122-131.

199. Svendsen, K.; Sjaastad, A.K.; Sivertsen, I. Respiratory symptoms in kitchen workers. *American Journal of Industrial Medicine* **2003**, *43*, 436-439.

200. Sivertsen, I.; Sjaastad, A.K.; Svendsen, K.; Krøkje, Å. Alveolar macrophages as biomarkers of pulmonary irritation in kitchen workers. *Annals of Occupational Hygiene* **2002**, *46*, 713-717.

201. Svendsen, K.; Jensen, H.N.; Sivertsen, I.; Sjaastad, A.K. Exposure to cooking fumes in restaurant kitchens in Norway. *Annals of Occupational Hygiene* **2002**, *46*, 395-400.

202. Thiébaud, H.P.; Knize, M.G.; Kuzmicky, P.A.; Hsieh, D.P.; Felton, J.S. Airborne mutagens produced by frying beef, pork and a soy-based food. *Food and Chemical Toxicology* **1995**, *33*, 821-828.

203. Löfroth, G. Airborne mutagens and carcinogens from cooking and other food preparation processes. *Toxicology Letters* **1994**, *72*, 83-86.

204. Wardle, E.N. Alveolar cell carcinoma in a cook. *British Journal of Clinical Practice* **1988**, *42*, 173-174.

205. Svedahl, S.R.; Hilt, B.; Svendsen, K. Work environment factors and respiratory complaints in Norwegian cooks. *Int Arch Occup Environ Health* **2019**, 10.1007/s00420-019-01473-w.

206. Goel, A.; Ola, D.; Veetil, A.V. Burden of disease for workers attributable to exposure through inhalation of PPAHs in RSPM from cooking fumes. *Environ Sci Pollut Res Int* **2019**, *26*, 8885-8894.

207. Bigert, C.; Lonn, M.; Feychting, M.; Sjogren, B.; Lewne, M.; Gustavsson, P. Incidence of myocardial infarction among cooks and other restaurant workers in Sweden 1987-2005. *Scand J Work Environ Health* **2013**, *39*, 204-211.

208. Wu, P.-F.; Chiang, T.-A.; Wang, L.-F.; Chang, C.-S.; Ko, Y.-C. Nitro-polycyclic aromatic hydrocarbon contents of fumes from heated cooking oils and prevention of mutagenicity by catechin. *Mutation Research/Fundamental and Molecular Mechanisms of Mutagenesis* **1998**, *403*, 29-34.

209. Borander, A.K.; Voie, Ø.A.; Longva, K.; Danielsen, T.E.; Grahnstedt, S.; Sandvik, L.; Kongerud, J.; Sikkeland, L.I.B. Military small arms fire in association with acute decrements in lung function. *Occupational and Environmental Medicine* **2017**, *74*, 639-644.

210. Gray, G.C.; Reed, R.J.; Kaiser, K.S.; Smith, T.C.; Gastañaga, V.M. Self-reported symptoms and medical conditions among 11,868 Gulf War-era veterans: The Seabee health study. *American Journal of Epidemiology* **2002**, *155*, 1033-1044.

211. Garcia, J.M. Breathing in. *Virginia Quarterly Review* **2011**, *87*, 96-113.

212. Eng, A.; T Mannetje, A.; Cheng, S.; Douwes, J.; Ellison-Loschmann, L.; McLean, D.; Gander, P.; Laird, I.; Legg, S.; Pearce, N. The New Zealand workforce survey I: Self-reported occupational exposures. *Annals of Occupational Hygiene* **2010**, *54*, 144-153.

213. Promisloff, R.A.; Phan, A.; Lenchner, G.S.; Cichelli, A.V. Reactive airway dysfunction syndrome in three police officers following a roadside chemical spill. *Chest* **1990**, *98*, 928-929.

214. Demarini, D.M. Genotoxicity biomarkers associated with exposure to traffic and near-road atmospheres: A review. *Mutagenesis* **2013**, *28*, 485-505.

215. Richiardi, L.; Mirabelli, D.; Calisti, R.; Ottino, A.; Ferrando, A.; Boffetta, P.; Merletti, F. Occupational exposure to diesel exhausts and risk for lung cancer in a population-based case-control study in Italy. *Annals of Oncology* **2006**, *17*, 1842-1847.

216. León-Mejía, G.; Luna-Rodríguez, I.; Trindade, C.; Oliveros-Ortíz, L.; Anaya-Romero, M.; Luna-Carrascal, J.; Navarro-Ojeda, N.; Ruiz-Benitez, M.; Franco-Valencia, K.; Da Silva, J., et al. Cytotoxic and genotoxic effects in mechanics occupationally exposed to diesel engine exhaust. *Ecotoxicology and Environmental Safety* **2019**, *171*, 264-273.

217. Peters, S.; Carey, R.N.; Driscoll, T.R.; Glass, D.C.; Benke, G.; Reid, A.; Fritschi, L. The Australian Work Exposures Study: prevalence of occupational exposure to diesel engine exhaust. *Ann Occup Hyg* **2015**, *59*, 600-608.

218. Pronk, A.; Coble, J.; Stewart, P.A. Occupational exposure to diesel engine exhaust: a literature review. *J Expo Sci Environ Epidemiol* **2009**, *19*, 443-457.

219. Ramachandran, G.; Paulsen, D.; Watts, W.; Kittelson, D. Mass, surface area and number metrics in diesel occupational exposure assessment. *J Environ Monit* **2005**, *7*, 728-735.

220. Muscat, J.E.; Wynder, E.L. Diesel engine exhaust and lung cancer: an unproven association. *Environmental Health Perspectives* **1995**, *103*, 812-818.

221. Chattopadhyay, B.P.; Alam, J.; Roychowdhury, A.J.L.V. Pulmonary Function Abnormalities Associated with Exposure to Automobile Exhaust in a Diesel Bus Garage and Roads. **2003**, 291-302.

222. Wortley, P.; Vaughan, T.L.; Davis, S.; Morgan, M.S.; Thomas, D.B. A case-control study of occupational risk factors for laryngeal cancer. *British journal of industrial medicine* **1992**, *49*, 837-844.

223. Zaebst, D.D.; Clapp, D.E.; Blade, L.M.; Marlow, D.A.; Steenland, K.; Hornung, R.W.; Scheutzle, D.; Butler, J. QUANTITATIVE DETERMINATION OF TRUCKING INDUSTRY WORKERS' EXPOSURES TO DIESEL EXHAUST PARTICLES. *American Industrial Hygiene Association Journal* **1991**, *52*, 529-541.

224. Willems, M.I.; de Raat, W.K.; Wesstra, J.A.; Bakker, G.L.; Dubois, G.; van Dokkum, W. Urinary and faecal mutagenicity in car mechanics exposed to diesel exhaust and in unexposed office workers. *Mutation Research/Genetic Toxicology* **1989**, *222*, 375-391.

225. Gillies, A.D.S.; Wu, H.W. Some approaches and methods for real-time DPM ambient monitoring in underground mines. pp. 465-470.

226. Rosengren, L.; Aminossadati, S.M. Investigation of a management system for the control of diesel emissions in an underground coal mine. *Journal of the Mine Ventilation Society of South Africa* **2011**, *64*, 17-23.

227. Tien, J.C. Coal mine ventilation practices in the United States. pp. 79-82.

228. Aziz, N.I.; Cram, K. Safety and welfare of Australian black coal mine employees. *Journal of Mines, Metals and Fuels* **2001**, *49*.

229. Schulz, H.M.; Hagemann, H.W.; Wolf, M.; Brammertz, A.; Einbrodt, H.J. Coal mine workers' pneumoconiosis. *Environmental Geology* **1997**, *30*, 72-80.

230. Haney, R.A.; Saseen, G.P.; Waytulonis, R.W. An overview of diesel particulate exposures and control technology in the u.S. mining industry. *Applied Occupational and Environmental Hygiene* **1997**, *12*, 1013-1018.

231. Pratt, S.L.; Grainger, A.P.; Todd, J.; Meena, G.G.; Rogers, A.J.; Davies, B. Evaluation and control of employee exposure to diesel particulate at several australian coal mines. *Applied Occupational and Environmental Hygiene* **1997**, *12*, 1032-1037.

232. Qu, S.X.; Leigh, J.; Koelmeyer, H.; Stacey, N.H. DNA adducts in coal miners: Association with exposures to diesel engine emissions. *Biomarkers* **1997**, *2*, 95-102.

233. Carlson, D.H.; Johnson, J.H.; Bagley, S.T.; Gratz, L.D. Underground coal mine air quality in mines using disposable diesel exhaust filter control devices. *Applied Occupational and Environmental Hygiene* **1996**, *11*, 703-706.

234. Suhartono, L.; Cornilsen, B.C.; Johnson, J.H.; Carlson, D.H. Quantitative measurement of diesel particulate matter in an underground coal mine using laser Raman spectroscopy. *Applied Occupational and Environmental Hygiene* **1996**, *11*, 790-798.

235. Wirth, G.J.; Schultz, M.J.; Francart, W.J. Use of atmospheric monitoring systems in dieselized coal mines. pp. 423-427.

236. Tomb, T.F.; Haney, R.A. Results of underground mine studies to assess diesel particulate exposures and control technologies. *Mining Engineering* **1995**, *47*, 276-279.

237. Cantrel, B.K.; Rubow, K.L.; Watts, W.F. Pollutant levels in underground coal mines using diesel equipment. pp. 59-64.

238. Haney, R.A. Diesel particulate exposures in underground mines. *Mining Engineering* **1992**, *44*, 173-176.

239. Waytulonis, K.W. Emission control options for mine diesels. *Mining Engineering* **1991**, *43*, 325-328.

240. Watts, W.F.; Waytulonis, R.W. Why is diesel particulate in mines an issue and how can it be controlled. pp. 247-253.

241. Wheeler, R.W.; Hearl, F.J.; McCawley, M. An industrial hygiene characterization of exposure to diesel emissions in an underground coal mine. *Environment international* **1981**, *5*, 485-488.

242. Hartman, H.L.; Novak, T.; Gregg, A.J. Health hazards of diesel and electric vehicles in an underground coal mine. *Mining Science and Technology* **1987**, *5*, 131-151.

243. Ames, R.G.; Reger, R.B.; Hall, D.S. Chronic respiratory effects of exposure to diesel emissions in coal mines. *Archives of Environmental Health* **1984**, *39*, 389-394.

244. Cullinan, P. Occupation and chronic obstructive pulmonary disease (COPD). *British Medical Bulletin* **2012**, *104*, 143-161.

245. Hoffmann, B.; Jöckel, K.H. Diesel exhaust and coal mine dust: Lung cancer risk in occupational settings. In *Annals of the New York Academy of Sciences*, 2006; Vol. 1076, pp 253-265.

246. Kaplan, I. Relationship of noxious gases to carcinoma of the lung in railroad workers. *Journal of the American Medical Association* **1959**, *171*, 2039-2043.

247. Oliver, L.C.; Miracle-McMahill, H.; Littman, A.B.; Oakes, J.M.; Gaita Jr, R.R. Respiratory symptoms and lung function in workers in heavy and highway construction: A cross-sectional study*. *American Journal of Industrial Medicine* **2001**, *40*, 73-86.

248. Choi, S.; Park, J.-H.; Kim, S.-Y.; Kwak, H.; Kim, D.; Lee, K.-H.; Park, D.-U. Characteristics of PM(2.5) and Black Carbon Exposure Among Subway Workers. *International journal of environmental research and public health* **2019**, *16*, 2901.

249. Tompa, A.; Jakab, M.G.; Biró, A.; Magyar, B.; Major, J. Health, genotoxicology, and immune status of road pavers in Hungary. *Journal of Occupational and Environmental Hygiene* **2007**, *4*, 154-162.

250. Kespohl, S.; Kotschy-Lang, N.; Tomm, J.M.; Von Bergen, M.; Maryska, S.; Brüning, T.; Raulf-Heimsoth, M. Occupational IgE-mediated softwood allergy: Characterization of the causative allergen. *International Archives of Allergy and Immunology* **2012**, *157*, 202-208.

251. Álvarez Eire, M.; Pineda, F.; Varela Losada, S.; González De La Cuesta, C.; Menéndez Villalva, M. Occupational rhinitis and asthma due to cedroarana (Cedrelinga catenaeformis Ducke) wood dust allergy. *Journal of Investigational Allergology and Clinical Immunology* **2006**, *16*, 385-387.

252. Bussi, M.; Gervasio, C.F.; Riontino, E.; Valente, G.; Ferrari, L.; Pira, E.; Cortesina, G. Study of ethmoidal mucosa in a population at occupational high risk of sinonasal adenocarcinoma. *Acta Oto-Laryngologica* **2002**, *122*, 197-201.

253. Minov, J.; Karadzinska-Bislimovska, J.; Tutkun, E.; Vasilevska, K.; Risteska-Kuc, S.; Stoleski, S.; Mijakoski, D. Chronic obstructive pulmonary disease in never-smoking female workers exposed to cotton dust. *Macedonian Journal of Medical Sciences* **2014**, *7*, 316-322.

254. Lai, P.S.; Christiani, D.C. Long-term respiratory health effects in textile workers. *Current Opinion in Pulmonary Medicine* **2013**, *19*, 152-157.

255. Beshir, S.; Mahdy-Abdallah, H.; Saad-Hussein, A. Ventilatory functions in cotton textile workers and the role of some inflammatory cytokines. *Toxicology and Industrial Health* **2013**, *29*, 114-120.

256. Rushton, L. Occupational causes of chronic obstructive pulmonary disease. *Reviews on Environmental Health* **2007**, *22*, 195-212.

257. Mastrangelo, G.; Tartari, M.; Fedeli, U.; Fadda, E.; Saia, B. Ascertaining the risk of chronic obstructive pulmonary disease in relation to occupation using a case-control design. *Occupational Medicine* **2003**, *53*, 165-172.

258. Luce, D.; Gérin, M.; Morcet, J.F.; Leclerc, A. Sinonasal cancer and occupational exposure to textile dust. *American Journal of Industrial Medicine* **1997**, *32*, 205-210.

259. Comba, P.; Battista, G.; Belli, S.; De Capua, B.; Merler, E.; Orsi, D.; Rodella, S.; Vindigni, C.; Axelson, O. A case‐control study of cancer of the nose and paranasal sinuses and occupational exposures. *American Journal of Industrial Medicine* **1992**, *22*, 511-520.

260. Minov, J.; Karadžinska-Bislimovska, J.; Vasilevska, K.; Risteska-Kuč, S.; Stoleski, S. Exercise-induced bronchoconstriction in textile and agricultural workers and in bakers. *Arhiv za Higijenu Rada i Toksikologiju* **2006**, *57*, 379-386.

261. Aidoo, H.; Beach, J.; Elbourne, R.; Galarneau, J.-M.F.; Straube, S.; Cherry, N. Estimation and Validation of Flour Exposure in Bakeries in Alberta, Canada. *Annals of Work Exposures and Health* **2018**, *62*, 1096-1108.

262. Raulf-Heimsoth, M.; Sander, I.; Kespohl, S.; van Kampen, V.; Brüning, T. Rare and new occupational inhalant allergens. *Allergol Select* **2017**, *1*, 65-70.

263. Kirkeleit, J.; Hollund, B.E.; Riise, T.; Eduard, W.; Bråtveit, M.; Storaas, T. Bakers' exposure to flour dust. *Journal of Occupational and Environmental Hygiene* **2017**, *14*, 81-91.

264. Bittner, C.; Garrido, M.V.; Harth, V.; Preisser, A.M. IgE Reactivity, Work Related Allergic Symptoms, Asthma Severity, and Quality of Life in Bakers with Occupational Asthma. In *Allergy and Respiration*, Pokorski, M., Ed. Springer International Publishing: Cham, 2016; 10.1007/5584_2016_226pp. 51-60.

265. Raulf, M. Allergen component analysis as a tool in the diagnosis of occupational allergy. *Current Opinion in Allergy and Clinical Immunology* **2016**, *16*, 93-100.

266. Stobnicka, A.; Górny, R.L. Exposure to flour dust in the occupational environment. *Int J Occup Saf Ergon* **2015**, *21*, 241-249.

267. Saad-Hussein, A.; Taha, M.M.; Fadl, N.N.; Awad, A.H.; Mahdy-Abdallah, H.; Moubarz, G.; Aziz, H.; El-Shamy, K.A. Effects of airborne Aspergillus on serum aflatoxin B1 and liver enzymes in workers handling wheat flour. *Human & Experimental Toxicology* **2015**, *35*, 3-9.

268. Simonis, B.; Hölzel, C.; Stark, U. Glucoamylase: a current allergen in the baking industry. *Allergo J Int* **2014**, *23*, 269-273.

269. Fahim, A.E.; El-Prince, M. Pulmonary function impairment and airway allergy among workers in traditional bakeries. *Int J Occup Med Environ Health* **2013**, *26*, 214-219.

270. Rémen, T.; Acouetey, D.-S.; Paris, C.; Zmirou-Navier, D. Diet, occupational exposure and early asthma incidence among bakers, pastry makers and hairdressers. *BMC public health* **2012**, *12*, 387-387.

271. Fishwick, D.; Harris-Roberts, J.; Robinson, E.; Evans, G.; Barraclough, R.; Sen, D.; Curran, A.D. Impact of worker education on respiratory symptoms and sensitization in bakeries. *Occupational Medicine* **2011**, *61*, 321-327.

272. Meijster, T.; Warren, N.; Heederik, D.; Tielemans, E. What is the best strategy to reduce the burden of occupational asthma and allergy in bakers? **2011**, *68*, 176-182.

273. Houba, R.; Heederik, D.; Doekes, G. Wheat Sensitization and Work-related Symptoms in the Baking Industry Are Preventable. *American Journal of Respiratory and Critical Care Medicine* **1998**, *158*, 1499-1503.

274. Meijster, T.; Tielemans, E.; Heederik, D. Effect of an intervention aimed at reducing the risk of allergic respiratory disease in bakers: change in flour dust and fungal alpha-amylase levels. **2009**, *66*, 543-549.

275. Harris-Roberts, J.; Robinson, E.; Waterhouse, J.C.; Billings, C.G.; Proctor, A.R.; Stocks-Greaves, M.; Rahman, S.; Evans, G.; Garrod, A.; Curran, A.D., et al. Sensitization to wheat flour and enzymes and associated respiratory symptoms in British bakers. *American Journal of Industrial Medicine* **2009**, *52*, 133-140.

276. Laurière, M.; Gorner, P.; Bouchez-Mahiout, I.; Wrobel, R.; Breton, C.; Fabriès, J.-F.; Choudat, D. Physical and Biochemical Properties of Airborne Flour Particles Involved in Occupational Asthma. *The Annals of Occupational Hygiene* **2008**, *52*, 727-737.

277. Burstyn, I.; Teschke, K.; Kennedy, S.M. Exposure levels and determinants of inhalable dust exposure in bakeries. *The Annals of Occupational Hygiene* **1997**, *41*, 609-624.

278. Jacobs, J.H.; Meijster, T.; Meijer, E.; Suarthana, E.; Heederik, D. Wheat allergen exposure and the prevalence of work-related sensitization and allergy in bakery workers. *Allergy* **2008**, *63*, 1597-1604.

279. Mounier-Geyssant, E.; Barthélemy, J.-F.; Mouchot, L.; Paris, C.; Zmirou-Navier, D. Exposure of bakery and pastry apprentices to airborne flour dust using PM2.5 and PM10personal samplers. *BMC Public Health* **2007**, *7*, 311.

280. Stuurman, B.; Meijster, T.; Heederik, D.; Doekes, G. Inhalable β(1→3)glucans as a non-allergenic exposure factor in Dutch bakeries. **2008**, *65*, 68-70.

281. Renström, A.; Mattsson, M.-L.; Blidberg, K.; Doekes, G.; Bogdanovic, J.; Tovey, E. Nasal Air Sampling for Measuring Inhaled Wheat Allergen in Bakeries With and Without Facemask Use. **2006**, *48*, 948-954.

282. Peretz, C.; de Pater, N.; de Monchy, J.; Oostenbrink, J.; Heederik, D. Assessment of exposure to wheat flour and the shape of its relationship with specific sensitization. *Scandinavian Journal of Work, Environment & Health* **2005**, 10.5271/sjweh.850, 65-74.

283. Brant, A.; Berriman, J.; Sharp, C.; Welch, J.; Zekveld, C.; Nieuwenhuijsen, M.; Elms, J.; Newman-Taylor, A.; Cullinan, P. The changing distribution of occupational asthma: a survey of supermarket bakery workers. **2005**, *25*, 303-308.

284. Brisman, J.; Nieuwenhuijsen, M.J.; Venables, K.M.; Putcha, V.; Gordon, S.; Taylor, A.J.N. Exposure-response relations for work related respiratory symptoms and sensitisation in a cohort exposed to α-amylase. **2004**, *61*, 551-553.

285. Smith, T.A. Preventing baker's asthma: an alternative strategy. *Occupational Medicine* **2004**, *54*, 21-27.

286. Elms, J.; Beckett, P.; Griffin, P.; Evans, P.; Sams, C.; Roff, M.; Curran, A.D. Job Categories and Their Effect on Exposure to Fungal Alpha-Amylase and Inhalable Dust in the U.K. Baking Industry. *AIHA Journal* **2003**, *64*, 467-471.

287. Talini, D.; Benvenuti, A.; Carrara, M.; Vaghetti, E.; Martini, L.B.; Paggiaro, P.L. Diagnosis of flour-induced occupational asthma in a cross-sectional study. *Respiratory Medicine* **2002**, *96*, 236-243.

288. Quirce; Polo; Figueredo; González; Sastre. Occupational asthma caused by soybean flour in bakers—differences with soybean-induced epidemic asthma. *Clinical & Experimental Allergy* **2000**, *30*, 839-846.

289. Brisman, J.; Järvholm, B.; Lillienberg, L. Exposure-response relations for self reported asthma and rhinitis in bakers. **2000**, *57*, 335-340.

290. Smith, T.A.; Smith, P.W. Respiratory symptoms and sensitization in bread and cake bakers. *Occupational Medicine* **1998**, *48*, 321-328.

291. Brisman, J.; Torén, K.; Lillienberg, L.; Karlsson, G.; Ahlstedt, S. Nasal symptoms and indices of nasal inflammation in flour-dust-exposed bakers. *International Archives of Occupational and Environmental Health* **1998**, *71*, 525-532.

292. Burstyn, I.; Teschke, K.; Bartlett, K.; Kennedy, S.M. Determinants of Wheat Antigen and Fungal α-Amylase Exposure in Bakeries. *American Industrial Hygiene Association Journal* **1998**, *59*, 313-320.

293. Houba, R.; van Run, P.; Doekes, G.; Heederik, D.; Spithoven, J. Airborne levels of α-amylase allergens in bakeries. *Journal of Allergy and Clinical Immunology* **1997**, *99*, 286-292.

294. Kolopp-Sarda, M.N.; Bene, M.C.; Massin, N.; Wild, P.; Faure, G.C. Altered partition of t cell subsets in the peripheral blood of healthy workers exposed to flour dust. *American Journal of Industrial Medicine* **1995**, *28*, 497-504.

295. Merget, R.; Heger, M.; Globisch, A.; Rasche, K.; Gillissen, A.; Gebler, A.; Schultze-Werninghaus, G.; Krieg, M. Quantitative bronchial challenge tests with wheat flour dust administered by Spinhaler: Comparison with aqueous wheat flour extract inhalation. *Journal of Allergy and Clinical Immunology* **1997**, *100*, 199-207.

296. Wiley, K.; Smith, M.M.; Allan, L.J.; Griffin, P. Measurement of Airborne Flour Exposure with a Monoclonal Antibody-Based Immunoassay. *International Archives of Allergy and Immunology* **1997**, *114*, 278-284.

297. Bolund, A.C.S.; Miller, M.R.; Jacobsen, G.H.; Sigsgaard, T.; Schlünssen, V. New-onset COPD and Decline in Lung Function Among Wood Dust-Exposed Workers: Re-analysis of a 6-year Follow-up Study. *Annals of work exposures and health* **2018**, *62*, 1064-1076.

298. Martin, J.; Vayr, F.; Paris, C.; Vergez, S.; Krief, P.; Luc, A.; Corvisier, J.; de Gabory, L.; Herin, F.; members, C.E. Nasal fibroscopy as a routine screening procedure of sinonasal adenocarcinoma of woodworkers: French longitudinal case study. *Head and Neck* **2018**, *40*, 2193-2198.

299. Douwes, J.; Cheung, K.; Prezant, B.; Sharp, M.; Corbin, M.; McLean, D.; T Mannetje, A.; Schlunssen, V.; Sigsgaard, T.; Kromhout, H., et al. Wood dust in joineries and furniture manufacturing: An exposure determinant and intervention study. *Annals of Work Exposures and Health* **2017**, *61*, 416-428.

300. Rourke, T.; Grover, S.; Wager, N.; Capper, J. Decreasing incidence of nasal adenocarcinoma in Wycombe woodworkers. *The Laryngoscope* **2014**, *124*, 1078-1082.

301. Dostbil, Z.; Polat, C.; Uysal, I.Ö.; Bakır, S.; Karakuş, A.; Altındağ, S. Evaluation of Nasal Mucociliary Transport Rate byTc-Macroaggregated Albumin Rhinoscintigraphy in Woodworkers. *Int J Mol Imaging* **2011**, *2011*, 620482-620482.

302. Whiteside, O.J.H.; Corbridge, R.J.; Capper, J.W.R. Esme Hadfield (1921-92) and the Wycombe woodworkers. *Journal of Medical Biography* **2010**, *18*, 24-26.

303. Jacobsen, G.; Schlünssen, V.; Schaumburg, I.; Sigsgaard, T. Increased incidence of respiratory symptoms among female woodworkers exposed to dry wood. *European Respiratory Journal* **2009**, *33*, 1268-1276.

304. Schlünssen, V.; Jacobsen, G.; Erlandsen, M.; Mikkelsen, A.B.; Schaumburg, I.; Sigsgaard, T. Determinants of wood dust exposure in the Danish furniture industry - Results from two cross-sectional studies 6 years apart. *Annals of Occupational Hygiene* **2008**, *52*, 227-238.

305. Jacobsen, G.; Schlünssen, V.; Schaumburg, I.; Taudorf, E.; Sigsgaard, T. Longitudinal lung function decline and wood dust exposure in the furniture industry. *European Respiratory Journal* **2008**, *31*, 334-342.

306. Schlünssen, V.; Schaumburg, I.; Heederik, D.; Taudorf, E.; Sigsgaard, T. Indices of asthma among atopic and non-atopic woodworkers. *Occupational and Environmental Medicine* **2004**, *61*, 504-511.

307. Schlünssen, V.; Schaumburg, I.; Taudorf, E.; Mikkelsen, A.B.; Sigsgaard, T. Respiratory symptoms and lung function among danish woodworkers. *Journal of Occupational and Environmental Medicine* **2002**, *44*, 82-98.

308. Ahman, M.; Holmstrom, M. Nasal histamine reactivity in woodwork teachers. *Rhinology* **2000**, *38*, 114-119.

309. Blot, W.J.; Chow, W.H.; McLaughlin, J.K. Wood dust and nasal cancer risk: A review of the evidence from North America. *Journal of Occupational and Environmental Medicine* **1997**, *39*, 148-156.

310. Pisaniello, D.L.; Connell, K.E.; Muriale, L. WOOD DUST EXPOSURE DURING FURNITURE MANUFACTURE—RESULTS FROM AN AUSTRALIAN SURVEY AND CONSIDERATIONS FOR THRESHOLD LIMIT VALUE DEVELOPMENT. *American Industrial Hygiene Association Journal* **1991**, *52*, 485-492.

311. Boysen, M.; Voss, R.; Solberg, L.A. The nasal mucosa in softwood exposed furniture workers. *Acta Oto-Laryngologica* **1986**, *101*, 501-508.

312. Cellai, F.; Capacci, F.; Sgarrella, C.; Poli, C.; Arena, L.; Tofani, L.; Giese, R.W.; Peluso, M. A cross-sectional study on 3-(2-deoxy-β-D-erythro-pentafuranosyl)pyrimido [1,2-α]purin-10(3H)-one deoxyguanosine adducts among woodworkers in Tuscany, Italy. *International Journal of Molecular Sciences* **2019**, *20*.

313. Lovato, A.; Staffieri, C.; Ottaviano, G.; Cappellesso, R.; Giacomelli, L.; Bartolucci, G.B.; Scapellato, M.L.; Marioni, G. Woodworkers and the inflammatory effects of softwood/hardwood dust: evidence from nasal cytology. *European Archives of Oto-Rhino-Laryngology* **2016**, *273*, 3195-3200.

314. Staffieri, C.; Lovato, A.; Aielli, F.; Bortoletto, M.; Giacomelli, L.; Carrieri, M.; Romeo, S.; Boscolo-Rizzo, P.; Da Mosto, M.C.; Bartolucci, G.B., et al. Investigating nasal cytology as a potential tool for diagnosing occupational rhinitis in woodworkers. *International Forum of Allergy and Rhinology* **2015**, *5*, 814-819.

315. Pukkala, E.; Martinsen, J.I.; Lynge, E.; Gunnarsdottir, H.K.; Sparn, P.; Tryggvadottir, L.; Weiderpass, E.; Kjaerheim, K. Occupation and cancer follow-up of 15 million people in five Nordic countries. *Acta Oncologica* **2009**, *48*, 646-790.

316. Heikkilä, P.; Martikainen, R.; Kurppa, K.; Husgafvel-Pursiainen, K.; Karjalainen, A. Asthma incidence in wood-processing industries in Finland in a registerbased population study. *Scandinavian Journal of Work, Environment and Health, Supplement* **2008**, *34*, 66-72.

317. Valente, G.; Ferrari, L.; Kerim, S.; Gervasio, C.F.; Ricci, E.; Migliaretti, G.; Pira, E.; Bussi, M. Evidence of p53 immunohistochemical overexpression in ethmoidal mucosa of woodworkers. *Cancer Detection and Prevention* **2004**, *28*, 99-106.

318. Magnavita, N.; Sacco, A.; Bevilacqua, L.; D'Alessandris, T.; Bosman, C. Aesthesioneuroblastoma in a woodworker. *Occupational Medicine* **2003**, *53*, 231-234.

319. Schlünssen, V.; Schaumburg, I.; Andersen, N.T.; Sigsgaard, T.; Pedersen, O.F. Nasal patency is related to dust exposure in woodworkers. *Occupational and Environmental Medicine* **2002**, *59*, 23-29.

320. Palus, J.; Dziubałtowska, E.; Rydzyński, K. DNA damage detected by the comet assay in the white blood cells of workers in a wooden furniture plant. *Mutation Research - Genetic Toxicology and Environmental Mutagenesis* **1999**, *444*, 61-74.

321. Mandryk, J.; Alwis, K.U.; Hocking, A.D. Work-related symptoms and dose-response relationships for personal exposures and pulmonary function among woodworkers. *American Journal of Industrial Medicine* **1999**, *35*, 481-490.

322. Rabone, S.J.; Saraswati, S.B. Acceptance and effects of nasal lavage in volunteer woodworkers. *Occupational Medicine* **1999**, *49*, 365-369.

323. Demers, P.A.; Stellman, S.D.; Colin, D.; Boffetta, P. Nonmalignant respiratory disease mortality among woodworkers participating in the American Cancer Society Cancer Prevention Study-II (CPS- II). *American Journal of Industrial Medicine* **1998**, *34*, 238-243.

324. Talini, D.; Monteverdi, A.; Benvenuti, A.; Petrozzino, M.; Di Pede, F.; Lemmi, M.; Carletti, A.; Macchioni, P.; Serretti, N.; Viegi, G., et al. Asthma-like symptoms, atopy, and bronchial responsiveness in furniture workers. *Occupational and Environmental Medicine* **1998**, *55*, 786-791.

325. Kauppinen, T.P.; Partanen, T.J.; Hernberg, S.G.; Nickels, J.I.; Luukkonen, R.A.; Hakulinen, T.R.; Pukkala, E.I. Chemical exposures and respiratory cancer among Finnish woodworkers. *British Journal of Industrial Medicine* **1993**, *50*, 143-148.

326. Mohtashamipur, E.; Norpoth, K.; Lühmann, F. Cancer epidemiology of woodworking. *Journal of Cancer Research and Clinical Oncology* **1989**, *115*, 503-515.

327. Barthel, E.; Dietrich, M. Retrospective cohor study of cancer morbidity in furniture makers exposed to wood dust. *Zeitschrift fur die Gesamte Hygiene und Ihre Grenzgebiete* **1989**, *35*, 279-281.

328. Wills, J.H. Nasal carcinoma in woodworkers: A review. *Journal of Occupational Medicine* **1982**, *24*, 526-530.

329. Cecchi, F.; Buiatti, E.; Kriebel, D.; Nastasi, L.; Santucci, M. Adenocarcinoma of the nose and paranasal sinuses in shoemakers and woodworkers in the province of Florence, Italy (1963-77). *British Journal of Industrial Medicine* **1980**, *37*, 222-225.

330. Black, A.; Evans, J.C.; Hadfield, E.H.; Macbeth, R.G.; Morgan, A.; Walsh, M. Impairment of nasal mucociliary clearance in woodworkers in the furniture industry. *British Journal of Industrial Medicine* **1974**, *31*, 10-17.

331. Acheson, E.D.; Cowdell, R.H.; Hadfield, E.; Macbeth, R.G. Nasal Cancer in Woodworkers in the Furniture Industry. *British Medical Journal* **1968**, *2*, 587-596.

332. Wilhelmsson, B.; Lundh, B. Nasal epithelium in woodworkers in the furniture industry a histological and cytological study. *Acta Oto-Laryngologica* **1984**, *98*, 321-334.

333. Andersen, H.C.; Solgaard, J.; Andersen, I. Nasal cancer and nasal mucus-transport rates in woodworkers. *Acta Oto-Laryngologica* **1976**, *82*, 263-265.

334. Green, B.J.; Couch, J.R.; Lemons, A.R.; Burton, N.C.; Victory, K.R.; Nayak, A.P.; Beezhold, D.H. Microbial hazards during harvesting and processing at an outdoor United States cannabis farm. *Journal of Occupational and Environmental Hygiene* **2018**, *15*, 430-440.

335. Viegas, S.; Faísca, V.M.; Dias, H.; Clérigo, A.; Carolino, E.; Viegas, C. Occupational exposure to poultry dust and effects on the respiratory system in workers. *Journal of Toxicology and Environmental Health - Part A: Current Issues* **2013**, *76*, 230-239.

336. Skaug, M.A.; Eduard, W.; Størmer, F.C. Ochratoxin A in airborne dust and fungal conidia. *Mycopathologia* **2001**, *151*, 93-98.

337. Mackiewicz, B. Study on exposure of pig farm workers to bioaerosols, immunologic reactivity and health effects. *Annals of Agricultural and Environmental Medicine* **1998**, *5*, 169-175.

338. May, J.J.; Stallones, L.; Darrow, D.; Pratt, D.S. Organic dust to×icity (pulmonary mycoto×icosis) associated with silo unloading. *Thorax* **1986**, *41*, 919-923.

339. Merchant, J.A. Agricultural exposures to organic dusts. *Occupational medicine (Philadelphia, Pa.)* **1987**, *2*, 409-425.

340. Do Pico, G.A. Hazardous exposure and lung disease among farm workers. *Clinics in Chest Medicine* **1992**, *13*, 311-328.

341. Reckner Olsson, Å.; Skogh, T.; Axelson, O.; Wingren, G. Occupations and exposures in the work environment as determinants for rheumatoid arthritis. *Occupational and Environmental Medicine* **2004**, *61*, 233-238.

342. Crook, B. Aerobiological investigation of occupational respiratory allergy in agriculture in the U.K. *Grana* **1994**, *33*, 81-84.

343. Sheats, M.K.; Davis, K.U.; Poole, J.A. Comparative Review of Asthma in Farmers and Horses. *Current Allergy and Asthma Reports* **2019**, *19*.

344. Basinas, I.; Sigsgaard, T.; Kromhout, H.; Heederik, D.; Wouters, I.M.; Schlünssen, V. A comprehensive review of levels and determinants of personal exposure to dust and endotoxin in livestock farming. *Journal of Exposure Science and Environmental Epidemiology* **2015**, *25*, 123-137.

345. Basinas, I.; Sigsgaard, T.; Bønløkke, J.H.; Andersen, N.T.; Omland, O.; Kromhout, H.; Schlünssen, V. Feedback on Measured Dust Concentrations Reduces Exposure Levels among Farmers. *Annals of Occupational Hygiene* **2016**, *60*, 812-824.

346. Donham, K.J.; Meppelink, S.M.; Kelly, K.M.; Rohlman, D.S. Health Indicators of a Cohort of Midwest Farmers: Health Outcomes of Participants in the Certified Safe Farm Program. *Journal of Agromedicine* **2019**, *24*, 228-238.

347. Solarz, K.; Pająk, C. Risk of exposure of a selected rural population in South Poland to allergenic mites. Part II: acarofauna of farm buildings. *Experimental and Applied Acarology* **2019**, *77*, 387-399.

348. Pfister, H.; Madec, L.; Cann, P.L.; Costet, N.; Chouvet, M.; Jouneau, S.; Vernhet, L. Factors determining the exposure of dairy farmers to thoracic organic dust. *Environmental Research* **2018**, *165*, 286-293.

349. Gautam, R.; Heo, Y.; Lim, G.; Song, E.; Roque, K.; Lee, J.; Kim, Y.; Cho, A.; Shin, S.; Kim, C., et al. Altered immune responses in broiler chicken husbandry workers and their association with endotoxin exposure. *Industrial Health* **2018**, *56*, 10-19.

350. Milanowska, J.; Mackiewicz, B.; Węgorowski, P.; Milanowski, J.; Milanowski, P.; Makara-Studzińska, M. The quality of life of farmers with chronic obstructive pulmonary disease (COPD). *Annals of Agricultural and Environmental Medicine* **2017**, *24*, 283-287.

351. Suzuki, Y.; Imokawa, S.; Nihashi, F.; Uto, T.; Sato, J.; Suda, T. Diffuse alveolar hemorrhage caused by exposure to organic dust. *Respiratory Medicine Case Reports* **2015**, *15*, 59-61.

352. Spierenburg, E.A.J.; Smit, L.A.M.; Heederik, D.; Robbe, P.; Hylkema, M.N.; Wouters, I.M. Healthy worker survivor analysis in an occupational cohort study of Dutch agricultural workers. *International Archives of Occupational and Environmental Health* **2015**, *88*, 1165-1173.

353. Müller-Wening, D.; Neuhauss, M. Protective effect of respiratory devices in farmers with occupational asthma. *European Respiratory Journal* **1998**, *12*, 569-572.

354. Husman, K.; Terho, E.O.; Notkola, V.; Nuutinen, J. Organic dust toxic syndrome among finnish farmers. *American Journal of Industrial Medicine* **1990**, *17*, 79-80.

355. Skórska, C.; Mackiewicz, B.; Dutkiewicz, J.; Krysińska-Traczyk, E.; Milanowski, J.; Feltovich, H.; Lange, J.; Thorne, P.S. Effects of exposure to grain dust in Polish farmers: Work-related symptoms and immunologic response to microbial antigens associated with dust. *Annals of Agricultural and Environmental Medicine* **1998**, *5*, 147-153.

356. Von Essen, S.; Fryzek, J.; Nowakowski, B.; Wampler, M. Respiratory symptoms and farming practices in farmers associated with an acute febrile illness after organic dust exposure. *Chest* **1999**, *116*, 1452-1458.

357. Iversen, M.; Dahl, R. Working in swine-confinement buildings causes an accelerated decline in FEV1: A 7-yr follow-up of Danish farmers. *European Respiratory Journal* **2000**, *16*, 404-408.

358. Monso, E.; Magarolas, R.; Radon, K.; Danuser, B.; Iversen, M.; Weber, C.; Opravil, U.; Donham, K.J.; Nowak, D. Respiratory symptoms of obstructive lung disease in European crop farmers. *American Journal of Respiratory and Critical Care Medicine* **2000**, *162*, 1246-1250.

359. Eduard, W.; Douwes, J.; Mehl, R.; Heederik, D.; Melbostad, E. Short term exposure to airborne microbial agents during farm work: Exposure-response relations with eye and respiratory symptoms. *Occupational and Environmental Medicine* **2001**, *58*, 113-118.

360. Melbostad, E.; Eduard, W. Organic dust-related respiratory and eye irritation in Norwegian farmers*. *American Journal of Industrial Medicine* **2001**, *39*, 209-217.

361. Iversen, M.; Kirychuk, S.; Drost, H.; Jacobson, L. Human health effects of dust exposure in animal confinement buildings. *J Agric Saf Health* **2000**, *6*, 283-288.

362. Radon, K.; Weber, C.; Iversen, M.; Danuser, B.; Pedersen, S.; Nowak, D. Exposure assessment and lung function in pig and poultry farmers. *Occupational and Environmental Medicine* **2001**, *58*, 405-410.

363. Cathomas, R.L.; Brüesch, H.; Fehr, R.; Reinhart, W.H.; Kuhn, M. Organic dust exposure in dairy farmers in an alpine region. *Swiss Medical Weekly* **2002**, *132*, 174-178.

364. Linaker, C.; Smedley, J. Respiratory illness in agricultural workers. *Occupational Medicine* **2002**, *52*, 451-459.

365. Lambert, G.P.; Spurzem, J.R.; Romberger, D.J.; Wyatt, T.A.; Lyden, E.; Stromquist, A.M.; Merchant, J.A.; Von Essen, S.G. Tumor necrosis factor-α hyper-responsiveness to endotoxin in whole blood is associated with chronic bronchitis in farmers. *Journal of Agromedicine* **2005**, *10*, 39-44.

366. Cormier, Y.; Israël-Assayag, E. Chronic inflammation induced by organic dust and related metabolic cardiovascular disease risk factors. *Scandinavian Journal of Work, Environment and Health* **2004**, *30*, 438-444.

367. Di Stefano, F.; Di Giampaolo, L.; Verna, N.; Di Gioacchino, M. Respiratory allergy in agriculture. *European Annals of Allergy and Clinical Immunology* **2007**, *39*, 89-100.

368. Sahlander, K.; Larsson, K.; Palmberg, L. Daily exposure to dust alters innate immunity. *PLoS ONE* **2012**, *7*.

369. May, S.; Romberger, D.J.; Poole, J.A. Respiratory health effects of large animal farming environments. *Journal of Toxicology and Environmental Health - Part B: Critical Reviews* **2012**, *15*, 524-541.

370. Carvalheiro, M.F.; Peterson, Y.; Rylander, R. Bronchial reactivity and work‐related symptoms in farmers. *American Journal of Industrial Medicine* **1995**, *27*, 65-74.

371. Malmberg, P.; Rask-Andersen, A.; Rosenhall, L. Exposure to microorganisms associated with allergic alveolitis and febrile reactions to mold dust in farmers. *Chest* **1993**, *103*, 1202-1209.

372. Axmacher, B.; Axelson, O.; Frodin, T.; Gotthard, R.; Hed, J.; Molin, L.; Noorlind Brage, H.; Strom, M. Dust exposure in coeliac disease: A case-referent study. *British Journal of Industrial Medicine* **1991**, *48*, 715-717.

373. Malmberg, P. Health effects of organic dust exposure in dairy farmers. *American Journal of Industrial Medicine* **1990**, *17*, 7-15.

374. Rask-Andersen, A.; Malmberg, P. Organic dust toxic syndrome in swedish farmers: Symptoms, clinical findings, and exposure in 98 cases. *American Journal of Industrial Medicine* **1990**, *17*, 116-117.

375. Rask-Andersen, A. Organic dust toxic syndrome among farmers. *British Journal of Industrial Medicine* **1989**, *46*, 233-238.

376. Malmberg, P.; Rask-Andersen, A.; Höglund, S.; Kolnwdin-Hedman, B.; Read Guernsey, J. Incidence of organic dust toxic syndrome and allergic alveolitis in swedish farmers. *International Archives of Allergy and Immunology* **1988**, *87*, 47-54.

377. Malmberg, P.; Rask-Andersen, A.; Palmgren, U.; Höglund, S.; Kolmodin-Hedman, B.; Stålenheim, G. Exposure to microorganisms, febrile and airway-obstructive symptoms, immune status and lung function of Swedish farmers. *Scandinavian Journal of Work, Environment and Health* **1985**, *11*, 287-293.

378. Saussereau, J.; Guillien, A.; Soumagne, T.; Laplante, J.J.; Laurent, L.; Bouhaddi, M.; Rocchi, S.; Annesi-Maesano, I.; Roche, N.; Dalphin, J.C., et al. Dietary Patterns and Prevalence of Post-bronchodilator Airway Obstruction in Dairy Farmers Exposed to Organic Dusts. *COPD: Journal of Chronic Obstructive Pulmonary Disease* **2019**, *16*, 118-125.

379. Fontana, L.; Lee, S.J.; Capitanelli, I.; Re, A.; Maniscalco, M.; Mauriello, M.C.; Iavicoli, I. Chronic Obstructive Pulmonary Disease in Farmers: A Systematic Review. *Journal of Occupational and Environmental Medicine* **2017**, *59*, 775-788.

380. Soumagne, T.; Chardon, M.L.; Dournes, G.; Laurent, L.; Degano, B.; Laurent, F.; Dalphin, J.C. Emphysema in active farmer's lung disease. *PLoS ONE* **2017**, *12*.

381. Richardson, D.B.; Terschüren, C.; Hoffmann, W. Occupational risk factors for non-Hodgkin's lymphoma: A population-based case-control study in Northern Germany. *American Journal of Industrial Medicine* **2008**, *51*, 258-268.

382. Halstensen, A.S.; Nordby, K.C.; Wouters, I.M.; Eduard, W. Determinants of microbial exposure in grain farming. *Annals of Occupational Hygiene* **2007**, *51*, 581-592.

383. Schierl, R.; Heise, A.; Egger, U.; Schneider, F.; Eichelser, R.; Neser, S.; Nowak, D. Endotoxin concentration in modern animal houses in Southern Bavaria. *Annals of Agricultural and Environmental Medicine* **2007**, *14*, 129-136.

384. Gainet, M.; Thaon, I.; Westeel, V.; Chaudemanche, H.; Venier, A.G.; Dubiez, A.; Laplante, J.J.; Dalphin, J.C. Twelve-year longitudinal study of respiratory status in dairy farmers. *European Respiratory Journal* **2007**, *30*, 97-103.

385. Sigurdarson, S.T.; Donham, K.J.; Kline, J.N. Acute toxic pneumonitis complicating chronic obstructive pulmonary disease (COPD) in a farmer. *American Journal of Industrial Medicine* **2004**, *46*, 393-395.

386. Mastrangelo, G.; Marzia, V.; Milana, G.; Fadda, E.; Fedeli, U.; Lange, J.H. An Exposure-dependent Reduction of Lung Cancer Risk in Dairy Farmers: A Nested Case-referent Study. *Indoor and Built Environment* **2004**, *13*, 35-43.

387. Vogelzang, P.F.J.; Van Der Gulden, J.W.J.; Folgering, H.; Van Schayck, C.P. Organic dust toxic syndrome in swine confinement farming. *American Journal of Industrial Medicine* **1999**, *35*, 332-334.

388. Edwards, J.H. Organic-dust diseases and endotoxins. *Revue d'Epidemiologie et de Sante Publique* **1981**, *29*, 199-207.

389. Wilhelmsson, J.; Bryngelsson, I.L.; Ohlson, C.G. Respiratory symptoms among swedish swine producers. *American Journal of Industrial Medicine* **1989**, *15*, 311-318.

390. Marx, J.J.; Guernsey, J.; Emanuel, D.A.; Merchant, J.A.; Morgan, D.P.; Kryda, M. Cohort studies of immunologic lung disease among Wisconsin dairy farmers. *American Journal of Industrial Medicine* **1990**, *18*, 263-268.

391. Rylander, R.; Essie, N.; Donham, K.J. Bronchial hyperreactivity among pig and dairy farmers. *American Journal of Industrial Medicine* **1990**, *17*, 66-69.

392. Eduard, W.; Pearce, N.; Douwes, J. Chronic bronchitis, COPD, and lung function in farmers: The role of biological agents. *Chest* **2009**, *136*, 716-725.

393. Golec, M. The effects of long-term occupational exposure to dust from herbs. *International Archives of Occupational and Environmental Health* **2006**, *79*, 169-175.

394. Roy, C.J.; Thorne, P.S. Exposure to Particulates, Microorganisms, β(1–3)-Glucans, and Endotoxins During Soybean Harvesting. *AIHA Journal* **2003**, *64*, 487-495.

395. Dal, M.; Malak, A.T. Effects of SiO2 in turkish natural stones on cancer development. *Asian Pacific Journal of Cancer Prevention* **2012**, *13*, 4883-4888.

396. Freed, J.A.; Miller, A.; Gordon, R.E.; Fischbein, A.; Kleinerman, J.; Langer, A.M. Desquamative interstitial pneumonia associated with chrysotile asbestos fibres. *British Journal of Industrial Medicine* **1991**, *48*, 332-337.

397. van Deurssen, E.; Meijster, T.; Oude Hengel, K.M.; Boessen, R.; Spaan, S.; Tielemans, E.; Heederik, D.; Pronk, A. Effectiveness of a Multidimensional Randomized Control Intervention to Reduce Quartz Exposure Among Construction Workers. *The Annals of Occupational Hygiene* **2015**, *59*, 959-971.

398. Boschman, J.S.; van der Molen, H.F.; Sluiter, J.K.; Frings-Dresen, M.H.W. Occupational demands and health effects for bricklayers and construction supervisors: A systematic review. *American Journal of Industrial Medicine* **2011**, *54*, 55-77.

399. Tjoe Nij, E.; Heederik, D. Risk assessment of silicosis and lung cancer among construction workers exposed to respirable quartz. *Scandinavian Journal of Work, Environment & Health* **2005**, 49-56.

400. Bello, D.; Virji, M.A.; Kalil, A.J.; Woskie, S.R. Quantification of Respirable, Thoracic, and Inhalable Quartz Exposures by FT-IR in Personal Impactor Samples from Construction Sites. *Applied Occupational and Environmental Hygiene* **2002**, *17*, 580-590.

401. Hlavay, J.; Wesemann, G. Distribution of mineralogical phases in dusts collected at different workshops. *Science of the Total Environment, The* **1993**, *136*, 33-42.

402. Partanen, T.; Jaakkola, J.; Tossavainen, A. Silica, silicosis and cancer in Finland. *Scandinavian Journal of Work, Environment & Health* **1995**, 84-86.

403. Andersson, L.; Burdorf, A.; Bryngelsson, I.-L.; Westberg, H. Estimating Trends in Quartz Exposure in Swedish Iron Foundries—Predicting Past and Present Exposures. *The Annals of Occupational Hygiene* **2011**, *56*, 362-372.

404. Kogevinas, M.; Antó, J.M.; Sunyer, J.; Tobias, A.; Kromhout, H.; Burney, P. Occupational asthma in Europe and other industrialised areas: A population-based study. *Lancet* **1999**, *353*, 1750-1754.

405. Liu, Y.C.; Tomashefski, J.; McMahon, J.T.; Petrelli, M. Mineral-associated hepatic injury: A report of seven cases with x-ray microanalysis. *Human Pathology* **1991**, *22*, 1120-1127.

406. Cocco, P.; Palli, D.; Buiatti, E.; Cipriani, F.; DeCarli, A.; Manca, P.; Ward, M.H.; Blot, W.J.; Fraumeni Jr, J.F. Occupational exposures as risk factors for gastric cancer in Italy. *Cancer Causes & Control* **1994**, *5*, 241-248.

407. Pascual del Pobil y Ferré, M.A.; García Sevila, R.; García Rodenas, M.d.M.; Barroso Medel, E.; Flores Reos, E.; Gil Carbonell, J. Silicosis: una antigua enfermedad profesional con nuevos escenarios de exposición laboral. *Revista Clínica Española* **2019**, *219*, 26-29.
